# Characterization of a *β*-Glucosidase from *Fragaria × ananassa*: Substrate Specificity, Catalytic Efficiency, and Structural Insights

**DOI:** 10.3390/foods15183290

**Published:** 2026-09-17

**Authors:** Ibrahim Rabeeah, Alberto Zavarise, Alessandro Manghi, Viktoria Gruber-Schmidt, Matthias Hackl, Renate Paltram, Manfred Gössinger, Luca Cattani, Karl Stich, Hester Sheehan, Heidi Halbwirth, Christian Haselmair-Gosch

**Affiliations:** 1 Research Group Phytochemistry and Biochemistry of Natural Compounds, Institute of Chemical, Environmental and Bioscience Engineering, Technische Universität Wien, 1060 Wien, Austria; 2 Department of Engineering for Industrial Systems and Technologies, University of Parma, Parco Area delle Scienze 181/A, 43124 Parma, Italy; 3 Department of Fruit Processing, Federal College and Institute for Viticulture and Pomology, Wiener Straße 74, 3400 Klosterneuburg, Austria

**Keywords:** *Fragaria × ananassa*, flavonoid glycosides, enzyme kinetics, substrate specificity, molecular docking, fruit metabolism, recombinant expression, *β*-glucosidase

## Abstract

*β*-Glucosidases (*β*GLs) play important roles in plant secondary metabolism by hydrolyzing glycosylated compounds involved in defense, signaling, and the formation of bioactive and flavor-related metabolites. In this study, a *β*-glucosidase from *Fragaria × ananassa* (Fra-*β*GL; *FxaC_10g48920.t1*) was identified and biochemically characterized in comparison with a commercial *β*-glucosidase from *Prunus dulcis* (Pru-*β*GL). The Fra-*β*GL cDNA was cloned and heterologously expressed in *Escherichia coli*, and the recombinant protein was purified for biochemical characterization. Substrate screening demonstrated that Fra-*β*GL hydrolyzed the 7-*O*-glucosides of quercetin, kaempferol, and luteolin, whereas no detectable activity was observed toward the tested 3-*O*-glucosides, anthocyanin glucosides, arbutin, or phloridzin under the assay conditions. Kinetic analysis revealed higher catalytic efficiencies of Fra-*β*GL than Pru-*β*GL toward all four substrates examined. Fra-*β*GL showed its highest turnover rate and catalytic efficiency toward the synthetic substrate *p*-nitrophenyl-*β*-D-glucopyranoside (*p*NP-Glc), with a *K*_cat_ of 60.33 s^−1^ and a *K*_cat_/*K*_m_ of 10.18 mM^−1^·s^−1^. Among the naturally occurring flavonoid glucosides, Fra-*β*GL exhibited the highest catalytic efficiency toward quercetin 7-*O*-glucoside (4.14 mM^−1^·s^−1^), approximately fivefold higher than that of Pru-*β*GL (0.824 mM^−1^·s^−1^). Thermal stability analysis showed progressive loss of activity with increasing temperature, with Pru-*β*GL retaining greater residual activity than Fra-*β*GL at elevated temperatures. Molecular docking suggested structural features that may contribute to the observed preference of Fra-*β*GL for flavonoid 7-*O*-glucosides over the corresponding 3-*O*-glucosides. Collectively, these findings demonstrate the substrate selectivity and distinct catalytic properties of Fra-*β*GL and identify Fra-*β*GL as a candidate for further investigation in the context of flavonoid glycoside metabolism in strawberry.

## 1. Introduction

Secondary metabolites occupy a pivotal role in the physiology and ecological success of strawberries. Unlike primary metabolites, which are indispensable for growth and energy production, secondary metabolites are typically specialized compounds derived from core pathways such as the phenylpropanoid, flavonoid, and terpenoid biosynthetic routes [1,2,3]. In strawberries, these include a rich array of phenolic acids (e.g., ellagic acid derivatives), flavonoids (flavonols, flavan -3-ols), anthocyanins, and tannins, as well as volatile terpenes and esters that strongly shape fruit aroma [4,5]. Each class contributes to distinct biological functions: e.g., flavonoids and anthocyanins act as potent antioxidants and UV protectants [6]; tannins and phenolic glycosides deter herbivory and microbial attack [7]; and terpenoids and volatile esters enhance pollinator attraction and consumer appeal through aroma and flavor [8,9].

From a nutritional perspective, strawberry secondary metabolites are considered key determinants of fruit quality [10]. They contribute not only to visual traits such as color intensity but also to mouthfeel, sweetness perception, and aroma complexity [10,11,12]. Many of these compounds, particularly anthocyanins, ellagitannins, and flavonols, are also linked to health-promoting properties in humans, including anti-inflammatory, cardioprotective, and anticancer activities [13,14]. This dual ecological and nutritional importance makes strawberries a compelling system in which to investigate how secondary metabolites are synthesized, stored, and mobilized during fruit ripening.

In strawberries, many phenolic compounds, including flavonoids and anthocyanins, accumulate in their glycosylated forms, where glycosylation enhances solubility, stability, and intracellular transport [15]. The hydrolysis of these conjugates by *β*GLs can dramatically alter their biological activity, availability, and sensory contribution [16]. Substrates of potential relevance include flavonol glucosides such as kaempferol and quercetin derivatives, flavone glucosides such as luteolin 7-*O*-glucoside, anthocyanin glucosides like cyanidin and pelargonidin 3-*O*-glucoside, and glycosylated phenolics such as phloridzin and arbutin [17]. In addition, synthetic model substrates such as *p*NP-Glc are frequently employed to probe catalytic activity [18,19]. The ability of *β*GLs to recognize and process these structurally diverse compounds highlights their potential role at the intersection of fruit physiology, defense, and sensory attributes.

Plant *β*GLs are encoded by large multigene families, especially within glycoside hydrolase family 1 (GH1), which contain dozens of *β*-glucosidase-like genes in most angiosperms (e.g., ~40 GH1 genes in rice and similar numbers in many species) and are implicated in processes such as defense compound activation, phytohormone release, lignin metabolism, and secondary metabolite turnover [20]. However, only a small number of individual *β*GLs have been biochemically detailed with defined substrates and kinetics [20,21]. GH1 *β*GLs generally have a (*β/α*)_8_-barrel fold and a retaining catalytic mechanism, and their substrate specificities range from narrow (e.g., specific natural glucosides) to moderately broad, acting on structurally diverse glucosides [22].

Abscisic acid (ABA) plays an important role in the regulation of strawberry fruit development and ripening, and changes in ABA-related pathways and gene expression have been extensively investigated during strawberry ripening [23,24]. Specific strawberry *β*-glucosidases have also been associated with fruit development and ripening. In *Fragaria × ananassa*, FaBG1 has been characterized in relation to strawberry fruit ripening [25], while FaBG3 has been implicated in fruit ripening and the response to *Botrytis cinerea* infection [26].

Wild strawberry (*F. vesca*), a diploid woodland species, is widely used as a genetic reference model for studying ripening and hormone regulation within the genus. Its relatively simple genome facilitates gene identification, functional annotation, and pathway characterization. However, the present study focuses on the cultivated strawberry (*F. × ananassa*), an allo-octoploid species of major agricultural importance, in which metabolic processes directly determine fruit quality traits such as flavor, aroma, and nutritional composition. Although the genetic complexity of *F. × ananassa* poses challenges for gene characterization, comparative analysis with *F. vesca* provides a valuable framework for identifying homologous genes and guiding functional investigations in the cultivated species [27].

Although *β*-glucosidases have been characterized in several plant species, the substrate specificity and catalytic properties of individual *β*GLs in cultivated strawberry (*F. × ananassa*) remain insufficiently characterized. This is particularly relevant for flavonoid glycosides because glycosylation can alter the solubility, stability, compartmentalization, availability, and biological activity of phenylpropanoid and flavonoid compounds [15]. Consequently, both glycosylation and deglycosylation represent important processes controlling the chemical form and availability of these secondary metabolites in plants [15].

Considerable attention has been given to the formation of glycosylated metabolites in strawberry. Several UDP-dependent glycosyltransferases (UGTs) have been functionally characterized in *F. × ananassa*, demonstrating substantial differences in substrate specificity and the ability of individual enzymes to glycosylate flavonoids and other secondary metabolites [17]. Notably, strawberry UGTs have been shown to produce different flavonoid glucosides, including 3- and 7-*O*-glucosides, illustrating the diversity of glycosylation reactions occurring within strawberry secondary metabolism [17]. However, compared with the characterization of enzymes responsible for glycoside formation, considerably less is known about the substrate preferences and catalytic properties of individual strawberry *β*-glucosidases that may catalyze the reverse process. In particular, the extent to which individual strawberry *β*GLs discriminate between structurally related flavonoid glucosides and between different positions of *O*-glycosylation remains poorly understood.

In the present study, transcriptomic information from *F × ananassa* was used to identify *β*GL candidates expressed in strawberry tissues, leading to the selection of *FxaC_10g48920.t1* as a candidate for biochemical characterization. The encoded protein, designated Fra-*β*GL, was heterologously expressed and purified to investigate its catalytic properties and substrate selectivity. Particular attention was given to naturally occurring flavonoid glucosides differing in both aglycone structure and glucosylation position. A commercial *β*-glucosidase from *Prunus dulcis* (Pru-*β*GL) was included as a reference enzyme to provide a comparative framework for evaluating the catalytic characteristics of Fra-*β*GL.

Therefore, the present study aimed to biochemically characterize Fra-*β*GL, determine its substrate specificity toward selected naturally occurring flavonoid and phenolic glycosides, and evaluate its kinetic properties toward accepted natural substrates and the synthetic substrate *p*NP-Glc. Particular emphasis was placed on comparing structurally related flavonoid 3- and 7-*O*-glucosides to examine whether the position of glycosylation influences substrate recognition. In addition, molecular docking was used to explore structural features that may contribute to the experimentally observed substrate selectivity. Together, these analyses contribute to the biochemical characterization of Fra-*β*GL and provide a basis for further investigation of its potential biological relevance in strawberry.

## 2. Materials and Methods

### 2.1. Chemicals

All chemicals that were utilized for the preparation of buffers and standard solutions were of analytical grade. The substrates for enzyme activity testing were purchased from various manufacturers: arbutin (≥98%) and phloridzin dihydrate (≥99%) (Merck, Darmstadt, Germany); cyanidin 3-*O*-glucoside (≥97%), luteolin 7-*O*-glucoside (≥99%), pelargonidin 3-*O*-glucoside (≥95%) and kaempferol 3-*O*-glucoside (≥99%) (Carl Roth GmbH, Karlsruhe, Germany); kaempferol 7-*O*-glucoside (≥99%) (Ambeed, Inc., Buffalo Grove, IL, USA); *p*NP-Glc (≥98%) (BLD Pharmatech GmbH, Kaiserslautern, Germany); quercetin 3-*O*-glucoside (≥95%) (Activate Scientific GmbH, Prien am Chiemsee, Germany); and quercetin 7-*O*-glucoside (≥98%) (Cayman Chemical, Ann Arbor, MI, USA).

### 2.2. β-Glucosidase Genes Selection and Transcript Quantification

*β*GL gene sequences from *F. vesca* were retrieved from the NCBI nucleotide database and partial or incomplete ones were discarded. In order to obtain the orthologous genes in *F. × ananassa*, a TBLASTN search was carried out, with the set of sequences retrieved from *F. vesca* against the genome transcripts v1.0.a2 database for the cultivar Camarosa available on https://www.rosaceae.org/ (accessed on 8 August 2023). The hit transcripts were sorted to remove putative isoforms and quantified at tissue level for the receptacle ripe stage with several transcriptomic databases (ERR1817375, ERR1817376, ERR1817377, ERR1817378, ERR1817379, ERR1817380, SRR5826137, SRR5826138, SRR10076577, SRR10076581, SRR10076582, SRR10076583, SRR10076585, SRR10076586) indexed with k-mer length of 31 using the Salmon quantification tool (v1.9) with default settings [28]. Instead of selecting the transcript solely with the highest expression across all the tissues, we focused on the set of top-expressed *β*GL candidates in ripe receptacle tissue. Further tissue quantification was carried out in the achene (green, white, turning, ripe stages), receptacle (green, white, turning, ripe stages), leaf, root and petals. A list of the Sequence Read Archives (SRAs) used is available in the Supplementary Materials. Deeploc 2.0 [29] was used to assess subcellular localization.

### 2.3. Gene Cloning, Protein Expression and Purification

The coding sequence from *FxaC_10g48920.t1* was obtained from the rosaceae.org website and translated to the amino acid counterpart via the Expasy online tool (https://web.expasy.org/translate/, accessed on 8 August 2023). SignalP 6.0 (https://services.healthtech.dtu.dk/services/SignalP-6.0/, accessed on 10 August 2023) was used to scan the sequence for potential signal peptides, and one was discovered with a predicted cleavage position between the amino acids 24 and 25. Primers were designed based on this information as follows (Fa_BetaGluc_forward: 5′-ACCTCCCCTAGCCATTACGATG-3′ and Fa_BetaGluc_reverse: 5′-GTTTCCAAGAAAATTCTTGAACCAGTGTGC-3′). mRNA was isolated from *F. × ananassa* whole fruit tissue consisting of flesh and achenes without the calyx. Samples were frozen in liquid nitrogen, finely ground using an IKA Basic blender, and mRNA was purified with a μMACS mRNA isolation kit (Miltenyi Biotec, Bergisch Gladbach, Germany). First-strand cDNA was synthesized using RevertAid™ Reverse Transcriptase (Thermo Fisher Scientific, Inc., Waltham, MA, USA). cDNA derived from receptacles at the ripe stage was used as the template for gene amplification with Q5 High-Fidelity DNA Polymerase (New England Biolabs, Ipswich, MA, USA). The PCR product was resolved on a 1% agarose gel, excised, and purified prior to cloning into the pTrcHis2-TOPO expression vector (Thermo Fisher Scientific, Inc., Waltham, MA, USA) according to the manufacturer’s instructions. This vector encodes a C-terminal 6x His tag to enable purification by affinity chromatography. *E. coli* Stellar™ competent cells (Takara Bio Inc., Shiga, Japan) were used for transformation, while *E. coli* BL21(DE3) competent cells were employed for recombinant protein expression.

Protein expression and purification were carried out using the same methodology described by Zavarise et al. (2025) with minor modifications [30]. In brief, a single colony was used to inoculate 20 mL of LB medium and cultured overnight at 37 °C. The following morning, the entire overnight culture was used to inoculate 1 L of TB medium. Protein expression was induced with 1 mM IPTG at OD_600_ = 0.6–0.8. The Temperature in the incubator was decreased to 18 °C and expression was carried out overnight. Cell lysis was carried out with a cell disruptor Q125 (QSonica, Newtown, CT, USA) and purification was carried out via immobilized metal affinity chromatography (IMAC) with an Äkta Purifier 10 (GE Healthcare, Uppsala, Sweden).

Protein integrity and purity were assessed via SDS-PAGE and Western blotting. SDS-PAGE samples were mixed with 4x Laemmli buffer containing 2-mercaptoethanol and incubated for 10 min at 95 °C. A 12% acrylamide gel was used for separation in combination with a BioRad Tetra system (BioRad Laboratories, Hercules, CA, USA) and Color Prestained Protein Standard, Broad Range (New England Biolabs, Ipswich, MA, USA) was used as a size standard. Protein bands were then transferred to a PVDF membrane with a Trans-Blot Turbo Transfer System (BioRad Laboratories, Hercules, CA, USA). A phosphate-buffered saline (PBS) solution containing 2% (*w*/*v*) bovine serum albumin was used to block unspecific interactions for 1.5 h and washed twice for 10 min in PBS buffer with 0.4% (*v*/*v*) Tween-20 at the end of incubation. AntiHis antibody conjugated to alkaline phosphatase was incubated in 1:5000 dilution for 1.5 h and washed twice briefly with 10 mL of PBS buffer with 0.4% (*v*/*v*) Tween-20. Color development was achieved with 5-bromo-4-chloro-3-indolyl-phosphate and nitro blue tetrazolium in alkaline phosphatase buffer.

### 2.4. Determination of Enzyme Concentration

The concentration of the recombinantly expressed and purified Fra-*β*GL was determined by the Lowry method, using bovine serum albumin as a standard [31]. Absorbance at 750 nm was measured, and concentrations were interpolated from a standard calibration curve. For commercially sourced Pru-*β*GL, the protein concentration was estimated spectrophotometrically from absorbance at 280 nm using the manufacturer-supplied specific absorbance coefficient *E_1%_* = 7.06. Measurements were corrected for dilution and path length, and the protein concentration was subsequently converted to molar concentration using the molecular mass of one enzyme subunit. The different quantification approaches reflect the different origins of the glucosidases and available characterization of the two enzyme preparations.

### 2.5. Enzyme Assays

The enzymatic activity was assessed using two distinct analytical methods, selected according to the properties of the respective substrates. Substrates that did not produce any chromogenic or detectable absorbance change during enzymatic conversion were analyzed using high-performance liquid chromatography (HPLC), which allowed accurate separation and quantification of substrates and products. For substrates that generated measurable absorbance changes during the reaction, spectrophotometric assays were performed. Eight naturally occurring compounds were analyzed by HPLC, whereas the synthetic substrate *p*NP-Glc was analyzed spectrophotometrically.

The synthetic substrate *p*NP-Glc was assayed using a Shimadzu UV-1800 spectrophotometer equipped with a CPS-controller unit (Shimadzu, Kyoto, Japan). Substrate concentrations ranging from 1 to 25 mM were tested. Pru-*β*GL and Fra-*β*GL were used at final concentrations of 74 and 90 nM, respectively. Reactions were performed in 100 mM citrate buffer (pH 4.5) at 30 °C for 10 min. These conditions were selected as standardized conditions for comparison of Fra-*β*GL and Pru-*β*GL and are consistent with conditions commonly used for the biochemical characterization of plant *β*-glucosidases [32,33,34]. The final volume per cuvette was 1.0 mL, and all measurements were performed in triplicate.

The naturally occurring substrates were assayed at concentrations ranging from 40 µM to 1 mM, with the maximum substrate concentration adjusted according to the solubility of each compound. Reactions were performed in 100 mM citrate buffer (pH 4.5) at 30 °C for 1 h. Pru-*β*GL and Fra-*β*GL were used at 370 nM and 73 nM, respectively. Reactions were terminated by heating at 90 °C for 5 min.

HPLC analyses for enzyme assays with flavonoid glycosides were performed on a Thermo Scientific Dionex UltiMate^®^ 3000 RSLC system (Thermo Fisher Scientific Inc., Waltham, MA, USA) equipped with an RS pump (HPG-3200RS binary), autosampler, column compartment, and diode array detector, with data processed using Chromeleon™ software (v7.1.3.2425). Separation was achieved on an Acclaim™ RSLC 120 C18 column (2.2 µm, 120 Å, 2.1 × 150 mm) with an Acclaim™ 120 C18 guard column (2.1 × 10 mm, 5 µm, 120 Å) at 25 °C. The mobile phase consisted of (A) ultrapure water and (B) acetonitrile, both containing 0.1% formic acid, delivered at 0.2 mL/min with the following gradient: 7.5% B (0–22 min to 53% B; 22–27 min to 95% B; held to 35 min; returned to 7.5% B by 35.5 min; re-equilibrated to 38 min). Injection volumes were 8 µL. Detection wavelengths were 360 nm for kaempferol, quercetin, and their glycosides; 340 nm for luteolin and its glycosides; and 290 nm for phloretin and phloridzin, with spectra recorded from 190 to 800 nm. Acetonitrile (HiPerSolv CHROMANORM^®^, VWR Chemicals, Radnor, PA, USA) and LC-MS–grade formic acid (ROTIPURAN^®^, Carl Roth GmbH. KG, Karlsruhe, Germany) were used with ultrapure water from a Direct-Q^®^ 3UV system (Merck, Darmstadt, Germany).

Enzyme assays with pelargonidin and cyanidin glycosides were analyzed on the same HPLC system and software as above, using the same column setup at 25 °C. The mobile phase comprised (A) 10% formic acid in water and (B) 22.5% methanol, 22.5% acetonitrile, and 10% formic acid in water, run at 0.2 mL/min with a gradient from 9% to 90% B over 30 min, held to 40 min. Injection volumes were 8 µL, with detection at 520 nm and spectral acquisition from 190 to 800 nm.

Substrate conversion was evaluated by HPLC based on changes in substrate peak area and the appearance of the corresponding aglycone product. Product identity was confirmed by comparison of retention times with authentic aglycone standards analyzed under the same chromatographic conditions. Substrates were classified as showing no detectable activity when no decrease in substrate peak area and no corresponding aglycone product peak were detectable relative to the control under the assay conditions. As a formal numerical limit of detection was not established, negative results are reported as “no detectable activity” rather than absence of activity. A panel of flavonoid and phenolic glycosides was tested to identify potential substrates (Table 1). Both enzymes displayed activity toward kaempferol 7-*O*-glucoside, luteolin 7-*O*-glucoside, quercetin 7-*O*-glucoside, and the synthetic substrate *p*NP-Glc. No measurable activity was detected for arbutin, cyanidin 3-*O*-glucoside, pelargonidin 3-*O*-glucoside, phloridzin, or any tested 3-*O*-glucosides of kaempferol and quercetin.

### 2.6. Kinetic Studies

Kinetic analyses were performed for both the Fra-*β*GL and Pru-*β*GL preparations using three naturally occurring substrates, quercetin 7-*O*-glucoside, kaempferol 7-*O*-glucoside, and luteolin 7-*O*-glucoside, and the synthetic substrate, *p*NP-Glc. Kinetic parameters including *V*_max_, *K*_cat_, *K*_m,_ and *K*_cat_/*K*_m_ were calculated using OriginPro software (Version 2023; OriginLab Corporation, Northampton, MA, USA). *V*_max_ values are available in Supplementary Table S1.

### 2.7. Effect of pH and Temperature on β-Glucosidase Activity and Thermal Stability

The effects of pH and temperature on the activities of Fra-*β*GL and Pru-*β*GL were evaluated using *p*NP-Glc as a substrate. Fra-*β*GL and Pru-*β*GL were used at a final protein concentration of 0.1 µg/mL, corresponding to approximately 1.82 and 1.48 nM, respectively, based on molecular masses of 55 and 67.5 kDa.

The effect of pH on *β*-glucosidase activity was examined over the range pH 3.0–7.5. 100 mM citric acid buffer (pH 3–6.2) was used for the acidic pH range, whereas 100 mM sodium phosphate buffer (pH 5.8–8) was used for the higher pH range. Enzyme activity at each pH was determined under otherwise identical assay conditions and expressed as relative activity. For each enzyme, the highest activity measured within the tested pH range was defined as 100%, and activity at the remaining pH values was expressed relative to this maximum.

The effect of reaction temperature on *β*-glucosidase activity was evaluated over the range 20–80 °C. Enzymatic reactions were performed at the respective temperatures, using *p*NP-Glc as a substrate under otherwise identical assay conditions. For each enzyme, the highest activity measured within the tested temperature range was defined as 100%, and activity at the other temperatures was expressed as relative activity.

Thermal stability was evaluated using a fixed-time residual-activity approach similar to that described for *β*-glucosidases [35]. The evaluation was done by incubating Fra-*β*GL and Pru-*β*GL at 30, 40, 50, 60, and 70 °C for 30 min in the absence of substrate. Following thermal treatment, residual *β*-glucosidase activity was determined using *p*NP-Glc under the standard assay conditions. Residual activity was expressed relative to the corresponding non-heated control, which was defined as 100%. All measurements were performed in triplicate.

### 2.8. Ligand Docking

A 3D model of the protein was created with the use of Alphafold 2.0 [36]. Ligand docking with the enzyme was performed using the free software AutoDock Vina (v1.2.7) [37]. Docking calculations were carried out with a search box size of 20 Å, an exhaustiveness parameter of 64, and a maximum of 20 output poses.

## 3. Results and Discussion

### 3.1. Candidate Gene Selection and Expression Study

A total of 31 *β*GL protein sequences were retrieved from the NCBI database for *F. vesca*, and InterProScan analysis [38] assigned all sequences to glycoside hydrolase family 1 (GH1). A TBLASTN search against the *F. × ananassa* cultivar Camarosa genome resulted in 27 hits in total, with 25 of these with a highly homologous hit (>90% protein ID) in *F. vesca*. All transcripts were kept to not exclude any potential candidate when looking at the level of expression in the fruit at the ripe stage.

**Figure 1:**
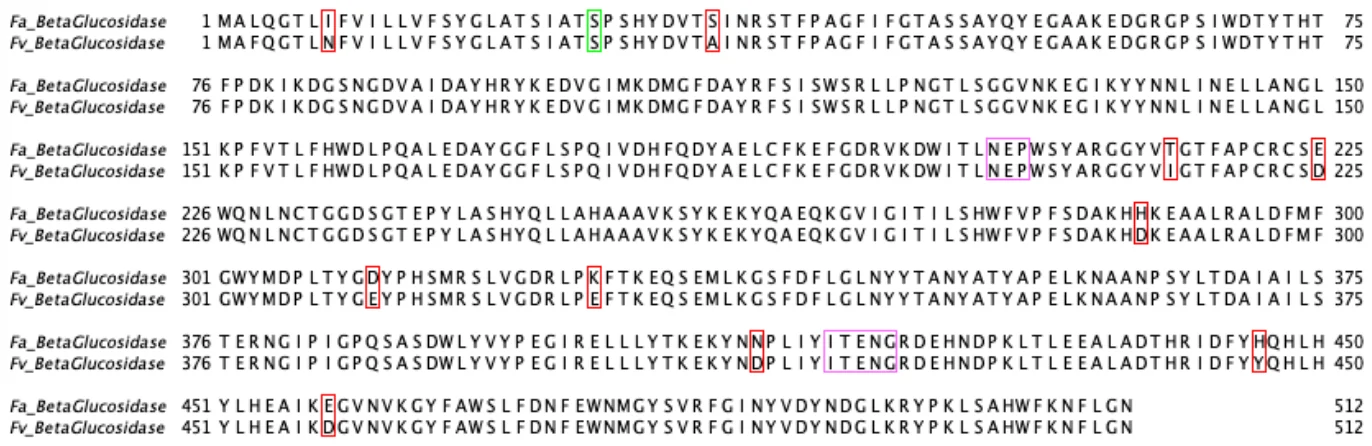
Sequence alignment for *β*GL 12-like from *Fragaria × ananassa* (Fa) and *Fragaria vesca* (Fv). Red boxes highlight amino acid differences; green boxes highlight the cleavage point in vivo for the signal peptide; pink boxes contain the conserved motif for catalysis in the plant GH1 family.

The transcript quantification was carried out with the Salmon quantification tool (v1.9) (https://combine-lab.github.io/salmon/, accessed on 8 August 2023) [28] on 14 different SRA studies and showed no expression for 11 different genes while the remaining genes showed highly variable expression levels. The transcript *FxaC_10g48920.t1* was among the more highly expressed transcripts and was identified as the putative ortholog to the *β*GL 12-like XP_011461905 from *F. vesca*. Figure 1 shows the alignment between the two different sequences made in Jalview (v2.11.4.1).

Non-conserved amino acids are highlighted by red boxes and are located on the surface of the protein, uggesting that conserved structural and catalytic features may be retained between the two proteins. In the pink boxes, the motifs NEP and ITENG are highlighted and are known to be highly conserved in the GH1 family for catalysis [39], with the first glutamic acid acting as an acid/base catalyst while the second one acts as the nucleophile. Amino acid no. 25 (green box) was flagged by SignalP 6.0 as the cleavage point in vivo for the signal peptide. To gain more insight into the possible localization of the enzyme, the sequence was analyzed with the online tool DeepLoc 2.0, and it was assessed with a predicted probability of 0.63 for vacuolar localization. This aligns well with what was previously described in the literature where *β*GLs are often targeted to the vacuole for stress resistance, hormone regulation and glycoside metabolism [40,41,42].

**Figure 2:**
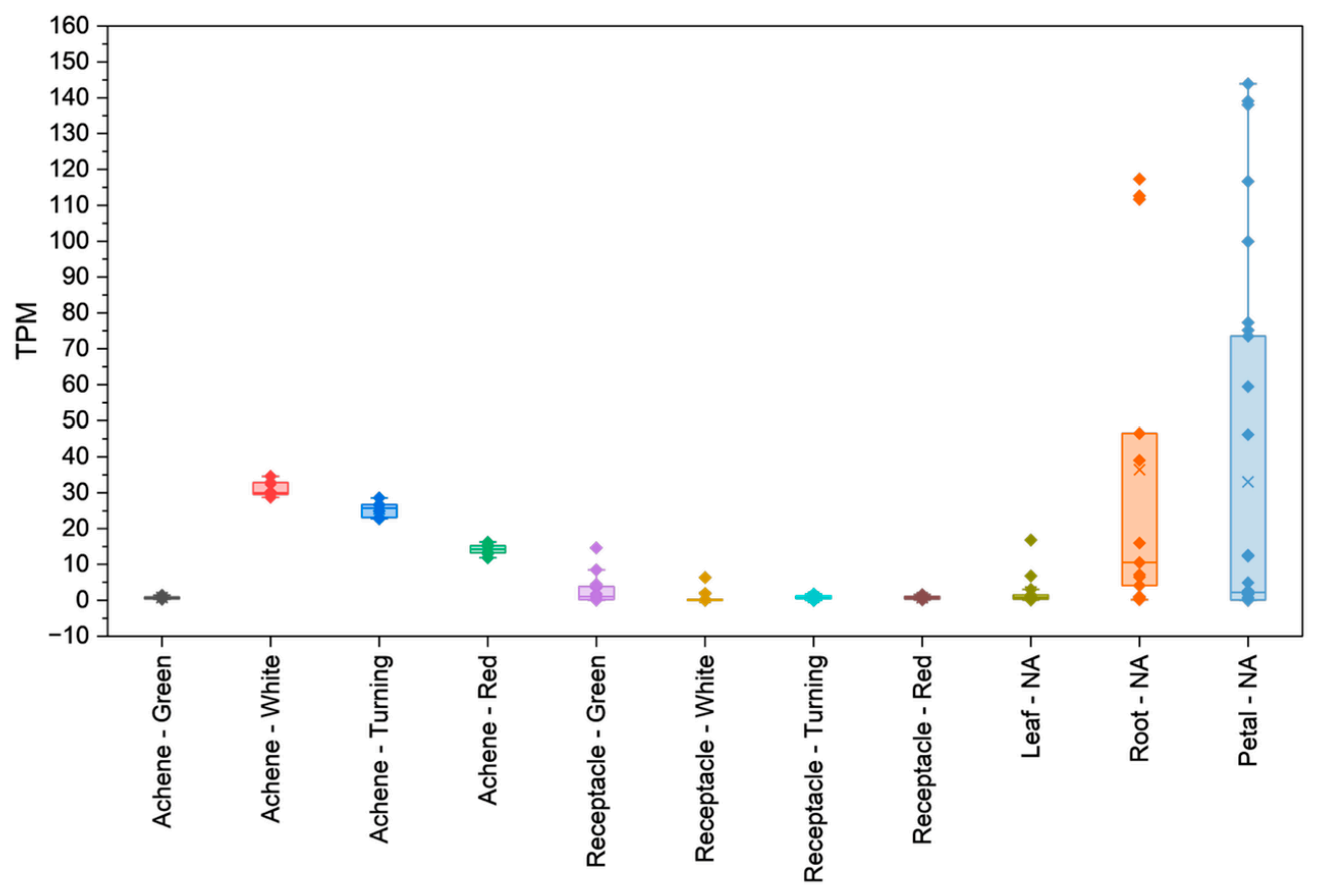
Box plot for transcriptomic abundance of the candidate *β*GL at different tissue levels with median value and standard deviation. *Y*-axis: TPM (transcripts per million). *X*-axis: Different tissues grouped by ripening stage (green, white, turning, red); for leaf, root and petal, no stage is specified (NA).

To build on the initial expression analysis, a more detailed examination of the transcript’s expression pattern across different tissues was conducted to better characterize the enzyme’s expression in the plant. In total, 139 SRAs from the NCBI databases were used and are available in the Supplementary File. The tissues considered were achene and receptacle at the green, white, turning and red ripening stage, leaf, root and petals. The plot in Figure 2 shows the results as a grouped box with a median value and standard deviation. The transcript showed relatively high abundance in achenes, roots, and petals, even though the dispersion for the latter is quite severe and might be attributed to combining data from different varieties in the SRA studies. The higher TPM observed in roots (around 110) is derived from RNA-seq analysis conducted by Sanchez et al. [43] but seems almost to be an outlier when compared to the other SRAs available that display values ranging from slightly above 0 to a maximum of 50. Values were more consistent when looking at all the ripening stages in the achene and receptacle. In the achene, a downward trend can be observed from white to ripe stage, while in the receptacle it stays mostly consistent at all stages with a little bit of dispersion during initial growth. In leaves, the transcript’s abundance is at a similarly low level as during the green stage in the receptacle, with values oscillating between 0 and 20.

While the result of the study highlights the gene *FxaC_10g48920.t1* as a promising candidate, it is necessary to state that re-quantification of publicly available SRA datasets, even with a linearized pipeline, has inherent biological and technical limitations due to cross-study heterogeneity and batch effects [44,45,46]. This is due to the observed transcript abundance being influenced by cultivars, growth conditions, and library preparation, along with non-biological batch effects [45,47]. Furthermore, the gene expression pattern can vary considerably across cultivars, given the polyploid complexity of the strawberry genome [48,49]. Independent expression analysis using RT-qPCR with standardized biological material would be valuable to determine whether the tissue-specific expression patterns observed in the publicly available RNA-seq datasets are reproducible under controlled conditions.

### 3.2. Cloning, Expression and Purification of β-Glucosidase

**Figure 3:**
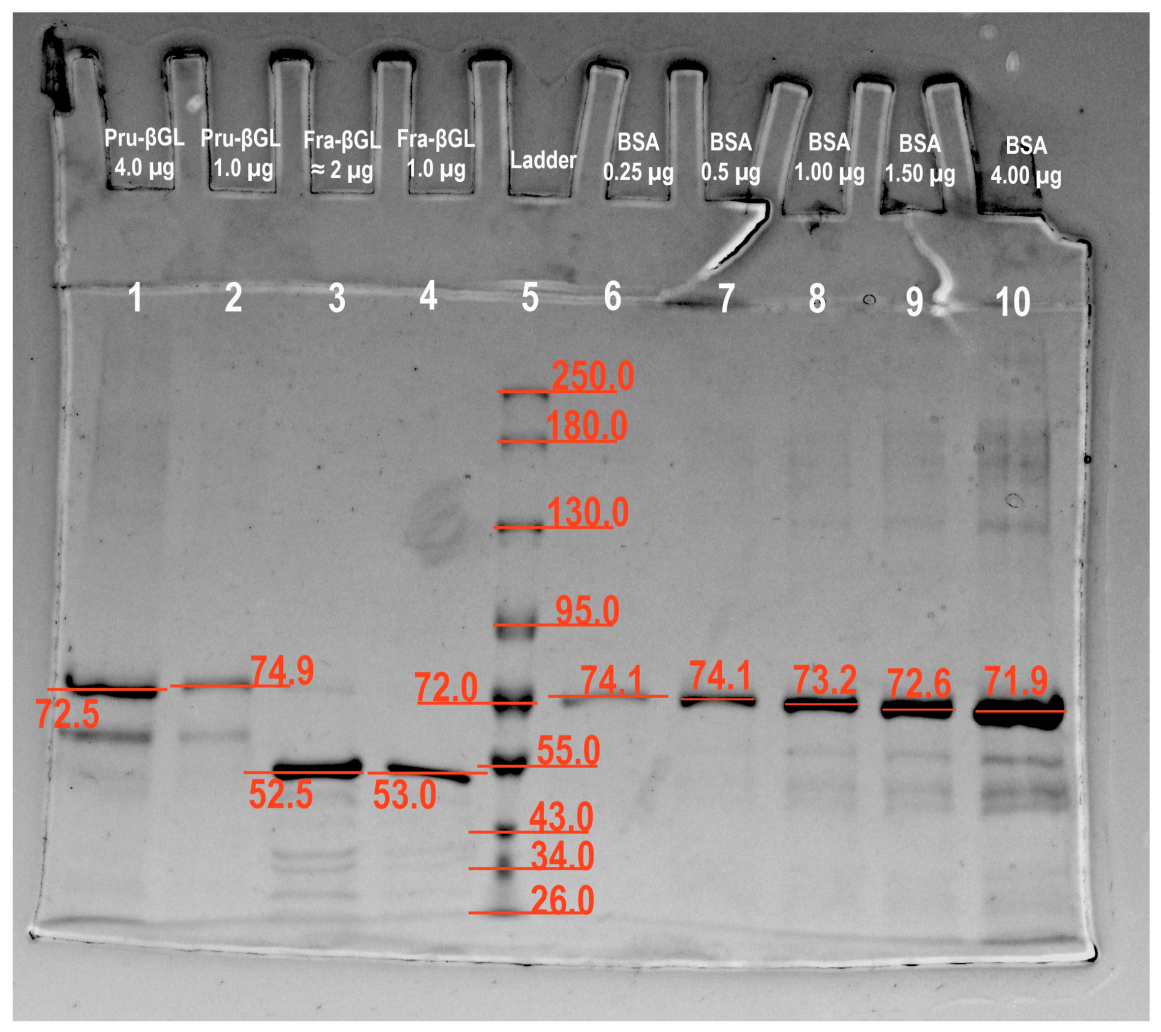
SDS-PAGE analysis of Fra-*β*GL and Pru-*β*GL preparations. Molecular-mass markers are indicated in kDa.

The Fra-*β*GL cDNA was successfully cloned into the pTrcHis2-TOPO vector and expressed in *E. coli* in soluble form. Following IMAC purification, the recombinant Fra-*β*GL yield was approximately 23 mg per liter of bacterial culture. SDS-PAGE analysis showed a predominant protein band with an apparent molecular mass of approximately 53–55 kDa, consistent with the predicted molecular mass of recombinant Fra-*β*GL (~55 kDa), although minor additional protein bands remained after purification (Figure 3).

### 3.3. Enzyme Activity and Kinetics

Enzyme activity screening was performed using recombinant Fra-*β*GL and commercial Pru-*β*GL. Different enzyme concentrations were used for the two preparations to obtain measurable substrate conversion under the respective assay conditions, as described in Section 2.5.

Kinetic parameters for the substrates accepted by the *β*GLs are summarized in Table 2. For the naturally occurring substrates kaempferol 7-*O*-glucoside and luteolin 7-*O*-glucoside, both enzymes exhibited comparable substrate affinities, with *K*_m_ values ranging from 0.49 to 0.89 mM. Fra-*β*GL showed higher catalytic efficiency toward both substrates. For kaempferol 7-*O*-glucoside, Fra-*β*GL exhibited a *K*_cat_/*K*_m_ of 1.60 mM^−1^·s^−1^ compared with 0.48 mM^−1^·s^−1^ for Pru-*β*GL. Similarly, for luteolin 7-*O*-glucoside, Fra-*β*GL exhibited a catalytic efficiency of 2.09 mM^−1^·s^−1^ compared with 0.47 mM^−1^·s^−1^ for Pru-*β*GL. The synthetic substrate *p*NP-Glc was hydrolyzed by both enzymes, with similar *K*_m_ values of approximately 5.9 mM. However, Fra-*β*GL displayed a markedly higher turnover rate (*K*_cat_ = 60.33 ± 1.17 s^−1^) than Pru-*β*GL (*K*_cat_ = 0.90 ± 0.03 s^−1^). Consequently, the catalytic efficiency of Fra-*β*GL toward *p*NP-Glc was 10.18 ± 0.32 mM^−1^·s^−1^, compared with 0.15 ± 0.01 mM^−1^·s^−1^ for Pru-*β*GL. Thus, *p*NP-Glc showed the highest turnover rate and catalytic efficiency among the substrates tested with Fra-*β*GL. In contrast to *p*NP-Glc, quercetin 7-*O*-glucoside exhibited substantially lower *K*_m_ values for both enzymes (0.05–0.06 mM), indicating higher apparent affinity for this substrate. Fra-*β*GL exhibited a *K*_cat_ of 0.23 ± 0.02 s^−1^ and a catalytic efficiency of 4.14 ± 0.47 mM^−1^·s^−1^, whereas Pru-*β*GL exhibited a *K*_cat_ of 0.043 ± 0.003 s^−1^ and a catalytic efficiency of 0.824 ± 0.071 mM^−1^·s^−1^.

Overall, Fra-*β*GL exhibited higher catalytic efficiency than Pru-*β*GL toward all four substrates, although the magnitude of the difference was substrate-dependent. The greatest difference was observed for the synthetic substrate *p*NP-Glc, for which Fra-*β*GL was approximately 68-fold more efficient. Among the naturally occurring flavonoid glucosides, the greatest catalytic efficiency of Fra-*β*GL was observed toward quercetin 7-*O*-glucoside, followed by luteolin 7-*O*-glucoside and kaempferol 7-*O*-glucoside.

### 3.4. Effect of pH and Temperature on β-Glucosidase Activity and Thermal Stability

**Figure 4:**
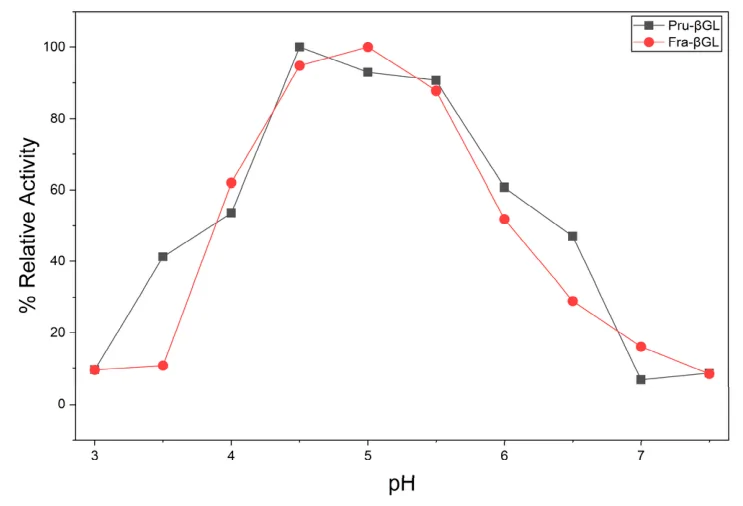
Effect of pH on the relative activity of Fra-*β*GL and Pru-*β*GL at 30 °C. Numerical values are provided in Supplementary Table S2.

The effect of pH on the activities of Fra-*β*GL and Pru-*β*GL was evaluated using *p*NP-Glc as substrate. Both enzymes exhibited their highest activities under acidic conditions, although their pH-dependent activity profiles differed. Fra-*β*GL showed its highest measured activity at pH 5.0, which was defined as 100% relative activity. High activity was also retained at pH 4.5, corresponding to approximately 95% of the maximum. In contrast, Pru-*β*GL exhibited its highest measured activity at pH 4.5. For both enzymes, activity decreased progressively as the pH increased and was markedly reduced toward neutral pH (Figure 4). The preference of both enzymes for acidic conditions is consistent with previous reports for plant *β*-glucosidases. A soybean *β*-glucosidase hydrolyzing the isoflavone glucosides daidzin and genistin showed an optimum at pH 4.5 [32], while acidic assay conditions have also been reported for *β*-glucosidase from guar [33].

**Figure 5:**
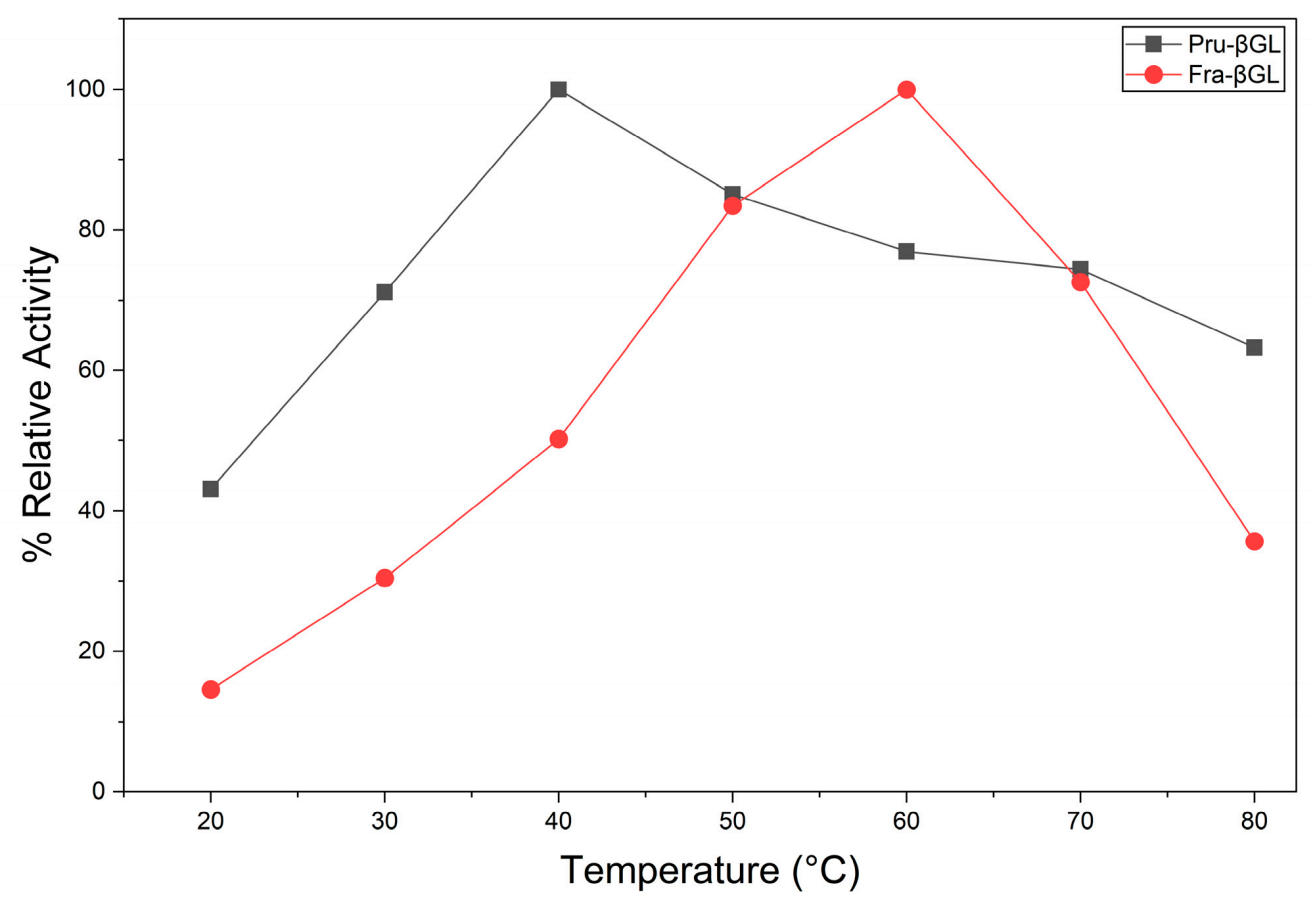
Effect of reaction temperature on the relative activity of Fra-*β*GL and Pru-*β*GL at pH 4.5. Numerical values are provided in Supplementary Table S3.

The two enzymes also displayed different temperature-dependent activity profiles. Fra-*β*GL activity increased with increasing reaction temperature and reached its highest measured activity at 60 °C, which was defined as 100%. Activity decreased at higher temperatures, with approximately 73% of the maximum retained at 70 °C and approximately 35% at 80 °C. Pru-*β*GL exhibited its highest measured activity at 40 °C. Above 40 °C, its relative activity decreased gradually, although measurable activity was retained throughout the tested temperature range (Figure 5). The temperature-dependent activity profiles of the two enzymes differed substantially. Although 30 °C was used as the standardized temperature for kinetic characterization, this temperature was selected as a common comparative assay condition rather than as the activity optimum of either enzyme. The use of 30 °C is consistent with conditions previously employed for biochemical characterization of plant *β*-glucosidases, including recombinant rice *β*-glucosidase [34].

**Figure 6:**
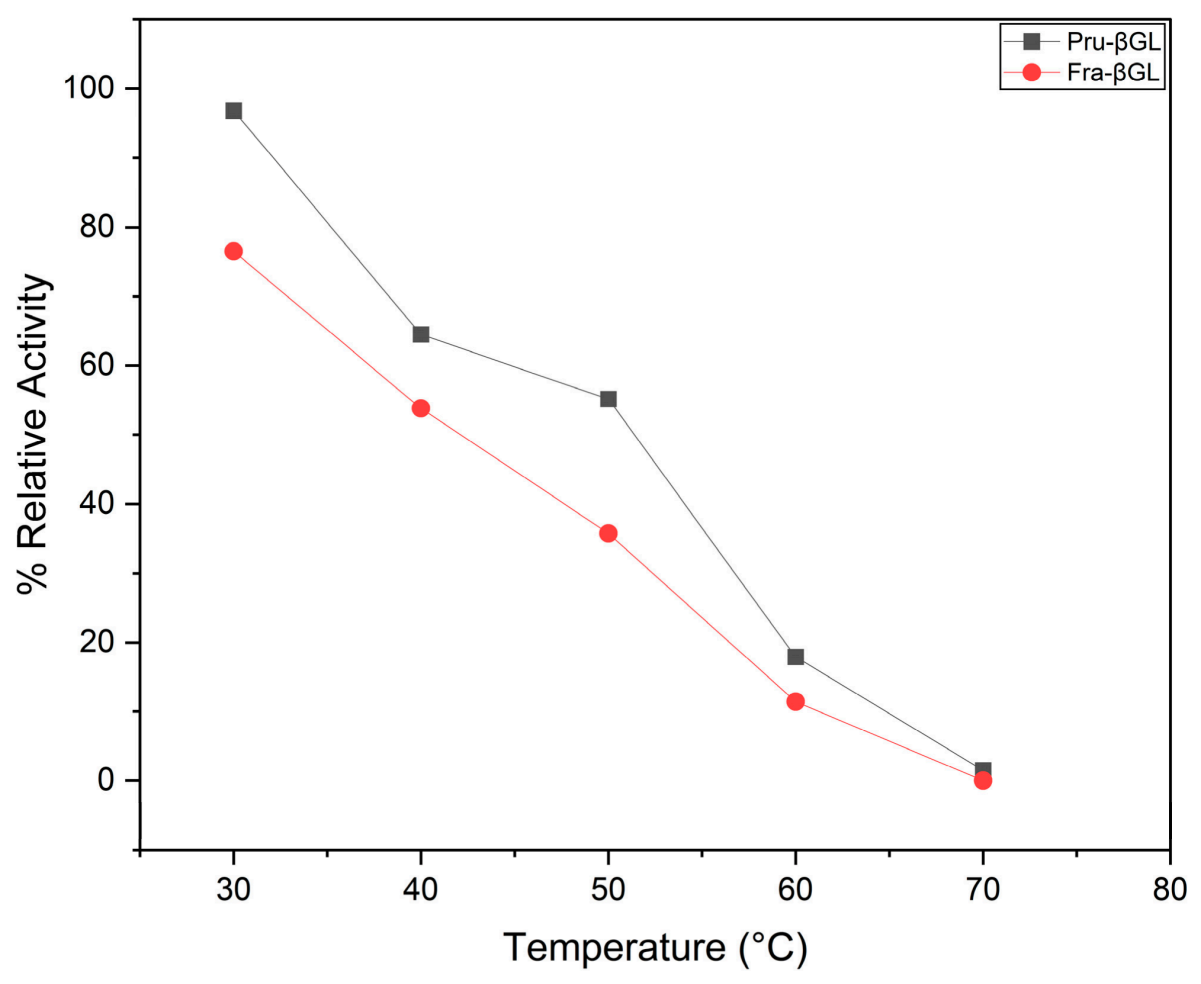
Thermal stability of Fra-*β*GL and Pru-*β*GL at pH 4.5 following 30 min thermal treatment at the indicated temperatures. Numerical values are provided in Supplementary Table S4.

Thermal stability was evaluated separately by measuring fixed-time residual activity following incubation for 30 min at different temperatures similar to those previously employed for the characterization of *β*-glucosidases [35]. Both enzymes showed progressive loss of activity with increasing pre-incubation temperature (Figure 6). Pru-*β*GL retained 96.8% of its initial activity after incubation at 30 °C, whereas Fra-*β*GL retained 76.5%. At 40 °C, residual activities were 64.5% and 53.8% for Pru-*β*GL and Fra-*β*GL, respectively, and at 50 °C they decreased to 55.2% and 35.8%. Following incubation at 60 °C, Pru-*β*GL retained 17.9% residual activity and Fra-*β*GL retained 11.4%. At 70 °C, Pru-*β*GL retained only 1.5% activity, whereas no detectable residual activity was observed for Fra-*β*GL (Figure 6). Overall, Pru-*β*GL exhibited greater thermal stability than Fra-*β*GL under the tested conditions. Importantly, the temperature supporting the highest short-term catalytic activity did not directly correspond to thermal stability: Fra-*β*GL showed its highest measured catalytic activity at 60 °C but was substantially inactivated following prolonged incubation at this temperature.

### 3.5. Docking of Quercetin 3-O-Glucoside and Quercetin 7-O-Glucoside

Following the results from the kinetic analysis, we decided to investigate the reasons that could explain the inability of Fra-*β*GL to convert 3-*O*-substituted substrates into their aglycone form.

**Figure 7:**
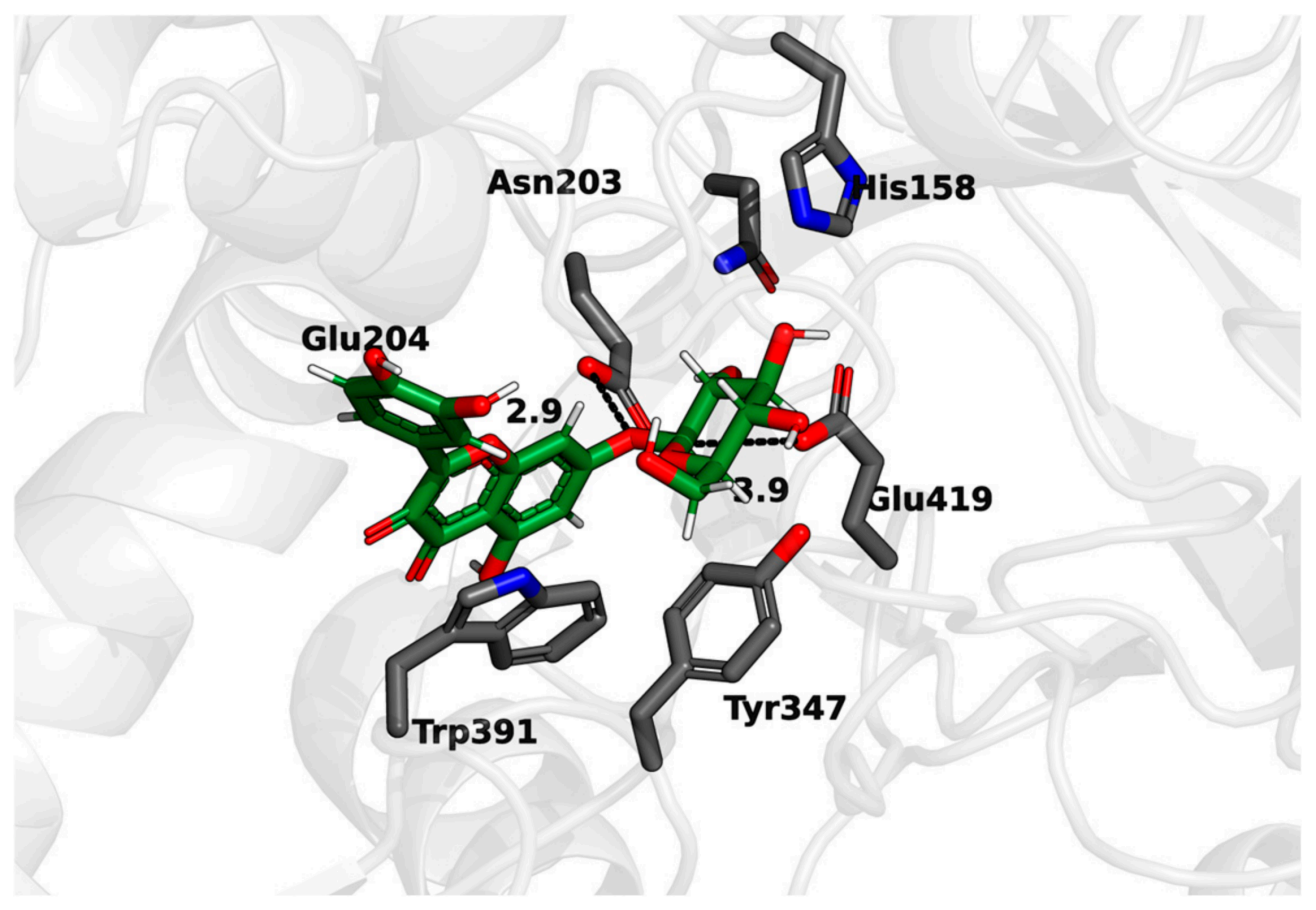
Molecular visualization of Fra-*β*GL with the docked ligand quercetin 7-*O*-glucoside. The enzyme is light gray and the substrate is green. Distances between catalytic oxygens and the ligand are displayed as dashed black lines. Key interacting residues are in dark gray.

**Figure 8:**
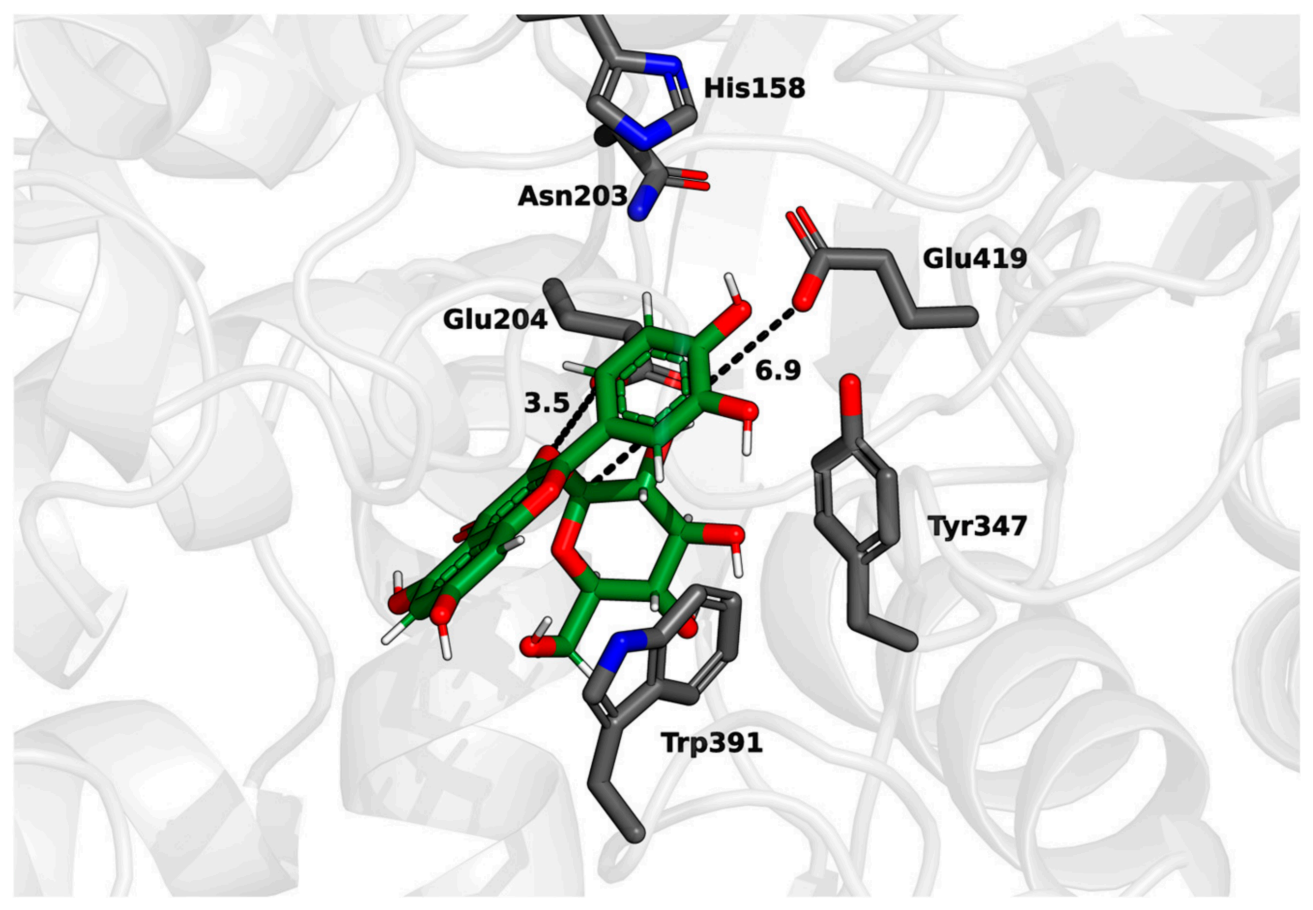
Molecular visualization of *β*GL with the docked substrate quercetin 3-*O*-glucoside. The enzyme is light gray and the substrate is green. Distances between catalytic oxygens and the ligand are displayed as dashed black lines. Key interacting residues are in dark gray.

The protein sequence was used with Alphafold 2.0 [36] to model the 3D structure of the enzyme. The receptor and the substrates were prepared for docking with AutoDock Vina by removing water molecules, adding polar hydrogens and Gasteiger charges. The box search was set to 20, exhaustiveness of poses to 64 and number of poses to 20. Results were inspected using PyMOL (v2.6.2) [50] and poses with sugar orientation towards the exit of the active site were discarded. The best pose for each substrate was selected and they can be seen in Figure 7 and Figure 8.

*β*GLs of the GH1 family are known to follow a retaining catalytic mechanism, in which one glutamate residue acts as a nucleophile, attacking the anomeric carbon of the sugar, while the second glutamate functions as the acid/base, protonating the glycosidic oxygen [22,51]. In Table 3, the values calculated by AutoDock Vina for each pose are reported:

AutDock Vina predicted energies for each pose were strong across all models (−10.76 kcal/mol to −7.064 kcal/mol), except for pose 20. While pose 1 yielded the lowest binding affinity (−10.76 kcal/mol), pose 5 (−9.911 kcal/mol) maintained tight convergence (RMSD l.b = 1.015 Å) while placing the ligand within functional catalytic distance. The nucleophilic residue (Glu419) and the anomeric carbon are spaced approximately 3.9 Å apart, while the acid/base (Glu204) is 2.9 Å from the glycosidic oxygen of the A ring, which lies within the limits of what is typically considered catalytically viable.

The ligand also appears to be stabilized by π–π stacking interactions with Trp391, as well as by hydrogen bonds with additional conserved Asn203, His158 and Tyr347 within the active site, that have already been shown to be of importance for the catalysis [52,53,54,55].

In contrast to quercetin 7-*O*-glucoside, no conformation satisfied geometric criteria for favorable catalysis. While top-ranked poses simulated by AutoDock Vina (Table 4) show really favorable affinity energies similar to quercetin 7-*O*-glucoside, with pose 1 scoring −10.76 kcal/mol, none had the key atoms within range. Most poses showed a preference for the ligand to be mirrored 90° on the *X*-axis and 180° on the *Y*-axis compared to the more linear 7-substituted counterpart. This is probably due to steric hindrance of the sugar in the active site that will not allow for suitable stabilization with residues Asn203, His158 and Tyr347.

Pose 13 had the shortest calculated distance between Glu419 and the anomeric carbon (6.9 Å) and Glu204 and the glycosidic oxygen (3.5 Å). While the acid/base residue is within the catalytic range, the nucleophilic glutamate is positioned much further away than mechanistically possible. The possibility of an effective catalysis at such a distance is non-existent and might explain the absence of activity. Furthermore, the π–π stacking interaction previously seen with Trp391 is also absent with the aromatic ring now directly facing the sugar, reducing the ligand’s stabilization.

It must be argued that AlphaFold models are based on static energy minima and do not consider small molecules or substrates during the building process and that docking might overestimate steric clashes due to the static nature of the program, these results are not a foolproof method to assess substrate affinity with the receptor, but they are an indication that the conformation of 3-substituted ligands might be too bulky for proper accommodation in the active site, resulting in no substrate conversion. The in vivo-induced fit after docking could look different, but in this case, it seems to agree with the experimental results.

## 4. Conclusions

In this study, a *β*-glucosidase from *Fragaria × ananassa* (Fra-*β*GL; *FxaC_10g48920.t1*) was identified, recombinantly expressed, and biochemically characterized in comparison with a commercial *β*-glucosidase from *Prunus dulcis* (Pru-*β*GL). Fra-*β*GL displayed clear substrate selectivity, hydrolyzing the 7-*O*-glucosides of quercetin, kaempferol, and luteolin, while no detectable activity was observed toward the tested 3-*O*-glucosides, anthocyanin glucosides, arbutin, or phloridzin under the assay conditions.

Kinetic characterization further demonstrated differences between Fra-*β*GL and Pru-*β*GL. Fra-*β*GL exhibited higher catalytic efficiency toward all four substrates subjected to kinetic analysis. The highest catalytic efficiency of Fra-*β*GL was observed with the synthetic substrate *p*-nitrophenyl-*β*-D-glucopyranoside (*p*NP-Glc), whereas among the naturally occurring flavonoid glucosides, quercetin 7-*O*-glucoside showed the highest catalytic efficiency. Fra-*β*GL was approximately fivefold more catalytically efficient toward quercetin 7-*O*-glucoside than Pru-*β*GL. The two enzymes also differed in thermal stability, with Pru-*β*GL retaining greater residual activity at elevated temperatures.

Molecular docking provided a structural framework for interpreting the experimentally observed substrate selectivity and suggested that differences in ligand orientation and interactions within the binding site may contribute to the preference of Fra-*β*GL for flavonoid 7-*O*-glucosides over the corresponding 3-*O*-glucosides. These structural observations should, however, be regarded as hypotheses that require further experimental validation.

Collectively, the results establish the in vitro biochemical properties and substrate selectivity of Fra-*β*GL and identify it as a candidate enzyme potentially involved in flavonoid glycoside metabolism in strawberry. However, its physiological function in strawberry fruit cannot be established from the present in vitro data alone. Future studies integrating gene-expression analysis with metabolite profiling and in planta functional approaches, such as gene silencing or overexpression, will be required to determine the contribution of Fra-*β*GL to flavonoid metabolism and its potential relationship with fruit composition and quality.

## Figures and Tables

**Table 1:** Substrates used for enzymatic activity assays, their detection methods, corresponding detection wavelengths, and observed activity results. “+”, detectable substrate hydrolysis accompanied by formation of the corresponding aglycone, confirmed against an authentic standard; “-”, no detectable decrease in substrate peak area or formation of the corresponding aglycone relative to the control under the assay conditions.

Substrate	Structure	Detection Method	Wavelength (nm)	Activity
Arbutin	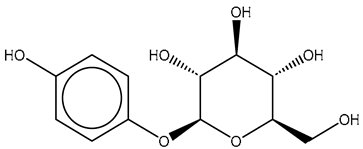	HPLC	290	-
Cyanidin 3-*O*-glucoside	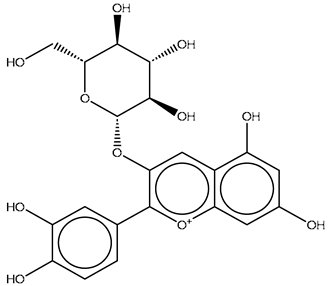	HPLC	520	-
Kaempferol 3-*O*-glucoside	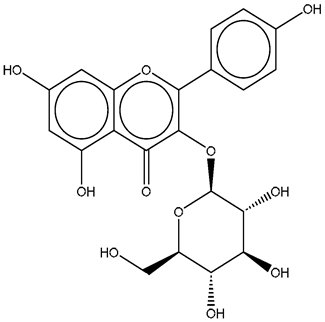	HPLC	360	-
Kaempferol 7-*O*-glucoside	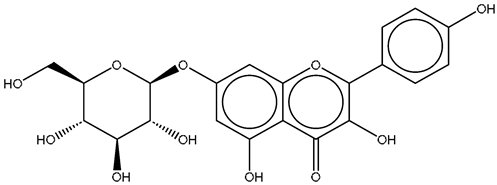	HPLC	360	+
Luteolin 7-*O*-glucoside	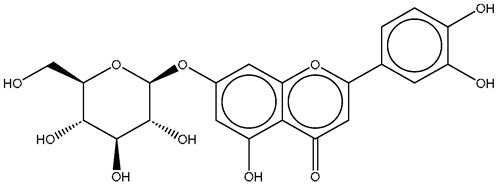	HPLC	340	+
Pelargonidin 3-*O*-glucoside	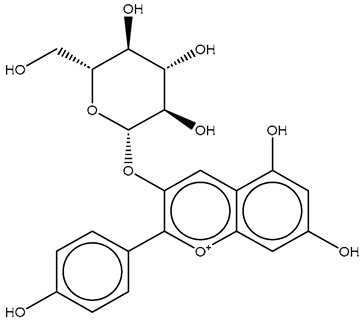	HPLC	520	-
Phloridzin	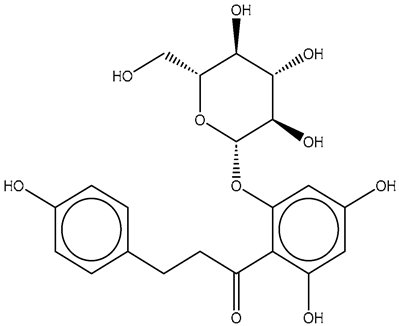	HPLC	290	-
*p*-Nitrophenyl-*β*-d-glucopyranoside	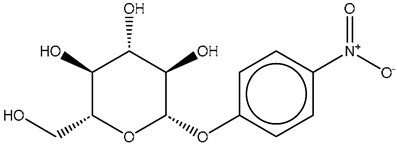	Spectrophotometric	405	+
Quercetin 3-*O*-glucoside	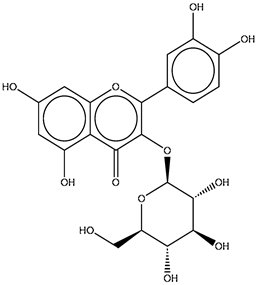	HPLC	360	-
Quercetin 7-*O*-glucoside	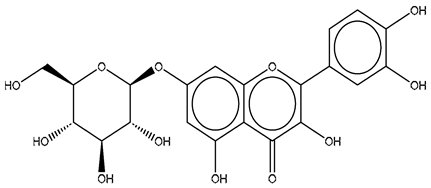	HPLC	360	+

**Table 2:** Kinetic parameters (*K*_m_, *K*_cat_, and catalytic efficiency *K*_cat_/*K*_m_) of Pru-*β*GL and Fra-*β*GL towards active flavonoid glucosides and the synthetic substrate *p*-nitrophenyl-*β*-D-glucopyranoside.

Substrate	*K*_m_ (mM)	*K*_cat_ (s^−1^)	*K*_cat_/*K*_m_ (mM^−1^·s^−1^)
Kaempferol 7-*O*-glucoside (*P. dulcis*)	0.49 ± 0.032	0.237 ± 0.011	0.482 ± 0.039
Kaempferol 7-*O*-glucoside (*F.* × *ananassa*)	0.89 ± 0.158	1.431 ± 0.212	1.602 ± 0.369
Luteolin 7-*O*-glucoside (*P. dulcis*)	0.79 ± 0.136	0.373 ± 0.042	0.47 ± 0.097
Luteolin 7-*O*-glucoside (*F.* × *ananassa*)	0.67 ± 0.057	1.399 ± 0.091	2.09 ± 0.225
*p*-Nitrophenyl-*β*-d-glucopyranoside (*P. dulcis*)	5.91 ± 0.35	0.9 ± 0.03	0.15 ± 0.01
*p*-Nitrophenyl-*β*-d-glucopyranoside (*F.* × *ananassa*)	5.93 ± 0.29	60.33 ± 1.17	10.18 ± 0.32
Quercetin 7-*O*-glucoside (*P. dulcis*)	0.05 ± 0.003	0.043 ± 0.003	0.824 ± 0.071
Quercetin 7-*O*-glucoside (*F.* × *ananassa*)	0.06 ± 0.004	0.23 ± 0.02	4.14 ± 0.47

**Table 3:** Affinity values (kcal/mol), RMSD lower bound and RMSD upper bound for each of the 20 poses calculated by AutoDock Vina for the ligand quercetin 7-*O*-glucoside.

Pose	Affinity (kcal/mol)	RMSD Lower Bound (l.b.)	RMSD Upper Bound (u.b.)
1	−10.76	0	0
2	−10.29	1.347	7.967
3	−9.942	2.278	4.773
4	−9.941	2.252	4.725
5 (depicted in Figure 7)	−9.911	1.015	2.303
6	−9.012	2.178	5.473
7	−8.779	2.18	8.929
8	−8.365	2.293	3.142
9	−8.278	1.929	8.873
10	−8.159	2.146	5.02
11	−8.158	2.084	2.847
12	−7.952	1.616	2.504
13	−7.733	3.258	9.672
14	−7.597	4.84	8.028
15	−7.575	3.052	9.738
16	−7.512	2.345	4.477
17	−7.506	2.557	9.219
18	−7.374	2.603	5.089
19	−7.064	1.959	5.238
20	40.14	2.749	9.385

**Table 4:** Affinity values (kcal/mol), RMSD lower bound and RMSD upper bound for each of the 20 poses calculated by AutoDock Vina for the ligand quercetin 3-*O*-glucoside.

Pose	Affinity (kcal/mol)	RMSD Lower Bound (l.b.)	RMSD Upper Bound (u.b.)
1	−10.76	0	0
2	−10.37	1.33	7.978
3	−10	2.265	4.719
4	−9.952	0.9921	2.298
5	−9.922	2.295	4.736
6	−8.934	2.211	5.516
7	−8.804	2.162	8.946
8	−8.584	2.706	9.372
9	−8.218	1.946	8.94
10	−8.19	2.155	4.802
11	−8.083	2.245	4.277
12	−7.988	2.12	5.076
13 depicted in Figure 8	−7.627	3.347	9.743
14	−7.547	2.573	9.217
15	−7.166	2.542	5.11
16	−7.097	1.793	2.897
17	−6.745	1.977	5.264
18	−6.089	2.984	9.602
19	−3.976	2.312	4.423
20	110.5	1.889	2.622

## Data Availability

The original contributions presented in the study are included in the article/Supplementary Materials, further inquiries can be directed to the corresponding author.

## Supplementary Materials

The following supporting information can be downloaded at https://www.mdpi.com/article/10.3390/foods15183290/s1, Supplementary sequences: *β*-glucosidase (*β*GL) sequences of *Fragaria vesca* retrieved from NCBI; Table S1: Maximum reaction velocities ($V_{max}$, µmol s^−1^) of Pru-*β*GL and Fra-*β*GL toward kaempferol 7-*O*-glucoside, luteolin 7-*O*-glucoside, quercetin 7-*O*-glucoside, and *p*-nitrophenyl-*β*-D-glucopyranoside; Table S2: pH dependence of Fra-*β*GL and Pru-*β*GL activity at 30 °C; Table S3: Temperature dependence of Fra-*β*GL and Pru-*β*GL activity at pH 4.5; Table S4: Thermal stability of Fra-*β*GL and Pru-*β*GL at pH 4.5.
