# Supplementary material for: Characterization of a β-Glucosidase from Fragaria × ananassa: Substrate Specificity, Catalytic Efficiency, and Structural Insights
Source: Foods. 2026 Sep 17;15(18):3290. doi: 10.3390/foods15183290 (PMC13606037; doi:10.3390/foods15183290)
Supplement: Supplementary file 1 [file foods-15-03290-s001.zip › foods-4538405-supplementary.pdf]

Supplementary sequences. *β*-Glucosidase (*β*GLs) sequences of *Fragaria vesca* retrieved from NCBI (Sequence Read Archives (SRAs))

>lcl|KF697198.1_prot_AHC04401.1_1 [gene=BG1] [protein=beta-glucosidase 1] [protein_id=AHC04401.1] [location=1..1512] [gbkey=CDS]
MGWKRGIALFIAFQVIIGCSSESNSEINRGSFPKGFVFGTASSAFQYEGAVKEDGRGPSVWDIFSHTFGKITDFSNADVAVDQYHLYDGDVQLMKDMGMDAYRFSISWSRIFPNGTGQINQAGVD
HYNNLINSLLAKGIEPYVTLYHWDLPQALEDRYTGWLDAQIIKDFAVYAETCFQKFGDRVKHWITFNEPHTFAVQGYDVGLQAPGRCSLLGSLFCRAGNSATEPYIVAHNVILSHATVANIYRKK
YKSKQRGSIGASFDVIWYRSATNSTADIEATQRAQDFQLGWFLDPFIFGDYPFSMRSRVGSRLPKFSKSESTLIKGSLDFVGINHYTTFYASNDSSHIIGLLNDSLSDSGAITLPFKDGKAIGDK
ANSIWLYIVPEGMRSLTNYIKKMYGNPPVIITENGMDDPNSPFISLKDALKDEKRISYHRDYLSNLLASIKEDGCNVNGYFAWSLLDNWEWAAGYTSRFGLYFIDYKDKLKRYPKDSVQWFKNFL
TST

>lcl|XM_004309432.2_prot_XP_004309480.1_1 [gene=LOC101310768] [db_xref=GeneID:101310768] [protein=beta-glucosidase 42] [protein_id=XP_004309480.1] [location=245..1714] [gbkey=CDS]
MAKIELLKEYEQADEISRRAFPPNFVFGVATSAYQVEGACREGNRGPSIWDAFTHTKGKIIDGSNGDIAVDQYHRYKEDVDLIAKLGFAAYRFSISWSRIFPDGLGTTVNEDGIAYYNNIINALL
EKGIQPYVTLYHWDLPLYLHESMGGWLNKQIVKFFSVYADTCFANFGDRVKDWITINEPLQTSVNGYGYGIFAPGRHEHASTEPYLVAHHQLLAHAAAVSIYRSKYKDKQGGQIGIAVDCEWAEA
NSDKTEDKIAAARRLDFQLGWYLDPIYSGEYPKVMRERLGDRLPIFSKEDKDLLKNSLDFVGLNHYTSRFIAHVKDSPEEGDFYKAQEMERIDKWEGGEIIGEKAASEWLYVVPWGIHKVLNYIA
QRYNSPPIYITENGMDDEDDDSSPLNEMLDDKLRVSYFKRYLAAVSDAMRDGADVRGYFAWSLLDNFEWAQGYTKRFGLVYIDYKNGLSRHPKSSAYWFSRFLKNGEGRDGKDD

>lcl|XM_004303949.2_prot_XP_004303997.1_1 [gene=LOC101297272] [db_xref=GeneID:101297272] [protein=beta-glucosidase 13-like] [protein_id=XP_004303997.1] [location=739..2448] [gbkey=CDS]
MRMRPSSCSMEHYCWFCVFIGVETREYLPLFNESGKIQASEQIVRGCWFCRFYTMISCLTARTIEACFRICCLIFELAGQSSPPAGRGALDDNAKLANLNEAQTSTRQETISRKDFPADFKFGCA
TSALQTEGCGTEGGRGPATWDEYIQDGTPPIKIAVDSYHRYKEDVQLLKAMGVDTYRFSMSWSRILPEGTIDGGVNQEGIDFYNNFINDLIANGITPFATLFHFDLPTALNTKYTGFLSIDIVED
FKAYADLCFKTFGDRVKHWATINEPQVWGQYGFTNPDPNANAATDPFLSTHHVLLAHAAAAKLYKNTYQASQGGEIGIPLVVEWFEPFEDTIPDKAAARRAFDFLVGWFMEPLVYGDYPFIMKTL
VKDGLPNFTDEQKELVKGSYDYIGVNYYTSRFAAPIAIVDGEEITDSDQYQHVNITVDGPDGEPIGTPTPGSSEIYVYPQGLKKALILLKEYGNPKFYITENGYPDKRDDTIPISTALIDDARIH
HIKDHLTAIKEAMNTGTNVKAYLMWAMLDCVEMGSGYQVRFGLNFTNYLGNLNRTAKKSAGWLKTFLTS

>lcl|XM_004303947.2_prot_XP_004303995.1_1 [gene=LOC101296690] [db_xref=GeneID:101296690] [protein=beta-glucosidase 24-like] [protein_id=XP_004303995.1] [location=85..1767] [gbkey=CDS]
MALSLSFSLTTSATTAPLSSFKRLPSRIPTVKALGVFPSKGIDHKRAIRIRPCSSLGDLTSALTSAVTTTVAVAVDTAAAVVDTTTTTTTTTETAEVTRADFPADFKFGCATSAFQTEGSGTEGG
RGPATWDDYIQDTTVDGTEIAVDSYNRYKDDVQLLKNMGVDTYRFSMSWSRLLPDGTIDGGINQEGIDFYNNFIDELIANGITPFVTLFHFDLPTALKDKYNGFLSIDIVEDFKAYADLCFKTFG
DRVKYWATINEPQVWGQYGFTNTAADANAATDPYVSSHNVILAHATAAKLYKNTYQSTQGGEIGIPIVVEWFEPFEDTPQDVFAAKRAFDFLTGWFLEPLVYGDYPFVMKALVRDGLPEFTDEQK
ELVKGSYDYIGVNYYTSRFATSVPITSNDVYTTMDQYQHATITVEGSDGEPIGDATPGSSSIYVYPEGLRKGLTLLKEYNNPKIYVTENGYPDARDDTLSVEEASVDDARIQHIKDHLAAVKAAR
DAGADVQGYLMWALMDCVEMGSGYAVRFGLNYTDYLNDLVRTPKKSAAWLKDFLASTTST

>lcl|XM_011468950.1_prot_XP_011467252.1_1 [gene=LOC101292823] [db_xref=GeneID:101292823] [protein=beta-glucosidase 24-like isoform X2] [protein_id=XP_011467252.1] [location=838..2130] [gbkey=CDS]
MSTTIDSYKRYKQDVKAVKALGVDSYRFSISWTRILPKGTLSGGINQEGVDHYNNLIDELIKYGITPYVTILHFDPPQALTVKYGGFLSRSFVDDFKDYSELCFKIFGDRVKNWITINEPFIIAK
MGYDLGVAAPGRCSIPGGDFDYCTAGNSSTEPYIVAHNLLLAHATVVKLYREKFQTKQGGQIGCSHVGTYIEAYSHSAKDKAAAKRILDFELGWFMEPIVYGCYPKSMRRLVKKRLPSFTKEEKK
LIMGSFDFIGINYYTSRYGKNIPARPHMPHFSRNDPLASSEFKNVNGVPIGPQADGSTFIFSYPQGLKKLLKFMKQKYQSPKIYITENGITVAKNNRRGLDVALKDPHRIECILRHLYMIKQAIK
KGVNVVGYFHWALFDDFEWGEGYTPRFGLYYVDYKDNLKRIPKESAKWLPKFLNG

>lcl|XM_011468949.1_prot_XP_011467251.1_1 [gene=LOC101292823] [db_xref=GeneID:101292823] [protein=beta-glucosidase 24-like isoform X2] [protein_id=XP_011467251.1] [location=1955..3247] [gbkey=CDS]
MSTTIDSYKRYKQDVKAVKALGVDSYRFSISWTRILPKGTLSGGINQEGVDHYNNLIDELIKYGITPYVTILHFDPPQALTVKYGGFLSRSFVDDFKDYSELCFKIFGDRVKNWITINEPFIIAK
MGYDLGVAAPGRCSIPGGDFDYCTAGNSSTEPYIVAHNLLLAHATVVKLYREKFQTKQGGQIGCSHVGTYIEAYSHSAKDKAAAKRILDFELGWFMEPIVYGCYPKSMRRLVKKRLPSFTKEEKK
LIMGSFDFIGINYYTSRYGKNIPARPHMPHFSRNDPLASSEFKNVNGVPIGPQADGSTFIFSYPQGLKKLLKFMKQKYQSPKIYITENGITVAKNNRRGLDVALKDPHRIECILRHLYMIKQAIK
KGVNVVGYFHWALFDDFEWGEGYTPRFGLYYVDYKDNLKRIPKESAKWLPKFLNG

>lcl|XM_011468948.1_prot_XP_011467250.1_1 [gene=LOC101292823] [db_xref=GeneID:101292823] [protein=beta-glucosidase 24-like isoform X2] [protein_id=XP_011467250.1] [location=2051..3343] [gbkey=CDS]
MSTTIDSYKRYKQDVKAVKALGVDSYRFSISWTRILPKGTLSGGINQEGVDHYNNLIDELIKYGITPYVTILHFDPPQALTVKYGGFLSRSFVDDFKDYSELCFKIFGDRVKNWITINEPFIIAK
MGYDLGVAAPGRCSIPGGDFDYCTAGNSSTEPYIVAHNLLLAHATVVKLYREKFQTKQGGQIGCSHVGTYIEAYSHSAKDKAAAKRILDFELGWFMEPIVYGCYPKSMRRLVKKRLPSFTKEEKK
LIMGSFDFIGINYYTSRYGKNIPARPHMPHFSRNDPLASSEFKNVNGVPIGPQADGSTFIFSYPQGLKKLLKFMKQKYQSPKIYITENGITVAKNNRRGLDVALKDPHRIECILRHLYMIKQAIK
KGVNVVGYFHWALFDDFEWGEGYTPRFGLYYVDYKDNLKRIPKESAKWLPKFLNG

>lcl|XM_011468948.1_prot_XP_011467250.1_1 [gene=LOC101292823] [db_xref=GeneID:101292823] [protein=beta-glucosidase 24-like isoform X2] [protein_id=XP_011467250.1] [location=2051..3343] [gbkey=CDS]
MSTTIDSYKRYKQDVKAVKALGVDSYRFSISWTRILPKGTLSGGINQEGVDHYNNLIDELIKYGITPYVTILHFDPPQALTVKYGGFLSRSFVDDFKDYSELCFKIFGDRVKNWITINEPFIIAK
MGYDLGVAAPGRCSIPGGDFDYCTAGNSSTEPYIVAHNLLLAHATVVKLYREKFQTKQGGQIGCSHVGTYIEAYSHSAKDKAAAKRILDFELGWFMEPIVYGCYPKSMRRLVKKRLPSFTKEEKK
LIMGSFDFIGINYYTSRYGKNIPARPHMPHFSRNDPLASSEFKNVNGVPIGPQADGSTFIFSYPQGLKKLLKFMKQKYQSPKIYITENGITVAKNNRRGLDVALKDPHRIECILRHLYMIKQAIK
KGVNVVGYFHWALFDDFEWGEGYTPRFGLYYVDYKDNLKRIPKESAKWLPKFLNG

>lcl|XM_011468947.1_prot_XP_011467249.1_1 [gene=LOC101292823] [db_xref=GeneID:101292823] [protein=beta-glucosidase 24-like isoform X1] [protein_id=XP_011467249.1] [location=108..1640] [gbkey=CDS]
MECIANVTVNAEKKLQALHINASNPKELKVKWSDFPSDFVFGVSTAAAQIEGTTNKAGRGPSVWDEFVKTFPERIADHSTMSTTIDSYKRYKQDVKAVKALGVDSYRFSISWTRILPKGTLSGGI
NQEGVDHYNNLIDELIKYGITPYVTILHFDPPQALTVKYGGFLSRSFVDDFKDYSELCFKIFGDRVKNWITINEPFIIAKMGYDLGVAAPGRCSIPGGDFDYCTAGNSSTEPYIVAHNLLLAHAT
VVKLYREKFQTKQGGQIGCSHVGTYIEAYSHSAKDKAAAKRILDFELGWFMEPIVYGCYPKSMRRLVKKRLPSFTKEEKKLIMGSFDFIGINYYTSRYGKNIPARPHMPHFSRNDPLASSEFKNV
NGVPIGPQADGSTFIFSYPQGLKKLLKFMKQKYQSPKIYITENGITVAKNNRRGLDVALKDPHRIECILRHLYMIKQAIKKGVNVVGYFHWALFDDFEWGEGYTPRFGLYYVDYKDNLKRIPKES
AKWLPKFLNG

>lcl|XM_004299497.2_prot_XP_004299545.1_1 [gene=LOC101312349] [db_xref=GeneID:101312349] [protein=beta-glucosidase 44-like] [protein_id=XP_004299545.1] [location=231..1769] [gbkey=CDS]
MRTTPVLVLFWVSLVVVIQYVAGAAKHNGAPFPEAEGGLRASFPKGFVFGTATSAYQVEGMANKEGRGPSIWDTFVKIPGIVANNATGDVTVDQYHRYKEDIDLLANLNFDAYRFSISWSRIFPE
GTGKVNWKGVAYYNRLIDYLLKRGITPYANLYHYDLPLALEKKYLGLLSDRVVKDFADFADFCFKTYGDRVKNWMTFNEPRVVAALGYDTGFFAPGRCSKAYGNCTAGNSGTEPYIAAHHLILSH
AAAVQRYREKYQKTQKGKIGILLDFVWYEPLTRSKADRYAAQRARDFHVGWFIHPIVYGEYPKTIQNIVGNRLPKFTKEEVKMVKGSMDFVGINEYTAYYIYAPDKNQTKSNALGYQNDWNAGFA
YEKNGVPIGPRAYSSWLYQVPWGMYKCITYIKEHYGNPPVILSENGMDNPGNQTLPKALHDTNRIHYYRSYLEQLKKAVDEGANVVGYFAWSLLDNFEWRLGYTSRFGIVYVDFTTLKRYPKMSA
YWFKKLLTKKKH

>lcl|XM_004299498.2_prot_XP_004299546.1_1 [gene=LOC101312635] [db_xref=GeneID:101312635] [protein=beta-glucosidase 44-like] [protein_id=XP_004299546.1] [location=52..1593] [gbkey=CDS]
MRTAVLLLVLLLSGFVVVIQYAAGAAKYNGAPFPEAEGGLRTGFPKGFVFGTATSAYQVEGMANKEGRGPSIWDAFVKIPGNIANNDTADVTADEYHRYKEDIDIMQDLNFDVYRFSISWSRIFP
DGTGKVNLKGVAYYNRLINYLLERGITPYANLHHYDLPLALEQEYLGFLSDRVVKDFADYAEFCFKTYGDRVKYWTTFNEPRVVAALGYDNALNPPARCSKAYGNCTVGNSGTEPYIVAHHLLLS
HAAAVHRYREKYQKTQKGSVGILLDFNWYEPLTSSKDDRDAAQRARDFHIGWFIHPIVYGEYPKTMQHIVGDRLPKFTKEEVKMVKGSIDFVGINQYTAYYMYDPHQNKTKPNALGYQNDWNAGF
AFEKNGVLIGPQAYSSWLYQVPWGLYKCVTYIKKNYGNPTVIISENGMDNPGNQTLQEALHDTNRIDFYRSYLEQLKKAVDEGANVVGYFAWSLLDNFEWRLGYTSRFGIVYVDFTTLKRYPKMS
AYWFKKLLTKKKH

>lcl|XM_011465402.1_prot_XP_011463704.1_1 [gene=LOC105351379] [db_xref=GeneID:105351379] [protein=beta-glucosidase 13-like] [protein_id=XP_011463704.1] [location=1..1611] [gbkey=CDS]
MASSTYALIALFAMVSIAGWPVQATWDGVVPVEANLTVGGYTFRLNLNSPAYLEELNVTKSDFPTDFAFGVGSSAAQIEGAAREFGRGPSTWDHRVDILPGSIHDSDKLSTAIDSYRRYKQDIEL
IKDLGVDSYRFSISWSRILPYGSLSGGINQQGVDHYNSLIDELIKNGIKPYVTIFHFDLPQALEEKYGGFLNRSFVDDFRDYSELCFKLFGDRVKNWFTINEPSVSAVYGYELGIAPPGKCSLQE
GVCVFGTSDNCLVPAGPCNFGGNSSTDPYIAAHNQILAHATAAKLYKEKYQSTHKGEIGIVLVSRHYMPFTKKQEDIAAAKRRFDFTLGWFMDPFVYGDYPKSMKELVKERLPTFSPEEQSLIKG

SLDFVGINYYSSTYARNKAPSKELRYSYDVSADEIGHWPEGLEKVLVHMKKKYQNPKIYITENGLSTTRNDELPFADQLKDPERIAYIVRHLYRINKAIKKGVNVKGYFFWAMFDDFEWGMGFLD
RFGLYHIDFAHNYRRTPKHSAKWFKAFLQSKVARAS

>lcl|XM_011464992.1_prot_XP_011463294.1_1 [gene=LOC105351214] [db_xref=GeneID:105351214] [protein=beta-glucosidase 13-like isoform X3] [protein_id=XP_011463294.1] [location=254..1492] [gbkey=CDS]
MASSTKFSGVVIVAVSAIMASIAGCYARATIPDFNGGVAGKLKLYSSSAFLDELDVSRSDFPADFTFGVATSAAQTEGSAREGGRGPSTWDHRVDILPDAIINSDKFSTAIDSYRRYKEDIKIIK
DLGVNAYRFSISWSRILPKGSLSGGINQEGIDHYNSFIDDLIRNGIEPFVTILHSDLPQALEDKYGGYLNHSFVNDFKDYSEICFKSFGDRVKNWFTINEPTVSAVYGYELGIAPPGKCSLKEGV
CVFGTPDNCLVPAGPCNFGGDSSTEPYIVAHNQILGHATVAKLYKEKYKAQQKGEIGIVLASKYYVPFSNSPEDKVAAERLFDFNLGWFMEPFVYGDYPKSMRELVKERLPSFSAQEQSLIKGCL
DFVGINYYTSSYARHKTPPPLEQFRYSFDISAEETGP

>lcl|XM_011464991.1_prot_XP_011463293.1_1 [gene=LOC105351214] [db_xref=GeneID:105351214] [protein=beta-glucosidase 13-like isoform X2] [protein_id=XP_011463293.1] [location=254..1564] [gbkey=CDS]
MASSTKFSGVVIVAVSAIMASIAGCYARATIPDFNGGVAGKLKLYSSSAFLDELDVSRSDFPADFTFGVATSAAQTEGSAREGGRGPSTWDHRVDILPDAIINSDKFSTAIDSYRRYKEDIKIIK
DLGVNAYRFSISWSRILPKGSLSGGINQEGIDHYNSFIDDLIRNGIEPFVTILHSDLPQALEDKYGGYLNHSFVNDFKDYSEICFKSFGDRVKNWFTINEPTVSAVYGYELGIAPPGKCSLKEGV
CVFGTPDNCLVPAGPCNFGGDSSTEPYIVAHNQILGHATVAKLYKEKYKAQQKGEIGIVLASKYYVPFSNSPEDKVAAERLFDFNLGWFMEPFVYGDYPKSMRELVKERLPSFSAQEQSLIKGCL
DFVGINYYTSSYARHKTPPPLEQFRYSFDISAEETGMKDGDFLGMLHCRTMKGTGQKGCRNMASSTKFSGVVIVAVSAIMASIAGCYARATIPDFNGGVAGKLKLYSSSAFLDELDVSRSDFPAD
FTFGVATSAAQTEGSAREGGRGPSTWDHRVDILPDAIINSDKFSTAIDSYRRYKEDIKIIKDLGVNAYRFSISWSRILPKGSLSGGINQEGIDHYNSFIDDLIRNGIEPFVTILHSDLPQALEDK
YGGYLNHSFVNDFKDYSEICFKSFGDRVKNWFTINEPTVSAVYGYELGIAPPGKCSLKEGVCVFGTPDNCLVPAGPCNFGGDSSTEPYIVAHNQILGHATVAKLYKEKYKAQQKGEIGIVLASKY
YVPFSNSPEDKVAAERLFDFNLGWFMEPFVYGDYPKSMRELVKERLPSFSAQEQSLIKGCLDFVGINYYTSSYARHKTPPPLEQFRYSFDISAEETGMKDGDFLGMLHCRTMKGTGQKGCRN

>lcl|XM_011464990.1_prot_XP_011463292.1_1 [gene=LOC105351214] [db_xref=GeneID:105351214] [protein=beta-glucosidase 13-like isoform X1] [protein_id=XP_011463292.1] [location=255..1910] [gbkey=CDS]
MASSTKFSGVVIVAVSAIMASIAGCYARATIPDFNGGVAGKLKLYSSSAFLDELDVSRSDFPADFTFGVATSAAQTEGSAREGGRGPSTWDHRVDILPDAIINSDKFSTAIDSYRRYKEDIKIIK
DLGVNAYRFSISWSRILPKGSLSGGINQEGIDHYNSFIDDLIRNGIEPFVTILHSDLPQALEDKYGGYLNHSFVNDFKDYSEICFKSFGDRVKNWFTINEPTVSAVYGYELGIAPPGKCSLKEGV
CVFGTPDNCLVPAGPCNFGGDSSTEPYIVAHNQILGHATVAKLYKEKYKAQQKGEIGIVLASKYYVPFSNSPEDKVAAERLFDFNLGWFMEPFVYGDYPKSMRELVKERLPSFSAQEQSLIKGCL
DFVGINYYTSSYARHKTPPPLEQFRYSFDISAEETGMKDGDFLGMDHEGHWPEGLQKLMEYMMEKYQNPKIYISENGLYTTRNDELSLVDQLNDTNRISFLVRHLYRLNKAIKNGVNVKGYFYWS
LFDDFEWGMGFLHRYGLYHIDFDHNNRRIPKQSADWFKAFLQSDQVTRASS

>lcl|XM_004297206.2_prot_XP_004297254.1_1 [gene=LOC101290754] [db_xref=GeneID:101290754] [protein=beta-glucosidase 40] [protein_id=XP_004297254.1] [location=323..1834] [gbkey=CDS]
MGWKRGIALFIAFQVIIGCSSQSNSEINRGSFPKGFVFGTASSAFQYEGAVKEDGRGPSVWDIFSHTFGKITDFSNADVAVDQYHLYDGDVQLMKDMGMDAYRFSISWSRIFPNGTGQINQAGVD
HYNNLINSLLAKGIEPYVTLYHWDLPQALEDRYTGWLDAQIIQDFAVYAETCFQKFGDRVKHWITFNEPHTFAVQGYDVGLQAPGRCSLLGRLFCRAGNSATEPYIVAHNVILSHATVADIYRKK
YKPKQRGSIGASFDVIWYRSATNSTADIEATERAQDFQLGWFLDPFIFGDYPFSMRSRVGSRLPKFSKSESTLIKGSLDFVGINHYTTFYASNDSSHIIGLLNDSLSDSGAIALPFKDGKAIGDK
ANSIWLYIVPEGMRSLMNYIKKKYGNPPVLITENGMDDPNSPFISLKDALKDEKRISYHRDYLSNLLASIKEDGCNVNGYFAWSLLDNWEWGAGYTSRFGLYFIDYKDKLKRYPKDSVQWFKNFL
TST

>lcl|XM_004295179.2_prot_XP_004295227.1_1 [gene=LOC101307288] [db_xref=GeneID:101307288] [protein=beta-glucosidase 13-like] [protein_id=XP_004295227.1] [location=153..1715] [gbkey=CDS]
MASISGGSSFLNIIAISLIVSAFASLVRGDDLSRGQYSTAFLNRSSFPAGFVFGTASSAYQYEGAANLSGRGPSIWDTFTHKYPDKITDGSNGDVAIDSYHRYKEDVAIMKEMGLDVYRFSISWS
RVLPTGKLSGGVNKEGIEYYNNLINELLANGIKPFVTLFHWDLPQPLQDEHGGFISPHIVKHFKAYAELCYREFGDQVKHWITFNEPIALAVAGYGLGALAPGRCSAWINPNCTGGNSTTEPYLV
THYQLLAHAAAVNLYKKHYQESQKGLIGITLVAQWLVPNTTARHDRAAQLRGLDFMLGWYMDPLTNGDYPKSMKSLVGDRLPKFKKEQSKLLKGSFDFIGLNYYTSNYVSDAPQLVKVVNASFMT
DSLATMSPLRNGIPIGPKAASEDLYIYPRGIRDILLYTKRKYNNPLIYITENGVDEFNDPKLTLEEALADNQRIDYHFRHLYYLQEAIKDGVNVKGYFAWSFLDNFEWTLGYTVRFGINYVDYKD
GLKRYPKHSALWFKNFLKKY

>lcl|XM_011463607.1_prot_XP_011461909.1_1 [gene=LOC105350726] [db_xref=GeneID:105350726] [protein=beta-glucosidase 13-like] [protein_id=XP_011461909.1] [location=236..1840] [gbkey=CDS]
MALRLGLILLLLVLVCTAAVTTGRAASSRHRDSSSLHRSSFPAGFVFGTASSAYQYEGAANEGGRGPSIWDTYTHKYPEKIHDHSTADVAIDEYHRYKEDIRIMKDMGLDAYRFSISWSRLLPNG
KLSGGVNKEGIKYYNNLINELLANGLVPYVTLFHWDTPQALEDEFSGFLSPKIVDHFKDYADLCYKEFGDRVRYWITLNEPWSYTNGGYVIGTFAPTRCSDWQNLNCTGGDSGTEPYLATHYQLL
AHAAAVKLYRDKYQASQKGVIGITLLSYWFVPYSDAKCDVDAAERALDFMYGWFMDPLINGDYPHSMRYLVAERLPKFTKEESMLLKGSFDFIGVNYYTANFAAYNAPRFNATHPNSNNPSYLTD
ALVNLSVERDGVPIGPKTALDWLYVYPEGFRELLLYTKNKYNDPVIYVTENGVNELNDPGLSLEESLTDHHRIDYYDAHLSYLLSAMKDGVNVKGYFAWSLFDNFEWNTGYTCRFGFNFIDYRNG
LKRYPKLSAGWFKNFLQKTSVKLDTSSSREFQSIMALRLGLILLLLVLVCTAAVTTGRAASSRHRDSSSLHRSSFPAGFVFGTASSAYQYEGAANEGGRGPSIWDTYTHKYPEKIHDHSTADVAI
DEYHRYKEDIRIMKDMGLDAYRFSISWSRLLPNGKLSGGVNKEGIKYYNNLINELLANGLVPYVTLFHWDTPQALEDEFSGFLSPKIVDHFKDYADLCYKEFGDRVRYWITLNEPWSYTNGGYVI
GTFAPTRCSDWQNLNCTGGDSGTEPYLATHYQLLAHAAAVKLYRDKYQASQKGVIGITLLSYWFVPYSDAKCDVDAAERALDFMYGWFMDPLINGDYPHSMRYLVAERLPKFTKEESMLLKGSFD
FIGVNYYTANFAAYNAPRFNATHPNSNNPSYLTDALVNLSVERDGVPIGPKTALDWLYVYPEGFRELLLYTKNKYNDPVIYVTENGVNELNDPGLSLEESLTDHHRIDYYDAHLSYLLSAMKDGV
NVKGYFAWSLFDNFEWNTGYTCRFGFNFIDYRNGLKRYPKLSAGWFKNFLQKTSVKLDTSSSREFQSI

>lcl|XM_004296295.2_prot_XP_004296343.1_1 [gene=LOC101300711] [db_xref=GeneID:101300711] [protein=beta-glucosidase 13-like] [protein_id=XP_004296343.1] [location=105..1685] [gbkey=CDS]
MALRLGVGTLLFGLLLVLSSTGINSHSSRKTSPQKKSRNDTSFSRSNFPAGFVFGTASAAYQFEGAAKEGGRGPCVWDAFAHKYPEKIKDHSSGDVATDQYHRYKEDVKMLKDMGWDAYRFSISW
SRLLPKGKLSGGVNQEGITYYKNLLNELVANGLKPFVTLFHWDTPQALEEEFGGFLSPQIVNRFQDFAELCYKEFGDKVKHWTTLNEPLTYSVSGYATGAMAPGRCSAWQNPKCTGGDSGTEPYL
VTHHLLLAHAAAVKVYKEKFQGKQKGLIGIVLNTGWFVPYSDSKRDQDAAQRAIDFSLGWHMDPVAHGDYPQIMRSLVGNRLPKFTKEQSHMLIGSFDFIGMNYYSGGYSADLPNKPDPKQPSYT
SDSLVNSTSDRNGIPIGPVAASSWLLVYPQGFLDLMLYMKKKYNNPTMYITENGVDEVNDPTWSLEKALDDSKRIEYYDQHLQKLQAAIKAGVKVKGYFAWSFLDNFEWADGYTVRFGLVYVDFK
NGLKRHPKKSANWFKNFLQKSTKSGK

>lcl|XM_004295180.2_prot_XP_004295228.1_1 [gene=LOC101307573] [db_xref=GeneID:101307573] [protein=beta-glucosidase 12-like] [protein_id=XP_004295228.1] [location=669..2240] [gbkey=CDS]
MALRLGVGTLLFGLLLAVSSKGITGHSSRKASHKRMSHNDSCLNRSTFPAGFMFGTASAAYQFEGAANEGGRGPCVWDAFTHKYPEKIKDRSSGDMAADQYHRYKEDVKMLKEMGWDAYRFSISW
SRLLPNGKLSGGVNQEGIKYYKNLINELVSHGLIPFVTLFHWDTPQALEEDYGGFLSPQIVKHFQDYAELCYKEFGDKVKHWTTLNEPHTYSVNGYATGGSAPGRCSAWKDPNCTGGDSGTEPYL
VSHHLLLAHAAAVQVYKEKFQAKQKGLIGIVVNTGWFMPYSDSKRDHDAAQRTMDFNLGWFMDPLTHGDYPQIMRSLVGNRLPKFTKEQSDKLKGSFDFIGMNYYSGGYSADLHTKPDPKQPSYT
SDSLVNVTFERNGVPIGPVAASSWLLVFPQGFLDLLLYIKNKYNNPTMYITENGWDEVNDPTWSLEKALDDSTRIEFYDQHLRKLQAAIKAGVKVKGYFAWSFLDNFEWADGYTVRFGLVYVDYK
NEQKRHPKKSVNWFKNFLHKSKE

>lcl|XM_011463606.1_prot_XP_011461908.1_1 [gene=LOC101300996] [db_xref=GeneID:101300996] [protein=beta-glucosidase 12-like isoform X2] [protein_id=XP_011461908.1] [location=373..1908] [gbkey=CDS]
MALQGILLLGFLLVLGCAVRISGATNSKQFDNASLNRSSFPAGFIFGTASAAYQYEGAAKEGGRGPSIWDTYTHKYPEKIFDHSSGDVAIDQYHRYKEDVRTMKDLGFDAYRLSFSWSRLLPNGK
LSGGVNKEGIKYYNNLINELLANGVKPFVTIFHWDLPQALEDEYGGFLSPKIVNHFQDYAELCFKEFGDRVKHWITLNEPHSYSRNGYATGKFAPGRCSAWQKRNCTGGDSGTEPYLASHYQLLA
HAAAMKLYRHKYQAKQKGLIGITINSHWFVPYSDSKQDQAAALRALDFMFGWYMDPMTNGDYPSSMRALVGNRLPKFTKEQSKMLKGSFDFLGLNYYTGNYAANIPFRNVKEASYTTDSLANLTA
VRNGIPIGRPAASDWLHFYPQGFREILHYTKKKYNNPLIYITENGWDDRGDPTLSLEEALRDNLRIEYYNRHLDELQKAIKAGVKVKGYFAWSLFDNFEWADGYSCRFGINSVDYKNGLKRHPKH
SAAWFKNFLQK

>lcl|XM_011463605.1_prot_XP_011461907.1_1 [gene=LOC101300996] [db_xref=GeneID:101300996] [protein=beta-glucosidase 12-like isoform X1] [protein_id=XP_011461907.1] [location=373..1920] [gbkey=CDS]
MALQGILLLGFLLVLGCAVRISGATNSKQFDNASLNRSSFPAGFIFGTASAAYQYEGAAKEGGRGPSIWDTYTHKYPGKKSEKIFDHSSGDVAIDQYHRYKEDVRTMKDLGFDAYRLSFSWSRLL
PNGKLSGGVNKEGIKYYNNLINELLANGVKPFVTIFHWDLPQALEDEYGGFLSPKIVNHFQDYAELCFKEFGDRVKHWITLNEPHSYSRNGYATGKFAPGRCSAWQKRNCTGGDSGTEPYLASHY
QLLAHAAAMKLYRHKYQAKQKGLIGITINSHWFVPYSDSKQDQAAALRALDFMFGWYMDPMTNGDYPSSMRALVGNRLPKFTKEQSKMLKGSFDFLGLNYYTGNYAANIPFRNVKEASYTTDSLA
NLTAVRNGIPIGRPAASDWLHFYPQGFREILHYTKKKYNNPLIYITENGWDDRGDPTLSLEEALRDNLRIEYYNRHLDELQKAIKAGVKVKGYFAWSLFDNFEWADGYSCRFGINSVDYKNGLKR
HPKHSAAWFKNFLQK

>lcl|XM_011463603.1_prot_XP_011461905.1_1 [gene=LOC101300431] [db_xref=GeneID:101300431] [protein=beta-glucosidase 12-like] [protein_id=XP_011461905.1] [location=31..1569] [gbkey=CDS]
MAFQGTLNFVILLVFSYGLATSIATSPSHYDVTAINRSTFPAGFIFGTASSAYQYEGAAKEDGRGPSIWDTYTHTFPDKIKDGSNGDVAIDAYHRYKEDVGIMKDMGFDAYRFSISWSRLLPNGT
LSGGVNKEGIKYYNNLINELLANGLKPFVTLFHWDLPQALEDAYGGFLSPQIVDHFQDYAELCFKEFGDRVKDWITLNEPWSYARGGYVIGTFAPCRCSDWQNLNCTGGDSGTEPYLASHYQLLA
HAAAVKSYKEKYQAEQKGVIGITILSHWFVPFSDAKHDKEAALRALDFMFGWYMDPLTYGEYPHSMRSLVGDRLPEFTKEQSEMLKGSFDFLGLNYYTANYATYAPELKNAANPSYLTDAIAILS
TERNGIPIGPQSASDWLYVYPEGIRELLLYTKEKYNDPLIYITENGRDEHNDPKLTLEEALADTHRIDFYYQHLHYLHEAIKDGVNVKGYFAWSLFDNFEWNMGYSVRFGINYVDYNDGLKRYPK
LSAHWFKNFLGN

>lcl|XM_011463602.1_prot_XP_011461904.1_1 [gene=LOC101300134] [db_xref=GeneID:101300134] [protein=beta-glucosidase 12-like] [protein_id=XP_011461904.1] [location=163..1710] [gbkey=CDS]
MKIQGSLLPAIVLLLLGFGLRNGKASDTPPAHYDTAFLNRTSFPAGFIFGTASSSYQYEGAAQDGGRGPSIWDTYTHRYPDKIKDGSNGDVANDEYHRYKEDVGIMKNMGLDAYRFSISWSRLLP
NGKLSGGVNKKGVKYYNNLINELLRNGIKPFVTLFHWDLPQTLEDEYGGFLSPHIVNHFRDYAELCYKEFGDRVKHWITFNEPYSYSAGGYAAAILAPGRCSDWQQLNCTGGDSGTEPYLVAHHQ
LLAHAAAVKLYRQKYQSSQKGLIGITLVSNWYVPVSEAKHHKNAALRALDYMLGWFMDPLTNGDYPHSMRSLVGNRLPKFTKEQSKLLIGSFDFLGLNYYTANYAAYAPQHNSVNASYLTDARAS
LSSVLNGIPIGPKAASDWLYVYPRGIRDLLLYTKTKYNDPLIYITENGIDEYNDPQLSLEEALNDTQRIDYYYRHLYYLQRAIKDGVNVKGYFAWSLLDNFEWNSGYTVRFGINYVDYKNGQTRH
PKLSAHWFKGFLKKT

>lcl|XM_004295175.2_prot_XP_004295223.1_1 [gene=LOC101306214] [db_xref=GeneID:101306214] [protein=beta-glucosidase 12-like] [protein_id=XP_004295223.1] [location=53..1606] [gbkey=CDS]
MAMQRFLFLGIVLLLVAGFRLTNSEALSPSRTPIYDTAFLNRTSFPEGFVFGTASAAYQYEGAAKEGGRGPSIWDTYTHEHPERITDGSNGDVANDQYHRYKEDVRIMKDMNLDAYRFSISWSRL
LPNGKLSGGVNKEGVKYYNNLINELLSKGLQPYVTLFHWDLPQTLEDEYGGFLSPNIVNHFRDFSELCFKEFGDRVKYWITLNEPWTYSNGGYALGSLAPGRCSAWQQLNCTGGDSSTEPYLVAH
NLLLSHAAAVKLYRQKYQAYQKGVIGITIVSHWFIPISDATHHKNAALRTLDFMFGWFMDPLTNGDYPHSMRSLVGNRLPKFTKEQSKLLIGSFDFLGLNYYTTYYASYTPQNNSLNASYVTDAR
STQSFALNGVPIGPQAASSWLYVYPKGLRDLLLYTKTKYNDPLIYITENGIDEFNDPKLSLEEALFDNQRVDYYFRHLYYLQKAIEDGVKVKGYFAWSLLDNFEWASGYTVRFGINYVDYKNGNK
RHPKLSAHWFKNFLKNY

>lcl|XM_004294358.2_prot_XP_004294406.1_1 [gene=LOC101291030] [db_xref=GeneID:101291030] [protein=beta-glucosidase 11-like] [protein_id=XP_004294406.1] [location=283..1839] [gbkey=CDS]
MVLQLELLLFLSFITLIIGIPLNLSLDLELGAGDHGFKRADFPAPPDFVFGASSSAYQVEGAADQDGRTPSIWDTYAHAGKMHGATGDVACDEYHRYKEDVQLMVDTGLEAYRFSISWSRLISNG
RGPVNPKGLQYYNNLIDQLISKGIQPHVTLHHADLPQALDDEYGGWVSPQIIKDFTAYANVCFAQFGDRVKHWTTMNEPNVFVLGGYDIGFLPPSRCSAPFGVNCSRGDSTTEPYLAIHNFLLSH
ASAAALYKQTYQHKQCGFIGINVFSYWFIPRTETHQDQAAAQRAMDFYFSWVVHPLVYGEYPGVMKKNAGSRLPTFTSAESALVKGSFDFIGLNYYNTLYADDNSAALNLQTRDYVADSAIKISP
LDQDRSTFEYPITPWGMEELLEYIKQNYGNPLLYIHENGQRTRRNSSLEDWSRIEYLHGHIHSLLNVIRNGSNARGYFTWSFLDTLELLDGLESSYGLYYIDVDDPDLKRQPKLSAHWYSKFLKN
KNFTPRSDPDADQHYFFQ

>lcl|XM_004294357.2_prot_XP_004294405.2_1 [gene=LOC101290740] [db_xref=GeneID:101290740] [protein=beta-glucosidase 11-like] [protein_id=XP_004294405.2] [location=363..1937] [gbkey=CDS]
MLRVVLQLELLLFLCFVILLSIPLNLSLASELELGAGDDDEVKRDDFPPPPDFVFGASSSAYQVEGAADQDGRTPSILDTYTHAGKMHGATGDIACDQYHKYKEDVQLMVDTGLEAYRFSISWSR
LIPNGRGPVNPKGLQYYNNLVDQLISKGIQPHVTLHHSDLPQALDDDYGGWISPQIIKDFTAYANVCFEQFGDRVKHWTTMNEPNVFVLGGYDIGFLPPSRCSAPFGVNCSRGDSTTEPYLAIHN
FLLSHASAAALYKQTYQHKQRGFIGINVLAFWFIPRTETHKDRAAAQRAMDFYFGWVVHPLVYGEYPQVMKKNAGSRLPTFTSAESALVKGSLDFIGLNYYYTQYAKDNSAALNIQTRDYVADSA
IQISPLDHNTSTLEFPITPWGMEGLLEYIKQNYGNPPLYIHENGQQTRRNSSLEDWSRIEYLRGHIHSLLNVIRNGSNARGYFMWSLLDSIELLGGYEISYGLYYIDLDDPDLKRQPKLSAHWYS
KFLKNKNITPRSDSSDTDQLYFFQ

>lcl|XM_004294274.2_prot_XP_004294322.1_1 [gene=LOC101315216] [db_xref=GeneID:101315216] [protein=putative beta-glucosidase 41] [protein_id=XP_004294322.1] [location=136..1677] [gbkey=CDS]
MKLCIILLLITRLFTISQSLSRADFPEGFVFGTASSAYQFEGAVNEGNKGASVWDTFTKVPGKILDFSNADTAVDQYHRFQGDVDLMKDMGVNAYRFSISWPRIFPNGTGAPNSEGISYYNSLID
SLLEKGIQPFVTLYHWDLPQMLEDKYEGWLSTQIVKDFEHYAITCFQAFGDRVKNWITFNEPHGYALQSYDLGIQAPGRCSILGHLFCKKGNSSVEPYIVAHNILLSHAATYHTYQQYFKEKQGG
QIGISLDAKWHEPLSETDEDIDAAHRANEFSLGWFLDPLFFGDYPLSMKKLVGKRLPAISKEVSKFLVGSVDFIGINHYTTLYARNDRTRIRKFILQDATSDAAVITTHYKGGVAIGEKAASRWL
RIVPWGIRKLARYIKDKYGNPPMMITENGMDDPNKPFTSMEKALNDEKRIRFHRDYLSNLSAAIREDKCDVRGYFVWSLLDNWEWNMGYTVRFGLYYVDYKKNLTRIPKSSVQWFTRFLQVNNNQ
QRSQTLLNTSVQI

>lcl|XM_004293223.2_prot_XP_004293271.1_1 [gene=LOC101312701] [db_xref=GeneID:101312701] [protein=beta-glucosidase 47] [protein_id=XP_004293271.1] [location=364..1908] [gbkey=CDS]
MEVLSVSHAFLMTEIMCFSILVVSCNHISLKGNSQSNFLFGTASSSYQFEGAFLNDGKSLNNWDVFSHKPGNILDGTNGDIAVDQYHLYQEDLDLMDYIGVNSYRFSISWARILPKGRFGKVNRA
GIDHYNKLIDALLLRGIQPFVTLTHYDIPQELEDRYGAWLSPLLQEDFRYYADICFKFFGDRVKYWSTFNEPNVLAIRGYRSGIYPPSHCSLPFGNCISGDSEREPFIAAHNIILSHAAAVNVYR
TKYQKKQEGSIGIVMNAVWYEPISSSLEDKLAAERAQSFYINWFLDPIVHGRYPAEMHELLGDDLPEFSMFDMEMLKNGLDFIGINHYTSFYSKDCMISPCEPGPGVSRTEGYALRTAQKDGIYI
GEPTAVDWLYVHPQGMEKMVTYIKERYNNTPMFITENGYAEEANSNTTNEEQFNDVKRVEYMRSYLQALADTMRTKGADVRGYFVWSLLDNFEWTSGYTVQFGLHHVDNVTLKRTPRLSADWYRQ
FIVNNTLQILSAQE

>lcl|JX244263.1_prot_AFU61922.1_1 [gene=BG3] [protein=beta-glucosidase 3] [protein_id=AFU61922.1] [location=1..1563] [gbkey=CDS]
MASISGGSSFLNIIAISLIVSAFASLVRGDDLSRGQYSTAFLNRSSFPAGFVFGTASSAYQYEGAANLSGRGPSIWDTFTHKYPDKITDGSNGDVAIDSYHRYKEDVAIMKEMGLDVYRFSISWS
RVLPTGKLSGGVNKEGIEYYNNLINELLANGIKPFVTLFHWDLPQPLQDEHGGFISPHIVKHFKAYAELCYREFGDQVKHWITFNEPIALAVAGYGLGALAPGRCSAWINPNCTGGNSTTEPYLV
THYQLLAHAAAVNLYKKHYQESQKGLIGITLVAQWLVPNTTARHDRAAQLRGLDFMLGWYMDPLTNGDYPKSMKSLVGDRLPKFKKEQSKLLKGSFDFIGLNYYTSNYVSDAPQLVKVVNASFMT
DSLATMSPLRNGIPIGPKAASEDLYIYPRGIRDILLYTKRKYNNPLIYITENGVDEFNDPKLTLEEALADNQRIDYHFRHLYYLQEAIKDGVNVKGYFAWSFLDNFEWTLGYTVRFGINYVDYKD
GLKRYPKHSALWFKNFLKKY

>lcl|JX244262.1_prot_AFU61921.1_1 [gene=BG2] [protein=beta-glucosidase 2] [protein_id=AFU61921.1] [location=1..1053] [gbkey=CDS]
MGKWWNDSSGWRDGGWTRELEVPCWSHALITDVGAKNQRVRTDRGVSINHLGGTGSVKSTALPSILDFWLLYARMAKIELLKEYEQADEISRRAFPPNFVFGVATSAYQVEGACREGNRGPSIWD
AFTHTKGKIIDGSNGDIAVDQYHRYKEDVDLIAKLGFAAYRFSISWSRIFPDGLGTTVNEDGIAYYNNIINALLEKGIQPYVTLYHWDLPLYLHESMGGWLNKQIVKFFSVYADTCFANFGDRVK
DWITINEPLQTSVNGYGYGIFAPGRHEHASTEPYLVAHHQLLAHAAAVSIYRSKYKDKQGGQIGIAVDCEWAEANSDKTEDKIAAARRLDFQLGWYLLLL

>lcl|JX244261.1_prot_AFU61920.1_1 [gene=BG1] [protein=beta-glucosidase 1] [protein_id=AFU61920.1] [location=1..1848] [gbkey=CDS]
MGWKRGIALFIAFQVIIGCSSQSNSEINRGSFPKGFVFGTASSAFQYEGAVKEDGRGPSVWDIFSHTFGKITDFSNADVAVDQYHLYDARLLQPISGDVQLMKDMGMDAYRFSISWSRIFPNGTG
QINQAGVDHYNNLINSLLAKGIEPYVTLYHWDLPQALEDRYTGWLDAQIIQDFAVYAETCFQKFGDRVKHWITFNEPHTFAVQGYDVGLQAPGRCSLLGRLFCRAGNSATEPYIVAHNVILSHAT
VADIYRKKYKPKQRGSIGASFDVIWYRSATNSTADIEATERAQDFQLGWFLDPFIFGDYPFSMRSRVGSRLPKFSKSESTLIKGSLDFVGINHYTTFYASNDSSHIIGLLNDSLSDSGAIALHSI
FVLFSAFKDGKAIGDKANSIWLYIVPEGMRSLMNYIKKKYGNPPVLITENGMDDPNSPFISLKDALKDEKRISYHRDYLSNLLASIKEDGCNVNGYFAWSLLDNWEWGAGYTSRFGLYFIDYKDK
LKRYPKDSGLALSSLLPDGNSICPFSLLYVVQLEYRRRYLRFASLCSVAGKIYTLCGGLAWPWVCLQQAVMEAGGGGGGRSDHGSSSVGLGLSLIFEVVWASRWVRARHLGLNPF

Fragaria × ananassa gene homologues (referred to the *F. vesca* protein sequence)

>XM_004309432.2_bg42
ATGGCCAAGATTGAGTTGTTAAAGGAATATGAACAAGCAGATGAAATCTCTCGCAGAGCCTTCCCTCCTAACTTCGTCTTTGGCGTTGCCACCTCGGCCTATCAGGTTGAAGGTGCCTGCAGGGA
GGGCAACAGGGGTCCTAGTATCTGGGATGCTTTTACACACACTAAAGGCAAGATCATTGATGGTAGCAATGGTGACATTGCTGTGGATCAATATCACCGGTATAAGGAGGATGTTGATCTCATTG
CCAAGTTGGGATTTGCTGCTTATCGGTTTTCCATATCATGGTCTCGAATCTTTCCTGATGGTCTAGGAACCACTGTCAATGAAGATGGGATTGCTTACTACAACAATATCATTAATGCTCTCCTG
GAAAAAGGCATCCAACCTTATGTAACACTGTACCATTGGGATCTACCATTGTACCTTCATGAAAGTATGGGAGGGTGGTTAAACAAGCAGATTGTAAAATTCTTCTCAGTCTATGCGGACACCTG
CTTCGCAAATTTTGGCGATAGAGTAAAGGATTGGATCACAATTAATGAGCCTCTTCAAACTTCTGTCAATGGTTATGGTTACGGGATATTTGCTCCTGGAAGACATGAACATGCATCTACAGAAC
CATATTTGGTTGCGCATCATCAACTGTTGGCCCATGCAGCTGCTGTTTCAATATACCGAAGCAAATATAAGGACAAGCAAGGTGGACAAATAGGCATAGCAGTGGACTGTGAATGGGCAGAGGCC
AATTCAGATAAAACTGAAGACAAAATTGCTGCAGCAAGGCGCTTAGATTTTCAGCTTGGATGGTACTTAGATCCAATATATTCTGGAGAATATCCCAAAGTTATGCGTGAACGACTTGGAGATCG
GCTTCCCATATTTTCAAAGGAAGATAAGGATTTGCTCAAGAACTCATTGGACTTTGTTGGTCTAAATCACTATACATCAAGATTTATTGCTCATGTAAAAGACAGCCCTGAGGAGGGTGATTTTT
ATAAAGCGCAAGAGATGGAGAGGATTGATAAATGGGAAGGAGGTGAGATTATTGGTGAGAAGGCAGCATCAGAATGGCTTTATGTTGTTCCATGGGGCATCCATAAGGTCTTGAATTACATAGCA
CAGAGATACAATAGTCCACCAATATATATTACTGAGAATGGTATGGATGACGAGGATGATGATTCCTCCCCACTTAATGAAATGCTAGATGACAAACTGAGAGTCAGTTATTTTAAGAGATACCT
TGCAGCGGTCAGCGATGCAATGAGGGATGGAGCAGATGTGAGGGGATACTTTGCATGGTCGTTACTGGATAACTTTGAGT
GGGCACAAGGCTATACCAAGCGCTTTGGTTTGGTTTACATTGACTACAAGAATGGGCTCTCCCGGCACCCGAAATCTTCTGCGTACTGGTTCTCGCGGTTCTTGAAAAATGGTGAAGGGAGAGAT
GGGAAGGATGATTAA

>XM_004303949.2_bg13
ATGAGGATGAGGCCATCATCTTGCTCTATGGAACACTACTGTTGGTTTTGTGTTTTTATTGGTGTAGAAACCAGAGAGTACTTACCCTTATTCAACGAGTCCGGCAAAATCCAGGCTTCTGAGCA
GATCGTTAGGGGTTGCTGGTTCTGCAGATTTTATACCATGATCTCTTGTTTGACAGCAAGAACAATCGAGGCTTGTTTCCGGATATGCTGCCTCATCTTTGAGCTTGCCGGTCAATCTTCACCGC
CCGCTGGTAGAGGAGCCCTTGATGATAACGCAAAACTTGCAAACCTCAATGAAGCTCAGACTAGTACACGACAAGAAACTATTAGCAGGAAAGACTTCCCTGCTGATTTCAAATTTGGTTGCGCA
ACTTCTGCTTTGCAGACAGAAGGATGCGGAACGGAAGGAGGGAGAGGACCAGCGACATGGGATGAGTACATACAAGATGGAACTCCACCCATAAAAATTGCAGTTGATTCGTACCACCGCTATAA

GGAGGACGTGCAACTGCTCAAGGCAATGGGAGTGGATACTTACAGATTCTCCATGTCTTGGTCAAGAATTCTCCCTGAGGGAACCATAGACGGGGGAGTAAACCAGGAGGGTATCGACTTCTATA
ACAACTTCATCAACGACCTAATTGCGAATGGGATAACTCCATTTGCGACACTGTTCCACTTCGACTTGCCCACAGCACTGAACACCAAATACACCGGTTTCTTAAGCATTGACATTGTGGAGGAT
TTCAAAGCATATGCTGATTTGTGTTTCAAAACTTTTGGCGATCGAGTGAAGCACTGGGCAACTATCAATGAACCACAAGTATGGGGTCAATATGGCTTCACTAACCCAGATCCGAACGCAAACGC
TGCCACCGATCCTTTCCTCTCTACACATCATGTCTTGTTGGCTCACGCTGCTGCTGCCAAACTCTACAAAAACACATACCAGGCATCACAAGGAGGAGAGATTGGAATTCCCCTGGTTGTTGAAT
GGTTTGAGCCATTTGAAGACACCATTCCAGATAAAGCTGCGGCCCGTAGAGCATTTGATTTCTTAGTCGGATGGTTTATGGAGCCACTAGTATATGGTGATTACCCGTTCATTATGAAAACTCTA
GTAAAAGATGGACTTCCAAATTTCACAGACGAGCAGAAAGAGCTGGTAAAAGGGTCATACGATTATATTGGGGTGAACTACTATACTTCCCGATTCGCGGCTCCGATAGCAATAGTCGACGGTGA
AGAAATTACCGACTCTGATCAGTACCAGCATGTTAACATCACAGTCGATGGACCAGATGGGGAACCCATAGGCACTCCAACACCAGGGAGCAGTGAGATCTATGTTTACCCACAAGGGCTGAAAA
AGGCATTGATTTTACTGAAGGAATACGGCAATCCCAAATTTTACATTACTGAGAATGGGTATCCAGACAAACGAGATGACACCATTCCAATTTCAACTGCTTTGATCGACGATGCTCGAATTCAT
CATATAAAGGATCACCTCACCGCCATTAAAGAGGCCATGAACACCGGAACAAATGTGAAGGCTTACCTGATGTGGGCAATGTTGGACTGCGTGGAGATGGGATCCGGTTATCAAGTTCGATTTGG
TCTCAATTTTACAAATTACCTTGGCAACTTGAATCGAACTGCCAAGAAATCCGCCGGCTGGCTCAAAACTTTCTTGACTTCCTGA

>XM_004303947.2_bg24
ATGGCTCTCTCTCTAAGCTTTTCGCTGACAACGTCGGCAACAACAGCGCCTTTGAGCTCCTTCAAGAGACTACCGAGCCGTATTCCTACGGTGAAAGCCCTAGGAGTGTTCCCATCTAAGGGAAT
AGATCATAAGAGGGCCATCAGAATTCGACCATGCTCTTCATTGGGAGATCTTACTTCAGCACTCACAAGTGCAGTTACTACTACTGTGGCCGTCGCTGTTGACACTGCGGCCGCGGTTGTTGATA
CCACAACCACAACCACAACCACGACCGAAACTGCAGAAGTAACCAGGGCTGATTTTCCTGCTGATTTCAAGTTTGGTTGCGCCACTTCTGCTTTCCAGACAGAAGGATCTGGAACCGAAGGAGGG
AGAGGACCGGCCACATGGGATGATTACATACAAGATACCACAGTAGATGGCACAGAAATAGCAGTTGATTCTTACAACCGCTACAAGGACGATGTGCAACTGCTGAAGAATATGGGAGTGGATAC
ATACAGATTCTCCATGTCATGGTCAAGACTATTGCCTGACGGAACCATAGACGGTGGAATAAACCAGGAGGGTATCGACTTCTACAACAACTTCATCGACGAACTGATCGCAAATGGGATAACTC
CATTTGTGACACTGTTCCACTTCGACTTGCCCACAGCACTGAAGGACAAGTACAACGGTTTCTTGAGCATTGACATTGTGGAGGATTTCAAAGCATATGCTGATTTGTGTTTCAAAACTTTTGGC
GATCGAGTGAAGTACTGGGCTACTATCAATGAGCCTCAAGTATGGGGGCAATACGGCTTCACTAACACTGCTGCGGATGCAAACGCTGCCACTGATCCTTATGTCTCTTCGCATAATGTCATATT
AGCCCATGCTACAGCTGCCAAGCTCTACAAGAACACATACCAGTCAACGCAAGGAGGAGAGATCGGAATCCCGATCGTGGTTGAATGGTTTGAGCCATTCGAGGACACCCCGCAAGATGTCTTTG
CAGCCAAAAGAGCATTCGACTTTTTGACAGGATGGTTTCTGGAGCCCCTAGTGTATGGTGATTACCCATTTGTTATGAAGGCTTTGGTGAGGGATGGCCTTCCAGAGTTCACAGATGAGCAGAAA
GAGTTGGTAAAGGGCTCATATGATTACATTGGGGTGAACTACTACACTTCCAGATTCGCCACTTCTGTACCAATAACTAGCAACGATGTCTACACAACTATGGATCAGTACCAGCATGCTACCAT
CACAGTGGAAGGATCAGACGGGGAGCCCATAGGCGATGCAACACCAGGGAGCAGTTCGATCTACGTCTATCCAGAAGGATTAAGAAAGGGATTGACATTACTGAAGGAATACAACAATCCCAAAA
TCTACGTTACTGAGAATGGATATCCGGACGCAAGAGATGACACCCTTTCAGTGGAAGAAGCTTCGGTTGATGATGCTCGAATTCAGCACATCAAGGATCATCTCGCCGCTGTCAAAGCCGCCAGA
GATGCGGGAGCAGATGTGCAGGGTTACCTGATGTGGGCACTGATGGACTGCGTGGAGATGGGATCTGGCTACGCGGTTCGGTTTGGCCTCAACTACACTGATTACCTCAATGACTTGGTCCGAAC
TCCAAAGAAGTCCGCCGCCTGGCTCAAAGATTTCTTGGCATCAACCACTTCCACTTGA

>XM_011468950.1_bg24_x4
ATGTCAACGACAATTGATTCATACAAGCGATACAAGCAAGATGTGAAGGCAGTGAAAGCCCTTGGAGTTGATTCTTATAGATTTTCCATATCTTGGACCAGGATTCTCCCAAAGGGAACCTTGAG
TGGAGGAATAAACCAAGAGGGTGTCGATCACTACAACAACTTGATAGATGAACTAATTAAGTATGGCATCACACCCTATGTAACCATATTACATTTTGATCCACCACAAGCCTTGACAGTCAAGT
ATGGAGGATTTCTCAGTCGCTCCTTTGTGGATGATTTTAAGGATTACAGTGAACTTTGCTTTAAAATCTTTGGAGATAGAGTAAAAAATTGGATTACAATCAACGAGCCTTTCATTATAGCCAAA
ATGGGGTATGATCTTGGGGTTGCTGCACCAGGCAGGTGTTCCATACCAGGCGGCGATTTTGATTATTGCACAGCTGGAAATTCATCTACAGAACCTTACATTGTGGCTCATAACCTTCTCCTTGC
CCATGCCACTGTTGTTAAGCTCTATAGAGAGAAGTTTCAGACAAAACAAGGTGGACAAATTGGATGTAGCCATGTAGGTACATATATTGAGGCTTATTCACATTCAGCAAAGGACAAGGCTGCAG
CTAAAAGAATATTGGACTTTGAACTTGGATGGTTCATGGAACCAATAGTATATGGATGTTATCCAAAGAGTATGAGGCGCTTGGTCAAGAAAAGGCTCCCCAGTTTTACCAAAGAAGAGAAGAAA
TTGATTATGGGATCCTTCGATTTCATTGGCATCAACTATTATACCTCCAGATATGGTAAAAACATTCCAGCACGTCCACATATGCCACACTTCTCTCGCAATGATCCTTTAGCCTCATCAGAGTT
CAAAAATGTAAATGGAGTCCCAATTGGTCCTCAGGCTGATGGCAGCACGTTCATCTTCTCTTATCCACAAGGTCTCAAGAAACTTTTGAAGTTCATGAAGCAAAAATACCAGAGTCCTAAGATCT
ACATTACTGAAAATGGAATTACAGTAGCAAAGAACAATAGGCGTGGACTCGATGTAGCACTGAAGGATCCACACAGAATTGAATGCATTCTTCGGCATCTGTACATGATCAAGCAGGCCATAAAG
AAGGGTGTGAATGTCGTAGGATACTTCCACTGGGCTCTATTTGATGACTTTGAGTGGGGAGAAGGCTATACCCCAAGGTTCGGGCTCTACTATGTCGACTACAAAGACAATCTTAAGCGCATTCC
AAAAGAATCTGCTAAGTGGCTCCCTAAATTTCTAAATGGTTAG

>XM_011468949.1_bg24_x3
ATGTCAACGACAATTGATTCATACAAGCGATACAAGCAAGATGTGAAGGCAGTGAAAGCCCTTGGAGTTGATTCTTATAGATTTTCCATATCTTGGACCAGGATTCTCCCAAAGGGAACCTTGAG
TGGAGGAATAAACCAAGAGGGTGTCGATCACTACAACAACTTGATAGATGAACTAATTAAGTATGGCATCACACCCTATGTAACCATATTACATTTTGATCCACCACAAGCCTTGACAGTCAAGT
ATGGAGGATTTCTCAGTCGCTCCTTTGTGGATGATTTTAAGGATTACAGTGAACTTTGCTTTAAAATCTTTGGAGATAGAGTAAAAAATTGGATTACAATCAACGAGCCTTTCATTATAGCCAAA
ATGGGGTATGATCTTGGGGTTGCTGCACCAGGCAGGTGTTCCATACCAGGCGGCGATTTTGATTATTGCACAGCTGGAAATTCATCTACAGAACCTTACATTGTGGCTCATAACCTTCTCCTTGC
CCATGCCACTGTTGTTAAGCTCTATAGAGAGAAGTTTCAGACAAAACAAGGTGGACAAATTGGATGTAGCCATGTAGGTACATATATTGAGGCTTATTCACATTCAGCAAAGGACAAGGCTGCAG
CTAAAAGAATATTGGACTTTGAACTTGGATGGTTCATGGAACCAATAGTATATGGATGTTATCCAAAGAGTATGAGGCGCTTGGTCAAGAAAAGGCTCCCCAGTTTTACCAAAGAAGAGAAGAAA
TTGATTATGGGATCCTTCGATTTCATTGGCATCAACTATTATACCTCCAGATATGGTAAAAACATTCCAGCACGTCCACATATGCCACACTTCTCTCGCAATGATCCTTTAGCCTCATCAGAGTT
CAAAAATGTAAATGGAGTCCCAATTGGTCCTCAGGCTGATGGCAGCACGTTCATCTTCTCTTATCCACAAGGTCTCAAGAAACTTTTGAAGTTCATGAAGCAAAAATACCAGAGTCCTAAGATCT
ACATTACTGAAAATGGAATTACAGTAGCAAAGAACAATAGGCGTGGACTCGATGTAGCACTGAAGGATCCACACAGAATTGAATGCATTCTTCGGCATCTGTACATGATCAAGCAGGCCATAAAG
AAGGGTGTGAATGTCGTAGGATACTTCCACTGGGCTCTATTTGATGACTTTGAGTGGGGAGAAGGCTATACCCCAAGGTTCGGGCTCTACTATGTCGACTACAAAGACAATCTTAAGCGCATTCC
AAAAGAATCTGCTAAGTGGCTCCCTAAATTTCTAAATGGTTAG

>XM_011468949.1_bg24_x3
ATGTCAACGACAATTGATTCATACAAGCGATACAAGCAAGATGTGAAGGCAGTGAAAGCCCTTGGAGTTGATTCTTATAGATTTTCCATATCTTGGACCAGGATTCTCCCAAAGGGAACCTTGAG
TGGAGGAATAAACCAAGAGGGTGTCGATCACTACAACAACTTGATAGATGAACTAATTAAGTATGGCATCACACCCTATGTAACCATATTACATTTTGATCCACCACAAGCCTTGACAGTCAAGT
ATGGAGGATTTCTCAGTCGCTCCTTTGTGGATGATTTTAAGGATTACAGTGAACTTTGCTTTAAAATCTTTGGAGATAGAGTAAAAAATTGGATTACAATCAACGAGCCTTTCATTATAGCCAAA
ATGGGGTATGATCTTGGGGTTGCTGCACCAGGCAGGTGTTCCATACCAGGCGGCGATTTTGATTATTGCACAGCTGGAAATTCATCTACAGAACCTTACATTGTGGCTCATAACCTTCTCCTTGC
CCATGCCACTGTTGTTAAGCTCTATAGAGAGAAGTTTCAGACAAAACAAGGTGGACAAATTGGATGTAGCCATGTAGGTACATATATTGAGGCTTATTCACATTCAGCAAAGGACAAGGCTGCAG
CTAAAAGAATATTGGACTTTGAACTTGGATGGTTCATGGAACCAATAGTATATGGATGTTATCCAAAGAGTATGAGGCGCTTGGTCAAGAAAAGGCTCCCCAGTTTTACCAAAGAAGAGAAGAAA
TTGATTATGGGATCCTTCGATTTCATTGGCATCAACTATTATACCTCCAGATATGGTAAAAACATTCCAGCACGTCCACATATGCCACACTTCTCTCGCAATGATCCTTTAGCCTCATCAGAGTT
CAAAAATGTAAATGGAGTCCCAATTGGTCCTCAGGCTGATGGCAGCACGTTCATCTTCTCTTATCCACAAGGTCTCAAGAAACTTTTGAAGTTCATGAAGCAAAAATACCAGAGTCCTAAGATCT
ACATTACTGAAAATGGAATTACAGTAGCAAAGAACAATAGGCGTGGACTCGATGTAGCACTGAAGGATCCACACAGAATTGAATGCATTCTTCGGCATCTGTACATGATCAAGCAGGCCATAAAG
AAGGGTGTGAATGTCGTAGGATACTTCCACTGGGCTCTATTTGATGACTTTGAGTGGGGAGAAGGCTATACCCCAAGGTTCGGGCTCTACTATGTCGACTACAAAGACAATCTTAAGCGCATTCC
AAAAGAATCTGCTAAGTGGCTCCCTAAATTTCTAAATGGTTAG

>XM_011468948.1_bg24_x2
ATGTCAACGACAATTGATTCATACAAGCGATACAAGCAAGATGTGAAGGCAGTGAAAGCCCTTGGAGTTGATTCTTATAGATTTTCCATATCTTGGACCAGGATTCTCCCAAAGGGAACCTTGAG
TGGAGGAATAAACCAAGAGGGTGTCGATCACTACAACAACTTGATAGATGAACTAATTAAGTATGGCATCACACCCTATGTAACCATATTACATTTTGATCCACCACAAGCCTTGACAGTCAAGT
ATGGAGGATTTCTCAGTCGCTCCTTTGTGGATGATTTTAAGGATTACAGTGAACTTTGCTTTAAAATCTTTGGAGATAGAGTAAAAAATTGGATTACAATCAACGAGCCTTTCATTATAGCCAAA
ATGGGGTATGATCTTGGGGTTGCTGCACCAGGCAGGTGTTCCATACCAGGCGGCGATTTTGATTATTGCACAGCTGGAAATTCATCTACAGAACCTTACATTGTGGCTCATAACCTTCTCCTTGC
CCATGCCACTGTTGTTAAGCTCTATAGAGAGAAGTTTCAGACAAAACAAGGTGGACAAATTGGATGTAGCCATGTAGGTACATATATTGAGGCTTATTCACATTCAGCAAAGGACAAGGCTGCAG
CTAAAAGAATATTGGACTTTGAACTTGGATGGTTCATGGAACCAATAGTATATGGATGTTATCCAAAGAGTATGAGGCGCTTGGTCAAGAAAAGGCTCCCCAGTTTTACCAAAGAAGAGAAGAAA
TTGATTATGGGATCCTTCGATTTCATTGGCATCAACTATTATACCTCCAGATATGGTAAAAACATTCCAGCACGTCCACATATGCCACACTTCTCTCGCAATGATCCTTTAGCCTCATCAGAGTT
CAAAAATGTAAATGGAGTCCCAATTGGTCCTCAGGCTGATGGCAGCACGTTCATCTTCTCTTATCCACAAGGTCTCAAGAAACTTTTGAAGTTCATGAAGCAAAAATACCAGAGTCCTAAGATCT
ACATTACTGAAAATGGAATTACAGTAGCAAAGAACAATAGGCGTGGACTCGATGTAGCACTGAAGGATCCACACAGAATTGAATGCATTCTTCGGCATCTGTACATGATCAAGCAGGCCATAAAG
AAGGGTGTGAATGTCGTAGGATACTTCCACTGGGCTCTATTTGATGACTTTGAGTGGGGAGAAGGCTATACCCCAAGGTTCGGGCTCTACTATGTCGACTACAAAGACAATCTTAAGCGCATTCC
AAAAGAATCTGCTAAGTGGCTCCCTAAATTTCTAAATGGTTAG

>XM_011468947.1_bg24_x1
ATGGAGTGTATAGCGAATGTTACAGTGAATGCTGAGAAGAAGCTCCAGGCATTGCACATCAATGCATCAAACCCTAAAGAATTGAAAGTGAAGTGGTCCGACTTCCCCAGTGATTTTGTATTTGG
AGTCAGCACTGCTGCTGCACAGATTGAAGGAACAACAAATAAAGCAGGACGAGGACCAAGTGTTTGGGACGAGTTTGTTAAGACATTTCCAGAAAGAATTGCAGACCATTCCACGATGTCAACGA
CAATTGATTCATACAAGCGATACAAGCAAGATGTGAAGGCAGTGAAAGCCCTTGGAGTTGATTCTTATAGATTTTCCATATCTTGGACCAGGATTCTCCCAAAGGGAACCTTGAGTGGAGGAATA
AACCAAGAGGGTGTCGATCACTACAACAACTTGATAGATGAACTAATTAAGTATGGCATCACACCCTATGTAACCATATTACATTTTGATCCACCACAAGCCTTGACAGTCAAGTATGGAGGATT
TCTCAGTCGCTCCTTTGTGGATGATTTTAAGGATTACAGTGAACTTTGCTTTAAAATCTTTGGAGATAGAGTAAAAAATTGGATTACAATCAACGAGCCTTTCATTATAGCCAAAATGGGGTATG
ATCTTGGGGTTGCTGCACCAGGCAGGTGTTCCATACCAGGCGGCGATTTTGATTATTGCACAGCTGGAAATTCATCTACAGAACCTTACATTGTGGCTCATAACCTTCTCCTTGCCCATGCCACT
GTTGTTAAGCTCTATAGAGAGAAGTTTCAGACAAAACAAGGTGGACAAATTGGATGTAGCCATGTAGGTACATATATTGAGGCTTATTCACATTCAGCAAAGGACAAGGCTGCAGCTAAAAGAAT
ATTGGACTTTGAACTTGGATGGTTCATGGAACCAATAGTATATGGATGTTATCCAAAGAGTATGAGGCGCTTGGTCAAGAAAAGGCTCCCCAGTTTTACCAAAGAAGAGAAGAAATTGATTATGG
GATCCTTCGATTTCATTGGCATCAACTATTATACCTCCAGATATGGTAAAAACATTCCAGCACGTCCACATATGCCACACTTCTCTCGCAATGATCCTTTAGCCTCATCAGAGTTCAAAAATGTA
AATGGAGTCCCAATTGGTCCTCAGGCTGATGGCAGCACGTTCATCTTCTCTTATCCACAAGGTCTCAAGAAACTTTTGAAGTTCATGAAGCAAAAATACCAGAGTCCTAAGATCTACATTACTGA
AAATGGAATTACAGTAGCAAAGAACAATAGGCGTGGACTCGATGTAGCACTGAAGGATCCACACAGAATTGAATGCATTCTTCGGCATCTGTACATGATCAAGCAGGCCATAAAGAAGGGTGTGA
ATGTCGTAGGATACTTCCACTGGGCTCTATTTGATGACTTTGAGTGGGGAGAAGGCTATACCCCAAGGTTCGGGCTCTACTATGTCGACTACAAAGACAATCTTAAGCGCATTCCAAAAGAATCT
GCTAAGTGGCTCCCTAAATTTCTAAATGGTTAG

>XM_004299497.2_bg44
ATGAGAACCACACCAGTGCTGGTGCTCTTCTGGGTCAGTCTTGTTGTTGTGATCCAGTACGTTGCGGGTGCAGCCAAGCACAATGGAGCTCCTTTCCCTGAGGCTGAGGGTGGACTGAGAGCTAG
TTTCCCAAAGGGGTTTGTGTTCGGAACGGCGACGTCTGCTTATCAGGTGGAAGGCATGGCCAACAAGGAAGGTCGTGGACCCAGCATTTGGGATACCTTTGTTAAAATCCCAGGGATTGTTGCCA
ATAATGCTACTGGAGATGTTACAGTTGACCAATATCACCGTTACAAAGAAGATATTGATCTCCTGGCGAATCTTAATTTTGATGCCTACCGGTTTTCAATATCATGGTCCAGAATATTTCCAGAG
GGTACTGGGAAAGTGAATTGGAAAGGAGTGGCATACTACAATCGGTTAATTGACTACTTGCTTAAAAGAGGCATTACTCCCTATGCAAATTTATACCACTATGATCTTCCTCTTGCACTTGAGAA
GAAGTACCTGGGATTGTTAAGTGACAGAGTTGTGAAAGATTTTGCCGATTTTGCCGATTTTTGCTTCAAGACATATGGAGACAGAGTGAAGAACTGGATGACATTCAATGAGCCTAGAGTTGTGG
CTGCTCTTGGCTATGACACTGGATTCTTCGCTCCGGGACGGTGCTCCAAAGCTTATGGAAATTGTACAGCTGGGAATTCAGGAACTGAGCCTTACATTGCTGCACATCATTTGATTTTGTCACAT
GCAGCAGCAGTTCAGAGATACCGCGAAAAGTATCAGAAAACACAAAAAGGAAAGATTGGTATACTGTTGGATTTTGTTTGGTATGAGCCTCTTACCAGATCAAAGGCTGACAGATATGCAGCTCA
AAGGGCAAGAGACTTTCATGTTGGATGGTTCATTCATCCGATTGTATATGGGGAGTATCCGAAAACAATCCAAAATATTGTTGGCAATAGGCTACCTAAGTTTACGAAAGAGGAGGTTAAGATGG
TGAAGGGTTCAATGGACTTTGTAGGCATCAACGAGTACACTGCTTACTATATATATGCTCCAGATAAAAATCAAACTAAATCAAACGCCTTGGGATACCAGAATGACTGGAATGCTGGATTTGCA
TATGAGAAAAATGGAGTGCCTATTGGTCCTAGGGCATATTCTTCTTGGCTCTACCAAGTACCATGGGGTATGTACAAATGTATAACATACATAAAAGAGCATTATGGAACCCGCCTGTGATTTT
ATCCGAAAATGGCATGGATAACCCCGGAAACCAGACGCTTCCAAAGGCATTGCATGACACCAATAGGATCCACTACTACAGAAGCTATCTGGAGCAACTGAAGAAGGCAGTCGATGAGGGAGCCA
ATGTGGTAGGCTACTTTGCTTGGTCATTGCTGGACAACTTCGAGTGGAGATTGGGTTACACTTCAAGGTTTGGCATAGTCTATGTTGACTTCACCACCCTCAAGAGGTACCCAAAGATGTCTGCC
TACTGGTTCAAGAAACTTCTCACCAAAAAGAAGCACTAG

>XM_004299498.2_bg44
ATGAGAACCGCGGTACTACTGCTGGTCTTGTTGCTCAGCGGTTTTGTTGTTGTGATCCAGTACGCTGCGGGTGCAGCTAAGTACAATGGAGCTCCTTTCCCTGAGGCCGAGGGTGGACTCAGAAC
TGGTTTTCCGAAAGGGTTTGTGTTCGGAACGGCGACATCTGCTTATCAGGTGGAAGGCATGGCCAACAAGGAAGGTCGTGGACCTAGCATTTGGGATGCCTTCGTTAAAATTCCAGGGAATATTG
CAAATAATGATACAGCAGATGTTACAGCGGACGAATATCACCGTTACAAAGAAGACATTGATATCATGCAGGACCTAAATTTTGATGTCTATAGGTTCTCAATATCATGGTCAAGAATCTTCCCA
GATGGAACTGGGAAAGTGAATTTGAAAGGAGTTGCATACTACAACCGGTTAATCAATTACTTGCTCGAAAGAGGCATTACCCCTTATGCAAATTTGCATCACTATGATCTTCCCCTAGCACTTGA
GCAAGAGTACCTAGGGTTCTTAAGTGACAGAGTGGTGAAGGATTTCGCGGATTACGCGGAATTTTGCTTCAAGACATATGGAGACAGAGTCAAGTACTGGACGACATTCAATGAGCCTAGAGTGG
TGGCTGCTCTTGGCTATGACAATGCACTGAATCCTCCGGCAAGGTGCTCCAAAGCTTATGGAAATTGCACAGTTGGGAATTCAGGAACTGAACCATACATTGTTGCTCATCATTTGCTTTTGTCA
CATGCAGCTGCGGTTCATAGATACCGCGAAAAGTATCAGAAAACACAAAAAGGAAGTGTTGGTATTCTCTTGGACTTTAACTGGTATGAGCCTCTTACCAGTTCAAAGGATGACAGAGATGCAGC
TCAAAGGGCAAGAGACTTTCATATTGGATGGTTCATTCATCCGATTGTATATGGGGAGTATCCAAAAACAATGCAACATATTGTTGGCGATAGGCTACCAAAGTTTACTAAAGAGGAGGTTAAGA
TGGTGAAGGGCTCAATAGATTTTGTAGGCATCAACCAGTATACTGCTTATTACATGTATGATCCACATCAAAACAAAACAAAACCAAATGCCTTGGGATACCAAAATGACTGGAATGCCGGATTT
GCTTTTGAGAAGAATGGAGTGCTTATCGGTCCTCAGGCATATTCTTCTTGGCTCTACCAAGTCCCATGGGGTTTGTACAAGTGTGTAACATACATAAAAAAGAACTATGGAACCCAACCGTGAT
TATATCCGAAAACGGCATGGATAACCCGGGAAACCAGACGCTTCAAGAGGCATTGCACGACACCAACAGGATCGACTTCTACAGAAGCTATCTGGAGCAACTGAAGAAGGCAGTTGATGAAGGCG
CCAATGTGGTAGGCTATTTTGCCTGGTCATTGCTGGACAACTTCGAATGGAGATTGGGTTACACTTCAAGGTTTGGCATAGTCTATGTTGACTTCACCACCCTAAAGAGATATCCAAAGATGTCT
GCCTACTGGTTCAAGAAACTGCTTACCAAAAAGAAGCATTAA

>XM_011465402.1_bg13
ATGGCTTCATCAACCTATGCTCTCATCGCGCTCTTTGCGATGGTTTCGATAGCTGGCTGGCCTGTTCAGGCAACATGGGACGGCGTGGTTCCAGTTGAGGCGAATTTGACAGTAGGCGGATACAC
TTTCAGATTGAATTTAAACAGTCCTGCATATCTTGAAGAATTGAACGTAACGAAATCAGATTTTCCAACTGATTTCGCTTTTGGAGTTGGTTCCTCTGCTGCACAGATTGAAGGGGCAGCCAGAG
AATTTGGAAGAGGGCCAAGTACATGGGATCATCGTGTTGATATTCTTCCAGGTAGTATTCACGACAGCGACAAATTGTCCACAGCAATTGATTCATATAGACGATACAAGCAAGATATTGAGCTT
ATTAAAGACCTCGGAGTAGATTCTTACCGATTCTCCATCTCCTGGAGTAGGATTTTACCGTATGGAAGCTTAAGTGGCGGAATTAACCAACAGGGTGTTGATCACTATAACAGTTTGATCGACGA
ATTGATCAAAAATGGTATCAAACCTTACGTGACGATTTTTCACTTTGACTTACCACAAGCCCTAGAAGAGAAGTATGGAGGTTTCTTGAATCGCTCTTTTGTGGATGATTTTAGAGACTACAGTG
AACTTTGCTTCAAATTATTTGGAGACAGAGTTAAGAATTGGTTCACAATCAATGAGCCATCAGTTTCTGCCGTATACGGGTATGAACTGGGAATAGCTCCACCCGGAAAGTGTTCACTTCAGGAA
GGAGTATGTGTGTTTGGGACTTCAGATAACTGTTTAGTCCCGGCAGGTCCATGCAATTTCGGTGGCAATTCATCCACAGATCCTTACATTGCAGCACACAACCAAATTCTTGCTCATGCCACAGC
AGCAAAGCTTTACAAAGAAAAATACCAATCAACTCACAAGGGTGAGATTGGAATCGTTCTTGTTTCTAGACACTATATGCCATTTACGAAGAAGCAGGAAGATATAGCTGCAGCAAAACGACGTT
TCGACTTCACCCTAGGATGGTTTATGGATCCATTTGTGTATGGAGATTATCCGAAGAGTATGAAGGAGTTAGTGAAAGAAAGACTGCCTACATTTTCTCCAGAAGAGCAAAGTTTGATCAAGGGA
AGTCTTGATTTTGTTGGAATCAATTACTACAGTTCAACTTATGCTAGAAACAAGGCACCATCTAAAGAATTGCGCTATAGTTATGATGTATCAGCCGACGAAATAGGGCATTGGCCAGAAGGCTT
GGAGAAAGTGTTGGTGCACATGAAGAAAAAATATCAAAACCCGAAAATCTACATAACAGAAAATGGACTTAGTACGACTAGAAATGATGAGCTTCCATTTGCGGATCAACTGAAAGATCCGGAAA
GAATTGCATACATTGTTCGTCATTTGTATCGTATCAACAAGGCAATCAAGAAGGGAGTAAACGTGAAAGGCTACTTCTTCTGGGCAATGTTTGACGACTTTGAATGGGGTATGGGGTTTTTGGAT
AGATTTGGGTTATACCATATTGATTTTGCTCACAATTACAGGCGCACACCCAAGCATTCGGCCAAGTGGTTCAAAGCTTTCCTTCAAAGCAAAGTGGCTCGAGCTAGCTAA

>XM_011464992.1_bg13_x3
ATGGCTTCATCAACCAAGTTTTCCGGTGTTGTTATCGTTGCGGTTTCTGCCATCATGGCTTCGATTGCCGGCTGTTATGCCCGGGCAACTATTCCAGATTTTAATGGTGGTGTGGCAGGAAAATT
GAAATTATACAGTAGTAGTGCATTTCTCGACGAATTGGATGTTTCAAGATCAGACTTCCCAGCTGATTTTACGTTTGGAGTTGCTACCTCTGCCGCACAGACTGAAGGTTCAGCCAGAGAAGGAG
GGAGAGGACCAAGTACATGGGATCATCGCGTTGATATACTGCCAGATGCAATAATAAACAGCGACAAATTTTCTACAGCAATTGATTCATATAGACGATACAAGGAAGATATTAAGATTATCAAG
GACCTCGGAGTAAATGCTTACCGATTCTCCATCTCCTGGAGTAGAATATTACCAAAGGGAAGCTTAAGTGGGGGAATCAACCAAGAGGGTATTGATCACTATAACAGCTTCATTGACGATTTGAT
CAGAAATGGAATCGAACCTTTCGTGACGATTTTACACTCCGACCTTCCACAAGCTCTGGAAGATAAGTATGGTGGTTATTTGAACCACTCATTTGTGAATGACTTTAAAGACTATAGTGAAATTT
GCTTCAAATCATTTGGAGATAGAGTTAAAAATTGGTTCACGATTAATGAGCCGACAGTGTCTGCAGTTTACGGCTATGAACTTGGAATCGCCCCGCCGGGAAAGTGTTCACTCAAGGAAGGAGTA
TGTGTGTTTGGGACTCCAGACAACTGTTTAGTTCCGGCAGGACCATGCAATTTTGGTGGTGACTCTAGCACAGAACCTTACATTGTAGCTCACAACCAAATTCTTGGTCATGCAACCGTGGCTAA
GCTCTACAAAGAAAAATATAAAGCACAACAAAAGGGTGAGATTGGAATCGTCCTTGCTTCAAAATACTATGTGCCTTTCTCCAATTCACCTGAAGATAAAGTCGCGGCAGAACGGCTTTTCGACT
TCAACCTAGGATGGTTTATGGAACCATTTGTATACGGAGATTATCCAAAGAGTATGAGAGAGTTGGTGAAGGAAAGACTGCCTAGTTTTTCTGCACAAGAGCAAAGCTTGATCAAGGGATGTCTA
GATTTTGTTGGGATTAATTACTACACATCAAGTTATGCTAGACATAAGACACCACCACCATTAGAACAATTTCGCTATTCTTTTGATATATCTGCCGAAGAAACAGGACCATGA

>XM_011464991.1_bg13_x2
ATGGCTTCATCAACCAAGTTTTCCGGTGTTGTTATCGTTGCGGTTTCTGCCATCATGGCTTCGATTGCCGGCTGTTATGCCCGGGCAACTATTCCAGATTTTAATGGTGGTGTGGCAGGAAAATT
GAAATTATACAGTAGTAGTGCATTTCTCGACGAATTGGATGTTTCAAGATCAGACTTCCCAGCTGATTTTACGTTTGGAGTTGCTACCTCTGCCGCACAGACTGAAGGTTCAGCCAGAGAAGGAG
GGAGAGGACCAAGTACATGGGATCATCGCGTTGATATACTGCCAGATGCAATAATAAACAGCGACAAATTTTCTACAGCAATTGATTCATATAGACGATACAAGGAAGATATTAAGATTATCAAG
GACCTCGGAGTAAATGCTTACCGATTCTCCATCTCCTGGAGTAGAATATTACCAAAGGGAAGCTTAAGTGGGGGAATCAACCAAGAGGGTATTGATCACTATAACAGCTTCATTGACGATTTGAT
CAGAAATGGAATCGAACCTTTCGTGACGATTTTACACTCCGACCTTCCACAAGCTCTGGAAGATAAGTATGGTGGTTATTTGAACCACTCATTTGTGAATGACTTTAAAGACTATAGTGAAATTT
GCTTCAAATCATTTGGAGATAGAGTTAAAAATTGGTTCACGATTAATGAGCCGACAGTGTCTGCAGTTTACGGCTATGAACTTGGAATCGCCCCGCCGGGAAAGTGTTCACTCAAGGAAGGAGTA
TGTGTGTTTGGGACTCCAGACAACTGTTTAGTTCCGGCAGGACCATGCAATTTTGGTGGTGACTCTAGCACAGAACCTTACATTGTAGCTCACAACCAAATTCTTGGTCATGCAACCGTGGCTAA
GCTCTACAAAGAAAAATATAAAGCACAACAAAAGGGTGAGATTGGAATCGTCCTTGCTTCAAAATACTATGTGCCTTTCTCCAATTCACCTGAAGATAAAGTCGCGGCAGAACGGCTTTTCGACT
TCAACCTAGGATGGTTTATGGAACCATTTGTATACGGAGATTATCCAAAGAGTATGAGAGAGTTGGTGAAGGAAAGACTGCCTAGTTTTTCTGCACAAGAGCAAAGCTTGATCAAGGGATGTCTA
GATTTTGTTGGGATTAATTACTACACATCAAGTTATGCTAGACATAAGACACCACCACCATTAGAACAATTTCGCTATTCTTTTGATATATCTGCCGAAGAAACAGGTATGAAGGATGGAGATTT
CCTAGGCATGTTACATTGCAGGACCATGAAGGGCACTGGCCAGAAGGGCTGCAGAAACTGA

>XM_011464990.1_bg13_x1
ATGGCTTCATCAACCAAGTTTTCCGGTGTTGTTATCGTTGCGGTTTCTGCCATCATGGCTTCGATTGCCGGCTGTTATGCCCGGGCAACTATTCCAGATTTTAATGGTGGTGTGGCAGGAAAATT
GAAATTATACAGTAGTAGTGCATTTCTCGACGAATTGGATGTTTCAAGATCAGACTTCCCAGCTGATTTTACGTTTGGAGTTGCTACCTCTGCCGCACAGACTGAAGGTTCAGCCAGAGAAGGAG
GGAGAGGACCAAGTACATGGGATCATCGCGTTGATATACTGCCAGATGCAATAATAAACAGCGACAAATTTTCTACAGCAATTGATTCATATAGACGATACAAGGAAGATATTAAGATTATCAAG
GACCTCGGAGTAAATGCTTACCGATTCTCCATCTCCTGGAGTAGAATATTACCAAAGGGAAGCTTAAGTGGGGGAATCAACCAAGAGGGTATTGATCACTATAACAGCTTCATTGACGATTTGAT
CAGAAATGGAATCGAACCTTTCGTGACGATTTTACACTCCGACCTTCCACAAGCTCTGGAAGATAAGTATGGTGGTTATTTGAACCACTCATTTGTGAATGACTTTAAAGACTATAGTGAAATTT
GCTTCAAATCATTTGGAGATAGAGTTAAAAATTGGTTCACGATTAATGAGCCGACAGTGTCTGCAGTTTACGGCTATGAACTTGGAATCGCCCCGCCGGGAAAGTGTTCACTCAAGGAAGGAGTA
TGTGTGTTTGGGACTCCAGACAACTGTTTAGTTCCGGCAGGACCATGCAATTTTGGTGGTGACTCTAGCACAGAACCTTACATTGTAGCTCACAACCAAATTCTTGGTCATGCAACCGTGGCTAA
GCTCTACAAAGAAAAATATAAAGCACAACAAAAGGGTGAGATTGGAATCGTCCTTGCTTCAAAATACTATGTGCCTTTCTCCAATTCACCTGAAGATAAAGTCGCGGCAGAACGGCTTTTCGACT
TCAACCTAGGATGGTTTATGGAACCATTTGTATACGGAGATTATCCAAAGAGTATGAGAGAGTTGGTGAAGGAAAGACTGCCTAGTTTTTCTGCACAAGAGCAAAGCTTGATCAAGGGATGTCTA
GATTTTGTTGGGATTAATTACTACACATCAAGTTATGCTAGACATAAGACACCACCACCATTAGAACAATTTCGCTATTCTTTTGATATATCTGCCGAAGAAACAGGTATGAAGGATGGAGATTT
CCTAGGCATGGACCATGAAGGGCACTGGCCAGAAGGGCTGCAGAAACTGATGGAGTATATGATGGAAAAATACCAAAATCCAAAAATCTATATTTCTGAAAATGGACTTTACACAACACGAAACG
ATGAACTTTCGCTTGTTGATCAACTAAATGACACAAATCGAATTTCATTCCTTGTTCGACATTTGTATCGTCTCAACAAGGCGATTAAGAATGGAGTGAATGTGAAAGGATACTTCTACTGGTCT
CTCTTCGATGATTTCGAATGGGGAATGGGGTTTTTGCATAGATATGGACTATACCATATCGATTTCGATCACAATAACAGGCGCATCCCCAAACAGTCTGCTGACTGGTTCAAAGCTTTCCTTCA
AAGTGACCAAGTGACTCGAGCTAGCAGCTAA

>XM_004297206.2_bg40
ATGGGGTGGAAGAGAGGCATTGCTTTGTTTATTGCTTTTCAGGTGATTATTGGGTGTTCATCACAGTCTAATTCAGAGATTAACAGAGGCAGCTTTCCAAAGGGCTTTGTTTTTGGGACTGCTTC
TTCTGCCTTCCAGTATGAAGGCGCAGTAAAAGAGGATGGAAGGGGGCCTTCTGTATGGGACATTTTTTCACACACTTTTGGCAAAATAACTGATTTCAGTAATGCTGATGTTGCTGTGGATCAGT
ATCACCTTTATGATGGAGATGTACAACTTATGAAGGATATGGGAATGGATGCGTATAGGTTTTCGATATCCTGGTCTCGGATATTCCCCAATGGAACTGGGCAAATCAATCAGGCAGGGGTTGAT
CACTACAACAATCTCATTAATTCATTGCTAGCGAAAGGAATTGAACCGTACGTAACCCTCTACCACTGGGATCTTCCTCAAGCCTTGGAAGACAGATACACTGGGTGGCTTGACGCTCAAATCAT
ACAGGACTTTGCAGTATACGCGGAGACATGCTTTCAGAAATTCGGTGACAGGGTGAAGCACTGGATCACATTCAACGAGCCACACACATTTGCTGTACAAGGATACGATGTAGGTCTCCAGGCAC
CAGGAAGATGCTCTCTCCTAGGCCGCCTTTTCTGTAGAGCCGGAAACTCTGCAACTGAGCCTTACATAGTTGCTCACAATGTCATCCTTTCTCATGCAACTGTTGCCGACATTTACAGGAAAAAG
TATAAGCCAAAACAACGAGGATCAATTGGGGCATCATTTGATGTTATTTGGTATCGATCAGCAACAAACTCTACAGCAGACATTGAAGCAACTGAAAGAGCCCAAGATTTTCAGCTTGGCTGGTT
TCTTGACCCTTTCATTTTTGGGGATTATCCATTCTCTATGAGAAGTAGAGTCGGAAGCCGTCTGCCAAAGTTTTCCAAATCTGAGTCCACTTTAATCAAGGGGTCATTGGATTTTGTGGGCATTA

ATCACTACACTACCTTCTATGCCAGTAACGATTCTTCTCATATTATCGGACTACTGAATGACTCCCTATCAGACTCTGGTGCCATTGCCCTTCCTTTCAAAGATGGAAAAGCTATTGGAGATAAG
GCAAATTCGATATGGTTATACATAGTGCCTGAGGGGATGAGAAGCTTAATGAACTACATCAAGAAAAAGTATGGCAATCCTCCGGTGCTTATCACTGAGAATGGCATGGACGACCCAAATAGCCC
ATTCATTTCCCTCAAGGATGCTCTAAAGGATGAGAAAAGGATCAGTTACCACCGTGACTATTTGTCAAACTTGCTAGCTTCAATCAAAGAAGATGGGTGCAATGTGAACGGTTATTTTGCATGGT
CTCTACTGGATAACTGGGAGTGGGGTGCAGGATACACTTCAAGATTTGGTCTCTATTTCATTGACTATAAGGACAAGCTCAAGAGATACCCCAAGGACTCTGTTCAATGGTTCAAGAACTTCTTA
ACTTCAACTTGA

>XM_004295179.2_bg13
ATGGCGTCCATTTCCGGAGGCTCCTCTTTCCTCAACATTATCGCTATAAGCCTTATTGTTAGCGCTTTTGCCTCGTTAGTGAGAGGTGATGATCTGAGCAGGGGCCAATACTCGACTGCTTTTCT
CAACCGGAGCAGTTTTCCGGCTGGTTTCGTATTCGGCACAGCTTCATCAGCTTACCAGTATGAAGGTGCTGCAAATCTAAGTGGCAGAGGACCAAGTATATGGGATACTTTCACCCACAAATATC
CAGATAAGATAACAGATGGTAGCAACGGAGATGTTGCGATAGATTCATATCATCGCTATAAGGAAGATGTGGCGATTATGAAGGAAATGGGTTTGGATGTTTACAGATTTTCTATCTCATGGTCT
AGAGTGCTACCAACTGGGAAATTAAGTGGGGGTGTGAACAAGGAAGGGATAGAATACTACAACAACCTTATCAATGAACTCCTTGCCAATGGTATAAAGCCATTTGTGACACTCTTCCACTGGGA
TCTTCCCCAACCCTTGCAAGATGAGCATGGCGGCTTTATAAGCCCCCATATTGTGAAGCATTTTAAGGCATATGCAGAACTTTGTTATAGGGAATTTGGTGATCAGGTGAAGCACTGGATCACAT
TCAATGAGCCAATTGCCCTTGCCGTGGCTGGTTATGGACTCGGCGCATTGGCACCGGGACGATGTTCCGCTTGGATAAACCCCAACTGCACCGGAGGGAATTCAACGACGGAGCCATATCTGGTC
ACACATTACCAACTCCTTGCTCATGCAGCTGCAGTTAACTTGTATAAGAAACATTACCAGGAGTCCCAAAAAGGACTGATAGGGATAACCCTGGTGGCACAATGGTTGGTACCAAATACTACGGC
ACGGCACGACCGAGCTGCACAACTTAGAGGCCTGGATTTTATGCTTGGATGGTATATGGACCCTTTGACAAATGGTGACTACCCTAAGAGCATGAAATCTCTTGTCGGAGACCGATTGCCTAAGT
TTAAGAAAGAGCAATCCAAATTGCTGAAAGGATCGTTTGACTTTATCGGATTGAACTACTACACTTCTAACTACGTCTCCGACGCACCCCAGCTAGTTAAAGTCGTCAATGCAAGCTTCATGACA
GACTCTCTTGCTACTATGTCACCCCTTCGAAATGGAATCCCTATTGGTCCAAAGGCTGCCTCAGAGGACCTCTATATTTATCCGAGAGGAATCAGGGATATTTTGCTCTACACAAAGAGAAAGTA
TAACAATCCGCTTATTTATATCACAGAGAATGGCGTTGATGAGTTTAATGATCCCAAATTAACACTTGAGGAAGCCCTTGCTGACAACCAAAGAATTGACTACCACTTCCGCCATCTCTATTACC
TCCAAGAAGCGATCAAAGATGGTGTTAATGTGAAGGGCTACTTTGCATGGTCATTTTTGGACAACTTTGAATGGACATTAGGTTACACTGTTCGGTTTGGCATCAACTATGTCGATTATAAAGAT
GGGCTGAAAAGATACCCTAAACACTCAGCATTATGGTTCAAGAATTTCCTCAAGAAGTATTGA

>XM_011463607.1_bg13
ATGGCACTCCGATTAGGGTTAATCCTCCTCCTACTTGTTCTTGTCTGCACTGCAGCTGTGACAACCGGTAGAGCTGCCAGCTCGCGGCACCGTGACAGCTCTTCCCTCCACCGGAGCAGTTTTCC
GGCTGGCTTCGTATTCGGAACAGCTTCATCGGCTTACCAGTATGAAGGTGCTGCAAATGAAGGTGGTAGAGGTCCAAGTATATGGGATACATACACTCACAAGTATCCCGAGAAGATACACGACC
ACAGTACTGCAGATGTGGCTATTGATGAGTATCATCGCTATAAGGAAGATATCCGGATTATGAAAGATATGGGGCTTGATGCTTACAGATTTTCAATTTCATGGTCAAGATTGTTACCAAATGGA
AAGCTGAGTGGAGGTGTGAACAAGGAAGGAATCAAATACTACAATAACCTCATCAACGAGCTTCTAGCCAACGGTCTTGTACCCTATGTTACGCTCTTTCATTGGGATACCCCTCAAGCTCTGGA
AGATGAATTCAGTGGGTTTCTTAGCCCTAAAATTGTGGACCATTTCAAGGATTATGCAGATCTCTGCTATAAAGAATTTGGTGACAGAGTTAGGTATTGGATCACGTTAAATGAGCCATGGAGCT
ACACCAATGGTGGTTATGTTATTGGAACGTTTGCGCCAACCCGATGTTCTGATTGGCAAAATCTGAATTGTACCGGTGGAGATTCAGGGACTGAGCCATACTTGGCGACACATTATCAACTCCTG
GCTCATGCAGCAGCCGTGAAGTTGTACAGAGACAAATATCAGGCATCACAGAAGGGCGTGATCGGAATAACACTACTATCATACTGGTTTGTGCCATATTCTGATGCAAAGTGTGATGTGGATGC
TGCAGAACGTGCATTGGATTTTATGTATGGATGGTTTATGGACCCCCTGATTAACGGAGACTATCCACACAGCATGCGATATCTTGTTGCAGAACGATTACCAAAATTCACCAAAGAAGAATCCA
TGTTGCTTAAGGGGTCATTTGATTTCATTGGAGTAAACTACTACACTGCTAACTTCGCAGCCTACAATGCACCTCGGTTTAATGCTACTCATCCTAATTCAAATAACCCAAGCTACTTGACAGAC
GCTCTTGTCAATCTTTCAGTGGAACGTGATGGAGTTCCTATTGGCCCAAAGACTGCTTTGGATTGGCTCTATGTTTATCCAGAAGGATTTCGAGAGCTATTGCTCTACACAAAGAACAAGTATAA
TGATCCAGTTATCTACGTCACTGAAAACGGAGTTAATGAGCTAAACGATCCCGGATTATCACTCGAGGAATCCCTAACCGATCATCATAGAATTGACTATTACGATGCCCATCTCAGTTATCTGC
TTTCAGCAATGAAGGATGGGGTTAATGTGAAAGGATATTTTGCGTGGTCATTGTTTGATAACTTTGAATGGAATACGGGTTACACTTGTAGATTCGGCTTCAACTTTATAGATTACAGAAATGGA
CTCAAACGATACCCTAAACTCTCAGCTGGCTGGTTCAAGAATTTTCTTCAGAAGACGTCTGTTAAGTTAGATACAAGTTCCTCTCGAGAGTTTCAATCAATTTAA

>XM_004296295.2_bg13
ATGGCTCTCCGGTTAGGCGTAGGTACCCTTCTCTTTGGCCTCCTACTTGTTCTTAGCAGTACAGGGATAAACAGTCATAGTAGCCGTAAAACATCACCCCAAAAGAAGTCACGTAATGATACTTC
CTTCAGCCGCAGCAATTTTCCGGCAGGTTTCGTATTCGGAACAGCTTCCGCAGCTTACCAGTTCGAGGGGGCTGCAAAGGAAGGTGGTAGAGGACCATGTGTATGGGACGCCTTTGCTCACAAGT
ATCCAGAGAAGATAAAGGACCACAGCAGTGGAGATGTGGCTACTGATCAATATCATCGGTACAAGGAAGATGTGAAGATGCTTAAGGATATGGGTTGGGATGCTTACAGATTCTCAATCTCATGG
TCTAGGTTGTTACCAAAGGGAAAGCTAAGTGGGGGTGTGAACCAAGAAGGGATCACATATTATAAGAATCTCCTCAACGAGCTTGTAGCCAATGGTCTAAAGCCCTTCGTCACACTCTTCCATTG
GGATACGCCACAAGCTTTAGAAGAGGAATTCGGTGGTTTCCTAAGCCCTCAAATTGTGAATCGTTTCCAAGACTTTGCAGAGCTCTGCTACAAAGAGTTTGGTGATAAAGTCAAGCACTGGACTA
CACTAAATGAGCCACTTACCTACAGCGTCAGTGGTTATGCGACCGGGGCAATGGCACCAGGCAGATGCTCTGCTTGGCAAAACCCTAAATGTACCGGTGGCGATTCAGGGACAGAGCCATATTTG
GTGACACATCACCTACTTCTAGCTCACGCCGCCGCTGTGAAGGTGTACAAAGAGAAATTTCAGGGAAAACAGAAGGGTTTGATAGGGATAGTGCTAAACACAGGATGGTTTGTGCCATATTCTGA
CTCAAAGCGTGACCAAGATGCTGCACAACGAGCAATAGATTTCAGCTTAGGATGGCATATGGATCCCGTAGCGCATGGTGATTATCCACAAATCATGCGATCTCTTGTCGGAAATCGGTTACCCA
AGTTCACTAAAGAGCAATCACATATGCTAATAGGATCATTTGATTTCATTGGAATGAACTACTACTCCGGTGGTTATTCAGCGGATTTACCTAATAAACCTGATCCTAAGCAACCGAGCTACACT
TCAGACTCCCTCGTTAATTCTACATCCGACCGGAATGGTATCCCTATTGGTCCAGTGGCTGCTTCATCGTGGCTCCTTGTCTATCCTCAAGGATTTCTAGATTTAATGCTCTACATGAAGAAAAA
GTACAATAATCCAACTATGTATATTACTGAAAACGGAGTGGATGAGGTGAATGATCCAACATGGTCGCTTGAGAAAGCTCTTGACGATAGTAAAAGAATCGAATATTACGATCAACATCTTCAGA
AACTTCAGGCAGCAATTAAGGCTGGTGTTAAGGTGAAGGGATACTTTGCATGGTCATTCCTTGATAATTTTGAGTGGGCTGATGGTTATACTGTTAGATTCGGCCTCGTCTATGTGGATTTCAAA
AATGGGCTCAAAAGGCACCCTAAAAAGTCAGCAAATTGGTTCAAGAATTTTCTTCAGAAATCTACAAAATCAGGAAAGTAA

>XM_004295180.2_bg12
ATGGCTCTCCGGTTAGGCGTAGGAACCCTTCTCTTTGGCCTGCTGCTTGCTGTTAGCAGTAAAGGGATAACTGGTCATAGTAGCCGTAAAGCTTCCCACAAAAGGATGTCACATAACGATTCTTG
CTTAAACCGCAGCACTTTTCCGGCAGGTTTTATGTTCGGGACAGCTTCGGCGGCTTACCAGTTCGAAGGGGCTGCAAATGAAGGTGGTAGAGGACCATGTGTATGGGACGCCTTTACTCACAAGT
ATCCAGAAAAGATAAAGGACCGCAGCAGTGGAGATATGGCTGCTGATCAATATCATCGGTACAAGGAAGATGTAAAGATGCTGAAGGAAATGGGTTGGGATGCTTACAGATTCTCAATCTCATGG
TCTAGGTTGTTACCAAATGGAAAGCTCAGTGGGGGTGTGAACCAGGAAGGAATCAAATATTATAAGAATCTCATCAACGAGCTTGTATCCCATGGTCTAATTCCCTTCGTCACACTCTTTCATTG
GGATACGCCCCAAGCTTTAGAAGAGGACTATGGCGGTTTCCTGAGCCCTCAAATAGTGAAACATTTCCAGGACTATGCAGAGCTCTGCTACAAAGAATTCGGTGATAAAGTCAAGCACTGGACAA
CACTAAATGAGCCGCATACCTACAGCGTCAATGGTTACGCAACCGGTGGATCGGCACCAGGCAGATGTTCTGCTTGGAAAGATCCTAACTGTACCGGTGGCGATTCAGGGACTGAGCCGTATTTG
GTGTCACATCACCTACTTCTAGCCCATGCCGCCGCTGTGCAGGTGTACAAAGAGAAATTTCAGGCGAAACAGAAGGGTTTGATAGGTATAGTGGTAAACACAGGATGGTTTATGCCATATTCTGA
CTCGAAGCGTGACCACGATGCTGCACAACGAACAATGGATTTCAACTTGGGATGGTTTATGGATCCCCTAACACATGGTGATTATCCACAAATCATGCGATCTCTCGTCGGAAATCGATTACCCA
AGTTCACTAAAGAGCAATCAGATAAGCTAAAAGGATCGTTTGATTTCATTGGAATGAACTACTACTCCGGTGGTTATTCAGCGGATTTACATACTAAACCAGATCCTAAGCAACCAAGCTATACG
TCAGACTCCCTCGTTAATGTTACATTCGAACGGAATGGTGTCCCTATTGGTCCAGTGGCTGCTTCATCGTGGCTCCTTGTTTTTCCTCAAGGATTTCTAGATCTATTGCTCTACATAAAGAACAA
GTACAATAATCCAACTATGTATATCACTGAAAACGGATGGGATGAGGTGAATGATCCAACATGGTCACTTGAGAAGGCTCTTGATGATAGTACAAGAATCGAATTTTACGATCAACATCTTCGGA
AACTTCAGGCAGCAATTAAGGCTGGCGTCAAGGTGAAGGGATACTTTGCATGGTCATTCCTTGATAACTTTGAGTGGGCTGATGGTTACACTGTTAGATTTGGCCTCGTTTATGTGGATTATAAA
AATGAGCAGAAAAGACACCCTAAAAAGTCTGTAAACTGGTTCAAGAATTTTCTTCATAAGTCTAAAGAATGA

>XM_011463606.1_bg12_x2
ATGGCACTCCAAGGCATCCTCCTCTTGGGCTTCCTTCTTGTTCTTGGCTGTGCTGTGAGGATCAGTGGAGCGACTAACTCTAAGCAATTTGACAATGCCTCCCTCAACCGGAGCAGTTTTCCGGC
AGGATTCATATTTGGGACAGCTTCTGCAGCTTACCAGTATGAAGGGGCTGCAAAAGAAGGGGGTCGAGGACCAAGTATATGGGACACATACACTCACAAGTATCCAGAGAAGATCTTTGACCACA
GCAGCGGAGATGTGGCTATTGATCAATATCATCGCTATAAGGAAGATGTTCGGACTATGAAGGACCTAGGTTTTGATGCTTACAGACTCTCATTTTCATGGTCCAGATTGTTACCAAATGGAAAG
CTAAGTGGGGGTGTCAACAAGGAAGGAATCAAATATTACAACAACCTCATCAACGAGCTTCTAGCCAATGGTGTGAAGCCTTTTGTCACAATCTTTCATTGGGATCTTCCCCAAGCATTAGAAGA
TGAATACGGTGGCTTCTTAAGTCCAAAAATTGTGAACCATTTTCAGGACTATGCAGAGCTCTGCTTTAAAGAATTCGGTGACAGAGTTAAGCATTGGATCACCTTAAATGAGCCCCATAGCTACA
GCCGTAATGGTTATGCAACCGGAAAGTTTGCACCAGGTCGATGTTCTGCTTGGCAGAAACGAAATTGCACAGGTGGGGATTCAGGGACCGAGCCATACTTAGCGTCACATTACCAACTTCTAGCT
CATGCAGCCGCCATGAAGTTGTATAGGCATAAGTATCAGGCAAAACAGAAGGGATTGATAGGGATAACAATAAATTCACACTGGTTTGTGCCGTATTCTGATTCTAAGCAAGACCAAGCTGCAGC
ACTACGAGCACTGGATTTTATGTTTGGATGGTATATGGATCCCATGACAAATGGTGACTATCCAAGTAGCATGCGAGCTCTTGTTGGAAATCGATTACCCAAGTTCACGAAAGAGCAATCAAAGA
TGCTGAAAGGTTCATTTGATTTTCTTGGCTTGAATTACTACACAGGTAATTATGCAGCCAATATACCGTTTCGTAATGTTAAAGAAGCAAGCTACACAACAGACTCTCTTGCTAATCTTACAGCC
GTGCGTAATGGTATTCCAATTGGTCGACCGGCTGCTTCAGATTGGCTTCATTTTTATCCACAAGGTTTTAGAGAGATATTGCATTACACAAAGAAGAAGTACAACAATCCTCTTATTTACATCAC
TGAAAATGGATGGGATGACCGCGGTGATCCAACATTATCACTAGAGGAAGCCCTTCGCGATAATCTAAGAATTGAGTATTACAATCGCCATCTTGATGAACTTCAGAAAGCAATTAAGGCTGGGG
TTAAGGTGAAGGGGTACTTTGCATGGTCATTGTTTGATAACTTTGAGTGGGCTGATGGTTATAGTTGTAGGTTTGGTATCAATTCTGTGGACTACAAAAATGGACTCAAAAGACACCCTAAACAC
TCTGCAGCTTGGTTCAAGAACTTTCTGCAGAAGTAG

>XM_011463605.1_bg12_x1
ATGGCACTCCAAGGCATCCTCCTCTTGGGCTTCCTTCTTGTTCTTGGCTGTGCTGTGAGGATCAGTGGAGCGACTAACTCTAAGCAATTTGACAATGCCTCCCTCAACCGGAGCAGTTTTCCGGC
AGGATTCATATTTGGGACAGCTTCTGCAGCTTACCAGTATGAAGGGGCTGCAAAAGAAGGGGGTCGAGGACCAAGTATATGGGACACATACACTCACAAGTATCCAGGGAAAAAATCAGAGAAGA
TCTTTGACCACAGCAGCGGAGATGTGGCTATTGATCAATATCATCGCTATAAGGAAGATGTTCGGACTATGAAGGACCTAGGTTTTGATGCTTACAGACTCTCATTTTCATGGTCCAGATTGTTA
CCAAATGGAAAGCTAAGTGGGGGTGTCAACAAGGAAGGAATCAAATATTACAACAACCTCATCAACGAGCTTCTAGCCAATGGTGTGAAGCCTTTTGTCACAATCTTTCATTGGGATCTTCCCCA
AGCATTAGAAGATGAATACGGTGGCTTCTTAAGTCCAAAAATTGTGAACCATTTTCAGGACTATGCAGAGCTCTGCTTTAAAGAATTCGGTGACAGAGTTAAGCATTGGATCACCTTAAATGAGC
CCCATAGCTACAGCCGTAATGGTTATGCAACCGGAAAGTTTGCACCAGGTCGATGTTCTGCTTGGCAGAAACGAAATTGCACAGGTGGGGATTCAGGGACCGAGCCATACTTAGCGTCACATTAC
CAACTTCTAGCTCATGCAGCCGCCATGAAGTTGTATAGGCATAAGTATCAGGCAAAACAGAAGGGATTGATAGGGATAACAATAAATTCACACTGGTTTGTGCCGTATTCTGATTCTAAGCAAGA
CCAAGCTGCAGCACTACGAGCACTGGATTTTATGTTTGGATGGTATATGGATCCCATGACAAATGGTGACTATCCAAGTAGCATGCGAGCTCTTGTTGGAAATCGATTACCCAAGTTCACGAAAG
AGCAATCAAAGATGCTGAAAGGTTCATTTGATTTTCTTGGCTTGAATTACTACACAGGTAATTATGCAGCCAATATACCGTTTCGTAATGTTAAAGAAGCAAGCTACACAACAGACTCTCTTGCT
AATCTTACAGCCGTGCGTAATGGTATTCCAATTGGTCGACCGGCTGCTTCAGATTGGCTTCATTTTTATCCACAAGGTTTTAGAGAGATATTGCATTACACAAAGAAGAAGTACAACAATCCTCT
TATTTACATCACTGAAAATGGATGGGATGACCGCGGTGATCCAACATTATCACTAGAGGAAGCCCTTCGCGATAATCTAAGAATTGAGTATTACAATCGCCATCTTGATGAACTTCAGAAAGCAA
TTAAGGCTGGGGTTAAGGTGAAGGGGTACTTTGCATGGTCATTGTTTGATAACTTTGAGTGGGCTGATGGTTATAGTTGTAGGTTTGGTATCAATTCTGTGGACTACAAAAATGGACTCAAAAGA
CACCCTAAACACTCTGCAGCTTGGTTCAAGAACTTTCTGCAGAAGTAG

>XM_011463603.1_bg12
ATGGCATTTCAAGGCACTCTCAACTTTGTAATCTTACTTGTTTTTAGCTATGGATTGGCAACTAGTATAGCTACCTCCCCTAGCCATTACGATGTTACTGCCATCAACCGGAGCACCTTTCCGGC
AGGTTTCATATTCGGGACAGCTTCTTCGGCTTACCAGTACGAAGGTGCTGCAAAAGAAGATGGAAGAGGACCAAGCATATGGGACACATACACTCACACATTTCCAGATAAGATAAAGGACGGCA
GCAATGGAGATGTCGCTATTGATGCATATCACCGCTACAAGGAGGATGTTGGCATCATGAAAGATATGGGTTTTGATGCTTACAGATTCTCCATCTCGTGGTCTAGATTGTTACCAAATGGAACG
CTAAGTGGGGGTGTGAATAAGGAAGGAATCAAATACTATAACAACCTCATCAACGAGCTTCTAGCCAATGGTCTAAAACCCTTTGTCACCCTCTTCCATTGGGATCTTCCTCAAGCTTTAGAAGA
TGCATACGGTGGATTCTTAAGCCCTCAAATTGTAGATCATTTCCAAGACTACGCAGAGCTATGCTTTAAGGAATTTGGTGATAGAGTTAAGGACTGGATCACATTAAACGAGCCATGGAGCTACG
CCCGTGGTGGTTATGTAATCGGGACGTTTGCACCGTGCCGATGTTCTGATTGGCAAAACCTGAATTGCACCGGTGGAGATTCAGGAACAGAGCCATATTTGGCCTCACACTACCAGCTTCTAGCT
CACGCAGCCGCCGTAAAGTCATACAAAGAGAAGTATCAGGCTGAGCAGAAAGGTGTGATAGGGATAACAATACTTTCCCACTGGTTTGTGCCCTTTTCTGATGCCAAGCACGATAAAGAAGCTGC
TCTACGAGCATTGGATTTTATGTTTGGATGGTATATGGATCCCTTAACATATGGTGAATATCCGCACAGCATGAGATCTCTTGTCGGAGATCGGTTACCGGAGTTCACGAAAGAGCAATCCGAGA
TGTTAAAAGGTTCATTTGATTTTCTAGGATTGAATTACTACACCGCTAACTATGCTACTTACGCACCTGAACTCAAGAATGCTGCAAACCCTAGCTACCTTACAGATGCTATCGCTATTCTTTCC
ACCGAGCGTAATGGTATTCCTATTGGTCCACAGTCTGCTTCAGATTGGCTCTATGTTTATCCAGAAGGAATTCGAGAACTTTTGCTTTACACGAAGGAAAAGTACAATGATCCACTTATTTACAT
CACTGAAAATGGAAGGGATGAGCACAACGATCCCAAATTAACACTTGAGGAAGCCCTAGCCGATACTCACCGAATCGACTTTTACTATCAACATCTTCATTATCTTCATGAAGCGATCAAGGATG
GCGTTAACGTGAAGGGATATTTCGCATGGTCATTGTTTGACAACTTCGAATGGAATATGGGTTACAGTGTTAGGTTTGGCATCAACTATGTGGATTACAATGATGGCCTCAAGAGATACCCTAAA
CTGTCCGCACACTGGTTCAAGAATTTTCTTGGAAACTAG

>XM_011463602.1_bg12
ATGAAAATCCAGGGGTCTTTGCTTCCAGCCATAGTGCTTCTGCTTCTTGGCTTTGGATTGAGAAATGGTAAAGCCAGTGATACACCCCCTGCTCATTATGACACCGCTTTCCTCAACAGGACTAG
TTTCCCAGCAGGTTTCATATTTGGGACAGCTTCATCATCTTACCAGTATGAAGGTGCTGCTCAAGATGGTGGCAGGGGACCAAGCATATGGGATACCTATACCCACAGATATCCAGACAAGATTA
AGGACGGTAGCAATGGAGATGTAGCTAATGATGAATATCACCGCTATAAGGAAGATGTAGGGATTATGAAGAACATGGGGTTGGATGCATATCGGTTCTCTATCTCATGGTCCAGATTGTTACCA
AATGGAAAGCTAAGTGGGGGAGTGAACAAGAAAGGAGTCAAATACTACAACAATCTCATCAATGAACTCCTACGCAATGGCATAAAACCATTTGTGACCCTCTTCCACTGGGATCTTCCCCAAAC
TTTAGAAGATGAGTATGGCGGTTTTTTAAGCCCTCACATTGTCAATCATTTTAGGGACTACGCAGAGCTTTGTTACAAGGAATTTGGTGACAGGGTAAAGCACTGGATCACGTTTAATGAGCCAT
ATAGTTATAGTGCTGGTGGTTATGCAGCGGCAATTTTGGCACCAGGACGCTGTTCTGATTGGCAACAGCTAAACTGCACCGGTGGGGATTCCGGTACTGAACCATATTTGGTAGCACACCACCAG
CTCCTTGCTCATGCAGCTGCAGTAAAATTATACAGGCAAAAATATCAGTCATCTCAAAAGGGATTGATAGGGATTACTCTAGTTTCAAATTGGTATGTGCCGGTTTCGGAGGCAAAGCACCATAA
AAATGCTGCACTAAGAGCTCTAGATTACATGCTTGGATGGTTTATGGATCCCTTGACAAATGGAGATTATCCACACAGCATGAGGTCTCTAGTTGGAAACCGATTACCAAAATTCACAAAGGAGC
AGTCCAAATTGTTAATTGGATCATTTGATTTTCTGGGATTGAATTATTACACAGCTAATTATGCAGCCTATGCACCTCAACATAATTCTGTCAATGCAAGCTACTTAACAGATGCTCGTGCTTCT
CTTTCATCTGTGCTTAATGGAATTCCCATTGGACCAAAGGCTGCTTCAGACTGGCTATACGTTTATCCAAGAGGAATTCGAGATCTTTTGCTCTACACAAAGACAAAATATAATGATCCACTCAT
ATACATTACTGAGAATGGTATTGATGAGTACAATGATCCGCAATTATCACTTGAAGAAGCCCTTAACGACACGCAGAGAATCGACTACTACTATCGCCACCTGTATTACCTTCAAAGAGCAATCA
AGGATGGTGTGAATGTGAAAGGATACTTTGCATGGTCACTGCTAGACAATTTTGAATGGAATTCTGGTTATACAGTCCGATTTGGAATCAACTATGTAGATTACAAAAATGGGCAGACTAGGCAC
CCAAAACTTTCTGCACACTGGTTCAAAGGTTTTCTGAAGAAAACGTAA

>XM_004295175.2_bg12
ATGGCAATGCAAAGATTTTTGTTTCTAGGCATAGTGCTTCTACTTGTTGCAGGCTTTAGATTGACAAACAGTGAAGCTCTTTCACCCAGTAGGACTCCTATCTATGACACTGCTTTCCTCAACAG
GACTAGTTTCCCTGAAGGTTTCGTATTTGGGACAGCTTCAGCGGCTTATCAGTATGAAGGTGCTGCTAAAGAAGGTGGTAGAGGACCAAGCATATGGGATACCTACACCCACGAACATCCAGAAA
GGATCACGGATGGAAGCAATGGAGATGTAGCTAATGATCAATATCACCGCTATAAGGAAGATGTGAGGATTATGAAGGATATGAATCTGGATGCTTATAGGTTCTCTATCTCATGGTCAAGACTG
TTACCAAATGGAAAGCTAAGTGGGGGAGTGAACAAAGAAGGAGTCAAGTACTACAACAATCTCATCAATGAACTCCTAAGCAAAGGTCTACAGCCATATGTGACCCTCTTTCACTGGGATCTTCC
CCAAACTTTAGAAGACGAATATGGCGGTTTCTTAAGCCCTAACATTGTCAACCATTTTCGGGACTTTTCGGAACTGTGTTTCAAAGAATTTGGTGATCGGGTGAAGTACTGGATCACGTTGAATG
AGCCATGGACATACAGCAATGGTGGTTATGCCCTCGGTTCGTTGGCACCGGGAAGGTGTTCTGCTTGGCAGCAGCTAAACTGCACGGGAGGGGATTCGAGTACTGAACCCTATTTGGTGGCACAC
AACCTACTCCTTTCTCATGCAGCTGCTGTAAAGTTGTACAGGCAGAAATATCAGGCATATCAGAAGGGAGTTATAGGAATTACCATAGTGTCTCACTGGTTCATTCCAATTTCAGACGCAACGCA
CCACAAAAATGCCGCCTTACGAACTCTGGATTTTATGTTTGGATGGTTCATGGATCCTTTGACAAACGGAGACTATCCACACAGCATGCGATCTCTCGTCGGAAACCGATTACCGAAATTCACAA
AAGAACAATCCAAGTTGCTAATCGGATCATTTGATTTTCTTGGACTGAATTACTATACTACTTACTATGCAAGTTATACACCTCAGAACAATTCTTTGAATGCAAGCTACGTGACAGACGCTCGT
TCTACTCAATCATTTGCGCTCAATGGCGTCCCGATTGGTCCACAGGCTGCTTCATCATGGCTATATGTTTATCCAAAAGGACTTAGAGACCTTTTACTCTACACAAAGACGAAGTATAATGATCC
TCTTATTTACATTACTGAGAATGGCATCGATGAGTTCAATGATCCGAAATTATCACTTGAGGAAGCCTTGTTTGACAATCAGAGAGTTGACTATTACTTTCGCCATCTCTATTACCTTCAAAAAG
CAATCGAGGATGGTGTGAAAGTGAAGGGCTACTTTGCATGGTCATTGCTAGACAACTTCGAGTGGGCTTCGGGTTACACAGTGCGGTTCGGTATCAACTACGTGGATTACAAGAACGGAAACAAA
AGGCACCCAAAGCTCTCAGCACACTGGTTCAAAAACTTCCTCAAGAATTACTGA

>XM_004294358.2_bg11
ATGGTTCTGCAACTGGAACTGCTTCTCTTTCTCTCCTTCATAACTCTTATCATCGGTATCCCCCTAAATCTTTCTTTAGATTTAGAGTTAGGCGCTGGTGATCATGGGTTCAAAAGAGCCGACTT
CCCAGCCCCACCGGACTTTGTGTTTGGTGCTTCCTCCTCTGCTTATCAGGTGGAAGGTGCTGCAGACCAAGACGGGAGAACCCCTAGCATCTGGGACACCTATGCGCATGCTGGAAAAATGCATG
GAGCCACTGGAGATGTAGCGTGTGATGAGTATCATAGATACAAGGAAGATGTGCAACTAATGGTGGATACTGGTCTAGAGGCGTATAGATTTTCCATCTCATGGTCAAGACTTATATCCAATGGA
AGAGGACCTGTGAATCCAAAGGGTCTACAGTATTACAACAATCTCATTGACCAACTAATCAGCAAAGGAATCCAGCCTCATGTTACCTTACACCACGCTGATCTCCCACAGGCACTCGACGATGA
GTATGGAGGATGGGTTAGCCCTCAGATTATAAAAGACTTCACTGCATATGCAAATGTTTGCTTCGCACAGTTCGGCGATCGAGTTAAGCACTGGACTACTATGAACGAGCCAAATGTATTTGTAC
TCGGGGGTTATGATATTGGATTCCTTCCACCGTCACGATGTTCAGCACCATTTGGTGTTAACTGCTCGAGGGGTGACTCCACAACGGAACCATACCTGGCAATTCATAATTTCTTGCTATCTCAT
GCATCGGCTGCTGCATTGTACAAGCAAACCTACCAGCACAAGCAGTGTGGGTTTATAGGCATCAATGTATTTTCCTATTGGTTTATTCCTCGAACGGAAACCCATCAAGACCAAGCTGCTGCTCA
AAGAGCTATGGACTTCTACTTCAGTTGGGTGGTGCATCCTTTGGTGTATGGAGAGTATCCTGGAGTAATGAAAAAAAATGCAGGCTCTAGACTTCCGACGTTCACCAGTGCTGAGTCTGCACTGG
TCAAGGGATCCTTCGACTTCATCGGACTCAATTACTATAACACATTGTATGCCGATGACAACTCTGCCGCCCTGAACCTTCAAACAAGAGACTATGTTGCTGATTCGGCAATCAAGATTTCTCCT
CTGGATCAGGATAGATCAACGTTTGAGTATCCGATAACACCTTGGGGTATGGAAGAACTGTTGGAATATATCAAACAAAACTACGGCAATCCTCTCTTATACATCCACGAAAATGGACAACGAAC
TCGACGCAATTCATCGTTAGAGGATTGGTCCAGAATCGAATATTTGCACGGACACATTCACAGTTTGCTTAATGTTATTAGGAATGGATCAAATGCCAGAGGCTATTTTACCTGGTCTTTCTTGG
ATACACTGGAGCTTTTGGATGGCTTAGAATCAAGCTACGGCCTTTACTACATCGATGTGGATGATCCAGATTTAAAAAGACAGCCTAAACTCTCTGCTCATTGGTACTCAAAGTTTCTAAAGAAT
AAAAACTTCACTCCACGTTCAGATCCTGATGCTGATCAACATTATTTCTTTCAGTAA

>XM_004294357.2_bg11
ATGTTGAGAGTGGTTCTGCAACTGGAACTGCTCCTCTTTCTTTGCTTCGTAATTCTTCTCAGTATCCCCCTAAATCTCTCTTTAGCTTCCGAGTTAGAATTAGGCGCTGGTGATGATGATGAGGT
TAAAAGAGACGACTTCCCTCCCCCGCCGGACTTTGTTTTTGGCGCTTCCTCCTCTGCTTATCAGGTGGAAGGTGCTGCAGACCAAGACGGGAGAACCCCAAGCATCTTGGACACCTATACGCATG
CTGGGAAAATGCATGGAGCAACTGGAGATATAGCGTGTGATCAGTATCACAAATACAAGGAAGATGTGCAACTAATGGTGGATACTGGTTTAGAGGCATATAGATTTTCCATCTCATGGTCAAGA
CTTATACCCAATGGAAGAGGACCTGTCAATCCAAAGGGTCTACAATATTACAACAATCTCGTTGACCAATTAATCAGCAAGGGAATCCAGCCTCATGTTACTTTACACCACAGTGATCTCCCACA
GGCACTCGACGATGACTATGGAGGATGGATTAGCCCTCAGATTATCAAAGACTTCACTGCATATGCAAATGTTTGCTTCGAACAGTTTGGAGATCGAGTTAAGCACTGGACTACTATGAATGAGC
CAAATGTGTTTGTACTCGGGGGTTATGATATTGGATTCCTTCCACCGTCACGATGCTCAGCTCCATTTGGTGTTAACTGCTCGAGGGGTGACTCCACAACGGAACCATACCTGGCAATTCATAAT
TTCTTGTTATCTCATGCATCGGCTGCTGCACTGTACAAGCAAACCTACCAGCACAAGCAGCGTGGGTTTATAGGCATCAATGTACTTGCATTTTGGTTTATTCCTCGAACCGAAACCCATAAAGA
CCGAGCTGCTGCTCAAAGAGCTATGGACTTCTACTTCGGTTGGGTTGTGCATCCTTTGGTGTATGGAGAGTACCCTCAAGTAATGAAAAAGAATGCAGGCTCTAGACTTCCGACGTTCACCAGTG
CTGAGTCTGCACTGGTCAAGGGCTCCTTGGACTTCATCGGACTCAATTACTATTACACGCAGTACGCCAAGGACAACTCTGCCGCCCTAAACATTCAAACAAGAGACTACGTAGCTGATTCGGCA
ATCCAGATTTCTCCTTTGGATCACAATACATCAACATTGGAGTTTCCGATAACACCATGGGGTATGGAAGGACTGTTGGAATATATCAAACAAAACTACGGCAATCCTCCCTTGTATATCCATGA
AAATGGCCAACAAACTCGACGCAATTCATCGTTGGAGGATTGGTCCAGAATCGAATATTTGCGTGGCCACATTCACAGTTTGCTTAATGTTATAAGGAATGGATCAAATGCTAGAGGCTATTTTA
TGTGGTCCCTATTGGATTCAATTGAGCTTTTGGGTGGCTATGAAATAAGCTACGGCCTCTATTACATCGATTTGGACGATCCTGATTTAAAGAGACAGCCTAAACTCTCTGCTCATTGGTACTCA
AAGTTTTTAAAGAACAAAAACATCACTCCACGTTCAGATTCATCAGATACTGATCAACTTTACTTCTTTCAGTAG

>XM_004294274.2_bg41
ATGAAACTATGCATCATCCTGTTACTGATAACCAGGCTCTTTACAATCTCTCAATCTCTTAGCAGAGCTGATTTTCCTGAAGGGTTCGTTTTCGGAACAGCTTCTTCGGCATACCAGTTTGAGGG
AGCTGTGAATGAAGGAAACAAGGGAGCTAGTGTATGGGATACTTTCACAAAAGTACCAGGGAAGATTTTAGATTTCAGCAATGCAGACACAGCTGTTGATCAATATCACAGATTTCAGGGTGATG
TAGACTTGATGAAGGATATGGGAGTAAATGCGTACAGATTTTCAATATCGTGGCCACGAATTTTTCCAAATGGAACAGGAGCACCCAACTCAGAAGGAATAAGCTACTACAATAGCCTTATAGAT
TCTTTACTTGAAAAAGGGATCCAGCCTTTTGTAACTCTATATCATTGGGATCTTCCGCAGATGCTTGAAGACAAATATGAAGGATGGTTGAGCACACAGATAGTAAAAGACTTTGAACATTATGC
CATCACCTGCTTCCAAGCTTTTGGAGATAGAGTTAAGAACTGGATCACCTTCAACGAGCCTCATGGTTATGCACTTCAAAGTTATGACTTGGGAATACAAGCTCCCGGACGATGTTCTATTCTTG
GTCACTTGTTTTGTAAGAAAGGAAATTCATCTGTTGAACCATACATTGTCGCTCACAACATCCTCCTGTCCCATGCTGCTACTTATCATACATACCAGCAATATTTCAAGGAGAAACAAGGAGGT
CAGATTGGAATTTCATTGGATGCCAAATGGCATGAACCTCTCTCGGAAACTGATGAGGATATAGATGCAGCACATAGAGCGAATGAGTTCAGTTTGGGATGGTTCCTTGATCCACTATTTTTTGG
CGATTATCCTCTCTCAATGAAGAAGCTTGTGGGTAAGCGATTACCTGCAATTTCGAAGGAAGTTTCAAAATTCCTCGTGGGATCAGTGGACTTTATCGGCATAAACCACTACACCACATTATATG
CTCGGAATGACAGGACACGAATTCGAAAATTTATATTGCAGGATGCTACATCTGATGCTGCAGTCATTACAACACATTACAAAGGAGGAGTGGCTATTGGAGAAAAAGCAGCTTCTCGCTGGTTG
CGCATAGTCCCATGGGGAATCCGAAAGTTGGCAAGATACATCAAAGATAAGTATGGGAACCCCCCAATGATGATAACAGAAAATGGTATGGATGATCCAAATAAACCTTTCACATCTATGGAAAA
GGCTCTAAATGATGAGAAGAGGATACGTTTTCACAGGGACTATCTCTCAAACCTATCAGCTGCCATAAGGGAAGACAAATGTGATGTTCGTGGTTATTTCGTGTGGTCATTACTTGACAACTGGG
AATGGAACATGGGCTACACTGTGAGATTTGGCCTCTACTATGTCGATTACAAGAAGAACCTTACGAGGATTCCTAAATCTTCTGTACAGTGGTTTACAAGATTTCTCCAAGTTAACAACAATCAA
CAGAGAAGCCAGACCTTGCTAAATACTTCTGTACAGATATAG

>XM_004293223.2_bg47
ATGGAGGTTTTATCAGTATCTCATGCTTTCCTTATGACTGAGATCATGTGTTTTTCGATTCTTGTTGTGTCATGTAATCACATATCCTTGAAAGGAAACTCCCAGAGCAACTTTCTCTTTGGAAC
CGCATCCTCTTCTTATCAGTTTGAGGGAGCTTTTCTGAATGATGGTAAAAGTCTCAACAACTGGGATGTCTTCAGTCACAAGCCTGGTAATATCCTGGATGGAACTAATGGAGACATTGCTGTTG
ATCAGTACCATCTTTATCAGGAAGACTTGGATCTCATGGATTACATTGGAGTCAACAGCTACCGTTTTTCTATATCATGGGCAAGAATTTTACCCAAAGGAAGATTTGGAAAAGTGAACAGAGCT

GGCATTGATCACTATAACAAGCTCATCGATGCTCTTCTGCTTAGAGGAATCCAGCCCTTTGTGACATTGACTCACTACGACATACCTCAGGAACTTGAAGACAGATATGGAGCTTGGCTAAGTCC
TCTACTGCAGGAAGATTTTAGATATTATGCAGATATATGTTTCAAATTCTTTGGAGACAGAGTCAAGTACTGGTCTACTTTCAACGAGCCCAATGTTCTGGCCATTCGTGGATATCGGTCTGGGA
TTTACCCACCGTCTCATTGCTCTCTCCCTTTTGGGAATTGTATTAGTGGAGATTCAGAAAGAGAGCCTTTCATTGCAGCTCATAACATCATTCTATCCCATGCAGCCGCTGTGAACGTATACCGA
ACCAAATATCAGAAAAAACAAGAAGGGAGCATTGGAATAGTGATGAATGCTGTATGGTATGAACCCATCAGCAGCTCTTTAGAAGACAAATTAGCAGCTGAAAGGGCTCAGTCTTTCTATATTAA
CTGGTTCTTAGATCCAATTGTACATGGAAGATACCCTGCAGAAATGCATGAGCTTCTAGGAGATGATTTGCCTGAGTTTTCAATGTTTGATATGGAGATGCTGAAGAATGGACTAGACTTCATTG
GTATCAATCACTACACAAGTTTTTACTCAAAAGACTGTATGATTTCTCCATGTGAACCAGGACCAGGAGTTTCAAGGACAGAGGGTTATGCTCTCAGAACTGCACAAAAAGATGGAATCTACATT
GGAGAGCCAACTGCAGTTGATTGGCTATATGTGCATCCTCAAGGAATGGAGAAGATGGTAACATACATAAAAGAGAGATACAATAACACACCAATGTTTATTACAGAAAATGGGTATGCTGAGGA
GGCAAACTCAAATACCACAAATGAAGAACAGTTCAATGATGTCAAGAGAGTAGAGTACATGAGAAGTTACTTACAGGCACTAGCAGACACAATGAGAACTAAAGGAGCAGATGTAAGAGGGTATT
TCGTGTGGTCATTGCTCGACAATTTTGAGTGGACTAGTGGATATACGGTACAGTTTGGACTTCACCATGTTGATAATGTCACTCTCAAGAGAACTCCAAGACTCTCTGCAGATTGGTACAGACAA
TTTATCGTTAATAACACCCTGCAGATTTTAAGTGCCCAGGAATGA

Supplementary Table S1. Maximum reaction velocities (V_{max}, μmol s^{-1}) of Pru-*β*GL and Fra-*β*GL towards kaempferol 7-*O*-glucoside, luteolin 7-*O*-glucoside, quercetin 7-*O*-glucoside, and *p*-nitrophenyl-*β*-D-glucopyranoside. Values are presented as mean ±SD.

**Substrate**	***V*max (μmol s⁻¹)**
Kaempferol 7-*O*-glucoside (*P. dulcis*)	0.176 ±0.008
Kaempferol 7-*O*-glucoside (*F.* × *ananassa*)	0.208 ±0.031
Luteolin 7-*O*-glucoside (*P. dulcis*)	0.276 ±0.031
Luteolin 7-*O*-glucoside (*F.* × *ananassa*)	0.203 ±0.013
p-Nitrophenyl-*β*-D-glucopyranoside (*P. dulcis*)	7.391 ±0.211
p-Nitrophenyl-*β*-D-glucopyranoside (*F.* × *ananassa*)	608.758 ±11.821
Quercetin 7-*O*-glucoside (*P. dulcis*)	0.032 ±0.002
Quercetin 7-*O*-glucoside (*F.* × *ananassa*)	0.034 ±0.003

Supplementary Table S2. pH dependence of Fra-βGL and Pru-βGL activity at 30 °C. Measurements were performed in triplicate, and the values shown represent the mean relative activity. For each enzyme, the highest activity measured within the tested pH range was defined as 100%, and activities at the remaining pH values were expressed relative to this maximum.

pH	Pru-βGL % activity	Fra-βGL % activity
**3**	9.54	9.49
**3.5**	41.23	10.7
**4**	53.58	61.96
**4.5**	100	94.82
**5**	92.94	100
**5.5**	90.7	87.89
**6**	60.73	51.87
**6.5**	47.03	28.95
**7**	6.73	16.08
**7.5**	8.6	8.35

Supplementary Table S3. Temperature dependence of Fra-βGL and Pru-βGL activity at pH 4.5. Measurements were performed in triplicate, and the values shown represent the mean relative activity. For each enzyme, the highest activity measured within the tested temperature range was defined as 100%, and activities at the remaining temperatures were expressed relative to this maximum.

Temperature °C	Pru-βGL % activity	Fra-βGL % activity
20	43.12	14.6
30	71.17	30.43
40	100	50.23
50	85.06	83.41
60	76.91	100
70	74.43	72.61
80	63.21	35.69

Supplementary Table S4. Thermal stability of Fra-βGL and Pru-βGL at pH 4.5. Measurements were performed in triplicate, and the values shown correspond to the mean residual activity. An untreated sample maintained at room temperature served as the non-heated control and was defined as 100% activity. Residual activities following thermal treatment were expressed relative to this control.

**Temperature °C**	**Pru-βGL % activity**	**Fra-βGL % activity**
control	100.00	100.00
30	96.84	76.51
40	64.49	53.80
50	55.16	35.75
60	17.88	11.40
70	1.48	0.00
